# A pharmacological network for lifespan extension in *Caenorhabditis elegans*

**DOI:** 10.1111/acel.12163

**Published:** 2013-11-13

**Authors:** Xiaolan Ye, James M Linton, Nicholas J Schork, Linda B Buck, Michael Petrascheck

**Affiliations:** 1 Division of Basic Sciences, Fred Hutchison Cancer Research Center, Howard Hughes Medical InstituteSeattle, WA, USA; 2 Department of Molecular and Experimental Medicine, The Scripps Research InstituteLa Jolla, California, USA; 3 The Scripps Translational Science Institute, Scripps HealthLa Jolla, California, USA; 4 Department of Chemical Physiology, The Scripps Research InstituteLa Jolla, California, USA; 5 Molecular and Cellular Neuroscience, The Scripps Research InstituteLa Jolla, California, USA; 6 Division of Biology and Bioengineering, Broad Center, Howard Hughes Medical Institute, California Institute of Technology1200 East California Boulevard, Pasadena, CA, 91125, USA

**Keywords:** aging, dopamine, drugs, oxidative stress, pharmaceutical, serotonin

## Abstract

One goal of aging research is to find drugs that delay the onset of age-associated disease. Studies in invertebrates, particularly *Caenorhabditis elegans*, have uncovered numerous genes involved in aging, many conserved in mammals. However, which of these encode proteins suitable for drug targeting is unknown. To investigate this question, we screened a library of compounds with known mammalian pharmacology for compounds that increase *C. elegans* lifespan. We identified 60 compounds that increase longevity in *C. elegans*, 33 of which also increased resistance to oxidative stress. Many of these compounds are drugs approved for human use. Enhanced resistance to oxidative stress was associated primarily with compounds that target receptors for biogenic amines, such as dopamine or serotonin. A pharmacological network constructed with these data reveal that lifespan extension and increased stress resistance cluster together in a few pharmacological classes, most involved in intercellular signaling. These studies identify compounds that can now be explored for beneficial effects on aging in mammals, as well as tools that can be used to further investigate the mechanisms underlying aging in *C. elegans*.

## Introduction

Studies of the short-lived nematode, *Caenorhabditis elegans*, have uncovered numerous genes involved in aging, some of which cluster in specific biochemical pathways (Donmez & Guarente, 2010; Kenyon, 2010). It has become increasingly evident that at least some mechanisms that underlie aging in *C. elegans,* and fruit flies are evolutionarily conserved in mammals. For example, dietary restriction (DR) can increase lifespan in multiple organisms ranging from yeast to mammals and decreased signaling through the insulin/insulin-like growth factor (IGF) signaling pathway can increase lifespan not only in *C. elegans*, but also in mice (Holzenberger *et al*., 2003; Harrison *et al*., 2009).

Can interventions that increase longevity also delay the onset of age-associated disease? Several observations are indeed consistent with this idea. DR, reduced insulin/IGF signaling, and decreased TOR (target of rapamycin) signaling are all reported to delay the onset, or improve the outcome, of certain age-related diseases, such as cancer or neurodegenerative disease in *C. elegans* and mouse models of these diseases (Hursting *et al*., 1994; Pinkston *et al*., 2006; Raffaghello *et al*., 2008; Cohen *et al*., 2009; Rangaraju *et al*., 2009; Johnson *et al*., 2013).

What strategies are most likely to lead to drugs for combating the deleterious effects of aging in humans? The ability of small molecules to extend lifespan has now been amply demonstrated in invertebrates (Kang *et al*., 2002; Evason *et al*., 2005; Wilson *et al*., 2006; Petrascheck *et al*., 2007; McColl *et al*., 2008; Srivastava *et al*., 2008; Pietsch *et al*., 2009; Onken & Driscoll, 2010; Alavez *et al*., 2011) and also confirmed in mice by the finding that lifespan can be increased by rapamycin, an immunosuppressant that blocks TOR activity (Harrison *et al*., 2009).

To identify additional drugs that would delay aging, but not have other undesirable effects, one could begin by using a ‘reverse pharmacology’ approach in which one would screen for compounds that target proteins implicated in aging and then test those compounds for effects on aging.

Alternatively, one could use a ‘forward pharmacology’ approach in which compounds would be directly screened *in vivo* for their ability to delay aging or age-associated phenotypes. However, aging and lifespan are ‘whole organism’ phenotypes that would make *in vivo* screens in mammals time-consuming and prohibitively expensive.

Given the apparent evolutionary conservation of aging mechanisms, we reasoned that it might be possible to circumvent these problems by first screening for compounds that increase the lifespan of a short-lived invertebrate and then testing the identified compounds for beneficial effects in mammals. By screening compounds with known mammalian targets, many with established safety profiles, for those that extend the lifespan of *C. elegans*, it may be possible to hasten the identification of compounds with similar effects in mammals. In this context, rapamycin provides a proof of principle as it extends lifespan in invertebrates as well as in mammals (Harrison *et al*., 2009; Robida-Stubbs *et al*., 2012).

As a first step in this direction, we screened for compounds that increase *C. elegans* longevity using a library of 1280 compounds with known or suspected mammalian targets, many approved for use as drugs in humans. These studies identified 60 compounds that increased *C. elegans* lifespan. These compounds act on a variety of mammalian proteins, suggesting the potential involvement of homologous nematode proteins in aging. Interestingly, similar to some genetic alterations that increase *C. elegans* longevity, 33 of the compounds also increased the animal’s resistance to oxidative stress.

## Results

### A large-scale screen for compounds that increase *C. elegans* lifespan

**Figure 1:**
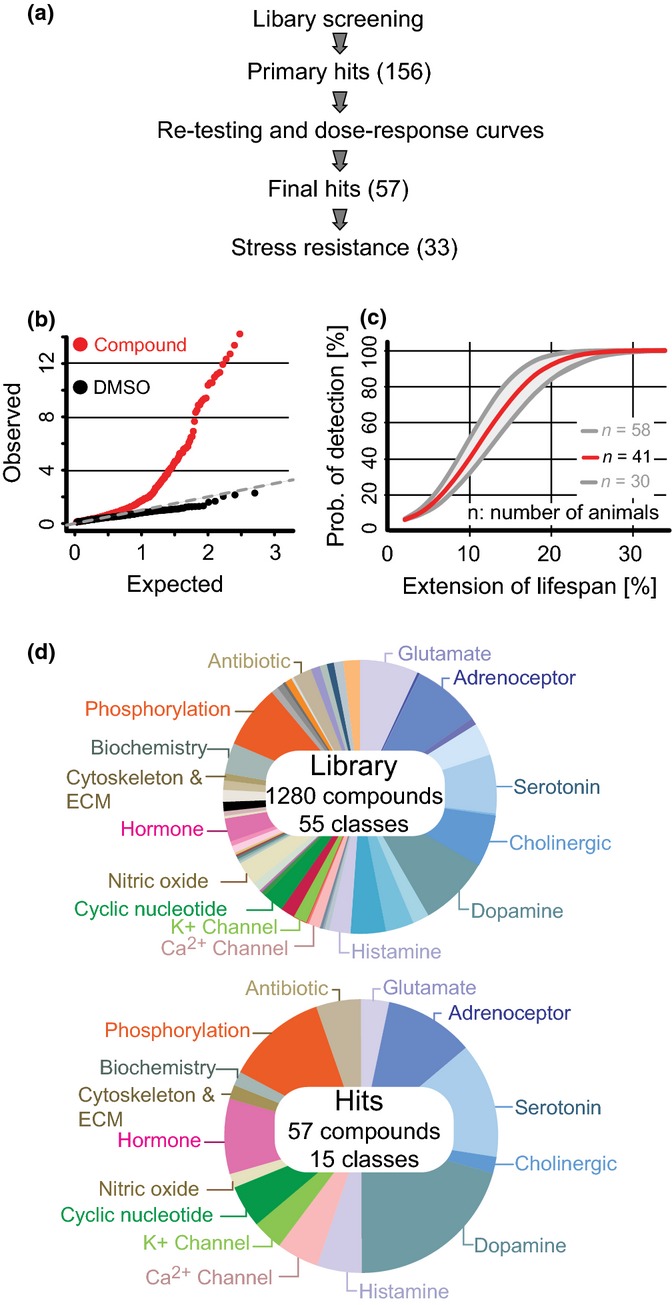
Overview of screening strategy and results. (a) Schematic shows screening strategy and results, with numbers of compounds in parentheses. (b) Q-Q plot showing lifespan *P* value-distribution for animals treated with DMSO (black) or compounds (red). Dashed line shows expected *P* value distribution due to chance. (c) *In silico* modeling of control data shows the probability of detecting a given increase in lifespan using the numbers of animals employed in the screen (n) (average, 41 (red line); range in >90% of experiments, 30–58). (d) Pie charts show the fraction of compounds belonging to different pharmacological classes in the Pharmacologically Active Compounds (LOPAC) library (Library) and among compounds that increased lifespan (Hits).

To search for compounds that increase lifespan when given to adult *C. elegans*, we screened a commercial collection of pharmacological agents with known or suspected targets in humans. This collection, called Library of Pharmacologically Active Compounds (LOPAC), contains 1280 different compounds that are grouped into pharmacological classes according to their mammalian targets (Fig. 1d, Experimental Procedures). Many of the compounds in the LOPAC library are in current use as pharmaceutical agents in humans.

To screen the LOPAC library for compounds that increase *C. elegans* lifespan, we used methods similar to those we employed in a previous screen of 88 000 small molecules of undefined function (Petrascheck *et al*., 2007, 2009; Solis & Petrascheck, 2011). Animals were grown in liquid medium at 20 **°**C in 96-well plates with 5–15 animals per well. Beginning at day 1 of adulthood, animals in each well were exposed continuously to a single compound or to the vehicle control (0.33% DMSO). Each compound was tested at a concentration of 33 μm on an average population of 41 ± 7 animals. The fraction of live animals per well was monitored until 98.1% was no longer alive (59 783 of 60 921 animals). The mean lifespan of animals treated with the vehicle control was 21.1 ± 0.7 days, well in agreement with the literature (Kenyon, 2010).

In the 1280 compounds screened, we obtained 156 primary hits using both the Cox-proportional hazard model and the Mantel–Haenzel version of the log-rank test (Fig 1a, Table S1). We leveraged a two stage-procedure, whereby we intentionally chose ‘soft’ statistical criteria for primary hits with a false discovery rate (FDR) of 0.66 (Storey & Tibshirani, 2003). This was performed to maximize the identification of true positive hits, but at the expense of including a large number of false positives, which could later be removed in a secondary screen.

Standard quality control measures for high-throughput assays, such as the Z’ factor (Zhang *et al*., 1999), are inadequate for lifespan screens because survival data are not normally distributed (Fig. S5–S7 Supplementary Methods). Instead, we assessed the quality of the screen by three different methods. First, we estimated the uniformity of the DMSO controls and the possible number of hits by generating a Q-Q plot that graphs expected versus observed *P* values (Fig. 1b). In Q-Q plots, *P* values due to chance will follow a 45º line (dashed line) as was observed for the DMSO-treated control populations (*n* = 250 control populations). This confirmed the uniformity of the screening conditions. In contrast, the *P* values for compound-treated populations very strongly deviated from the 45º line suggesting that a large number of compounds affected lifespan.

Second, we estimated the ability of the screen to detect any given percent increase in lifespan. This was performed by generating a parametric survival time model based on the Gompertz equation using the DMSO-treated control population as input data. This model allowed us to simulate the screen *in silico* (Johnson, 1990) (Fig. S1c). As a test, we conducted a reference screen in which we evaluated 122 populations of animals treated with vehicle alone and six populations treated with mianserin, a compound that extends lifespan by 31% (Petrascheck *et al*., 2007, 2009; Yu *et al*., 2010). All six mianserin-treated populations were identified as hits, as two populations were treated with vehicle alone (false positives). This empirical detection rate was consistent with the survival time model-derived detection rate of 99% for mianserin (Fig. 1c).

Third, we examined library compounds previously reported to extend nematode lifespan at the concentration and temperature used in our screen. We determined that the hits obtained in the primary screen included all four such compounds: mianserin, cyproheptadine, methiothepin, and pregnenolone (Broue *et al*., 2007; Petrascheck *et al*., 2007). A fifth compound, doxycycline, reported to extend lifespan subsequent to these analyses, also belonged to the selected hits (Houtkooper *et al*., 2013). In addition, another compound that was represented twice in the library was found to extend lifespan in both instances. Taken together, these tests confirmed the high quality of the screening data.

We next conducted secondary screen on 153 of the 156 primary hits, excluding three compounds previously found to increase lifespan, but keeping a fourth (mianserin) as a positive control. Due to the high FDR, we used in the primary screen (0.66), we expected roughly 100 false positives among the primary hits. We first retested the 59 weakest primary hits at 33 μm, and the concentration used in the primary screen. Of these weaker primary hits, 54 were false positives, with only five compounds showing a positive effect on lifespan. We then tested these five compounds and the remaining 94 stronger primary hits at five different concentrations ranging from 3 to 176 μm, using 30–99 animals for each concentration (Fig. S3). A compound was considered a secondary hit if it produced a significant increase in lifespan at two consecutive concentrations. Exceptions were made for two compounds (nicardipine and BRL15572) that extended lifespan at only a single concentration, but did so with a *P* value of <10^−5^. Compounds identified as secondary hits were each tested on a minimum of 128 animals, with an average of 245 animals tested per compound (Table S2).

The LOPAC library contains 28 antibiotics, three of which increased *C. elegans* lifespan (by 16–29%; Table 1). Although one of these three tetracycline antibiotics, minocycline, has annotated mammalian targets, this effect could be caused by killing or by preventing growth of the bacteria used for food, as feeding *C. elegans* with dead, or nonproliferating bacteria can increase lifespan (Gems & Riddle, 2000; Garigan *et al*., 2002; Cabreiro *et al*., 2013). To test whether these three antibiotics are the only lifespan-extending compounds with antibiotic activity, we measured the effect of each compound on the growth of the bacterial strain used in the screen (OP50) at a concentration 1.5-fold higher than the optimal concentration used in the lifespan assay. Bacterial growth was inhibited by all three antibiotics, as well as by the dopamine receptor agonist N-(2-[4-(4-Chlorophenyl) -b -piperazin- 1-yl]ethyl)-3-methoxybenzamide and the nitric oxide donor 4-Phenyl-3-furoxancarbonitrile. Lifespan extension by these five compounds could therefore be due to their effects on the feeding bacteria. The other 52 compounds that increased lifespan had no detectable effect on bacterial growth (Fig. S4). We decided to include all compounds in further analysis, however, because doxycycline was shown recently to increase lifespan in animals fed with tetracycline-resistant bacteria and minocycline was found to increase lifespan in *Drosophila melanogaster* (Oxenkrug *et al*., 2012; Houtkooper *et al*., 2013), suggesting antibiotic-independent mechanisms for both compounds.

**Table 1:** Name, pharmacology and effects of the 57 hit compounds

Class	Compound/drug	Target^a^	Action^b^	Lifespan increase^c^ (%)	OSR change^d^ (%)
Antibiotic	Demeclocycline hydrochloride*	Bacterial 30S subunit	−	16	37***
	Doxycycline hydrochloride*	Bacterial 30S subunit	−	18	51***
	Minocycline hydrochloride*	Bacterial 30S subunit (mammals: MMP9, VEGF, ALOX5, Cytochr. C, IL1B, CASP-1, CASP-3)	−	29	−39***
Biochemistry	3,4-Dichloroisocoumarin	Serine proteases	−	13	30***
Biogenic amine/adrenoceptor	Amoxapine*	SLC6A2 (HTR 2A, 2C, 6, 7; DRD2; SLC6A4)	−	33	97***
	Doxazosin mesylate*	ADRA1A (ADRA 1B, 1D, 1C)	−	15	4
	Guanabenz acetate*	ADRA2A	**+**	12	29***
	Guanfacine hydrochloride*	ADRA2A	**+**	15	27
	Naftopidil dihydrochloride	ADRA1A	−	14	31***
	Nortriptyline hydrochloride*	SLC6A2 (ALB; SLC6 A2, A4; HTR 2A, 2C, 6; ADRA1A; CHRM1, M2, M3, M4, M5; HRH1)	−	21	63***
Biogenic amine/dopamine	(±)-Octoclothepin maleate	DRD2 (DRD1, 3, 4; HTR 2A, 6, 7)	−	38	108***
	BTCP hydrochloride	SLC6A3	−	14	23
	Chlorprothixene hydrochloride*	DRD2 (HTR 2A, 2B, 2C, 6, 7; DRD3, 4; CHRM1, M2, M3, M4, M5; HRH1)	−	33	91***
	cis-(Z)-Flupenthixol dihydrochloride*	DRD1 (DRD2, D4, D5; ABCB1; HTR 2A, 2C; ADRA1A, HRH1)	−	30	92***
	Cortexolone maleate	DRD2 (precurser of cortisol synthesis)	−	11	−17
	Dihydroergocristine methanesulfonate	DRD2 (ADRA1A, HTR-receptors)	**±**	34	74***
	Loxapine succinate*	DRD2 (DRD1, D3, D4, D5; HTR 2A, 2C, 6, 7; ADRA1A, 1B, 2A, 2B, 2C; CHRM1, M3; HRH1)	−	43	99***
	Methylergonovine maleate*	DRD1 (HTR 1E, 1F, 2A, 2B, 2C, 7)	−	28	106***
	N-(2-[4-(4-Chlorophenyl)piperazin-1-yl]ethyl)-3-methoxybenzamide	DRD4	**+**	35	101***
	Pergolide methanesulfonate*	DRD1, DRD2 (DRD3, D4, D5; HTR 1A, 1D, 2A, 2B, 6, 7; ADRA 2A, 2B, 2C; HRH1)	**+**	37	97***
	Propionylpromazine hydrochloride	DRD2	−	20	78***
	Thioridazine hydrochloride*	DRD1, DRD2 (DRD3, D4; HTR 1A, 1B, 2A, 2C, 6, 7; ADRA 1A, 1B, 2A, 2C; CHRM1, M2, M3, M4, M5; HRH1)	−	31	28***
Biogenic amine/histamine	Loratadine*	HRH1	−	18	41***
	Oxatomide	HRH1	−	25	71***
	Promethazine hydrochloride*	HRH1 (CHRM1, M5; DRD2; HTR2A, ADRA1A)	−	32	81***
Biogenic amine/serotonin	PAPP/LY-165,163	HTR1A (HTR1D, DRD2)	**±**	33	83***
	Amperozide hydrochloride	HTR2A (HTR6, ADRA1A, DRD2)	−	38	60***
	BRL 15572	HTR1D (HTR 1A, 1B, 2A, 2B)	−	10	15
	Dihydroergotamine methanesulfonate*	HTR1D (ADR2A, HTR2B)	**+**	24	69***
	Ketanserin tartrate	HTR2A (HTR2C, ADRA1A)	−	13	40***
	LY-367,265	HTR2A (SLC6A4)	−	34	83***
	Metergoline	HTR2A (HTR1B, 1A, 2C, 6, 7)	−	23	65***
	Mianserin hydrochloride*	HTR2A (HTR 1A, 1D, 2A, 2B, 2C, 3, 6, 7; ADRA 2A, 2B, 2C; HRH1; OPRK1)	−	32	77***
Ca2+ Channel	Cinnarizine*	CACNA1A (HRH1, DRD2, HRH4)	−	15	8
	Nicardipine hydrochloride*	CACNA1C (ABCB1, ABCG2)	−	23	−16
	Nitrendipine*	CACNG1 (ABCG2)	−	25	39***
Cholinergic	Hexahydro-sila-difenidol hydrochloride	CHRM3 (CHRM1, M2)	−	15	22
Cyclic Nucleotide	BRL 50481	PDE7	−	18	1
	Trequinsin hydrochloride	PDE3	−	27	64***
	Vinpocetine	PDEI	−	15	−3
Cytoskeleton and ECM	Vincristine sulfate*	TUBB2A	−	12	13
Glutamate	AMN082	Grm7	**+**	8	18
	Eliprodil	NMDA	−	16	51***
Hormone	(R,R)-cis-Diethyl tetrahydro-2,8-chrysenediol	ESR2, ESR1	**±**	7	1
	Beta-Estradiol*	ESR1, ESR2, SHBG, NR1I2	**+**	7	12
	Cyproterone acetate*	AR	−	23	−7
	Danazol*	ESR1 (GNRHR, R2; SHBG, CCL2)	**+**	13	−2
K+ Channel	Psora-4	Kv1.3	−	42	−22
	Quinidine sulfate*	KCNK1 (KCNH2, KCNK6, SCN5A, CHRM2)	−	12	15
Nitric Oxide	4-Phenyl-3-furoxancarbonitrile	Nitric oxide donor	N/A	30	4
Phosphorylation	7-Cyclopentyl-5-(4-phenoxy)phenyl-7H-pyrrolo[2,3-d]pyrimidin-4-ylamine	lck	−	11	8
	Cyclosporin A*	CAML (PPIA, ABCB1, PPP3R2)	−	18	43***
	DAPH	EGFR	−	15	43***
	Kenpaullone	CDK1 (CDK2, CDK5, GSK3)	−	27	−31***
	LFM-A13	BTK	−	27	−60***
	SU 4312	VEGEFR, PDGFR	−	5	27***
	Tyrphostin AG 1478	EGFR	−	11	21

* Compounds/Drugs approved for human use.

*** *P <* 0.005 for the observed change in stress resistance.

a Target information was obtained using the LOPAC annotation from Sigma and information from DrugBank and the PDSP database; Sigma annotations were used for primary target classifications.

b Describes whether the compound has an activating (+) or inhibiting (−) effect on the target. Some compounds show different actions on different targets.

c Describes% increase in lifespan relative to DMSO-treated animals; average of three to six independent experiments using the optimal concentration of compound.

d Describes% change in survival under conditions of oxidative stress relative to DMSO-treated animals, (*C. elegans*); mean of four experiments shown.

**Figure 2:**
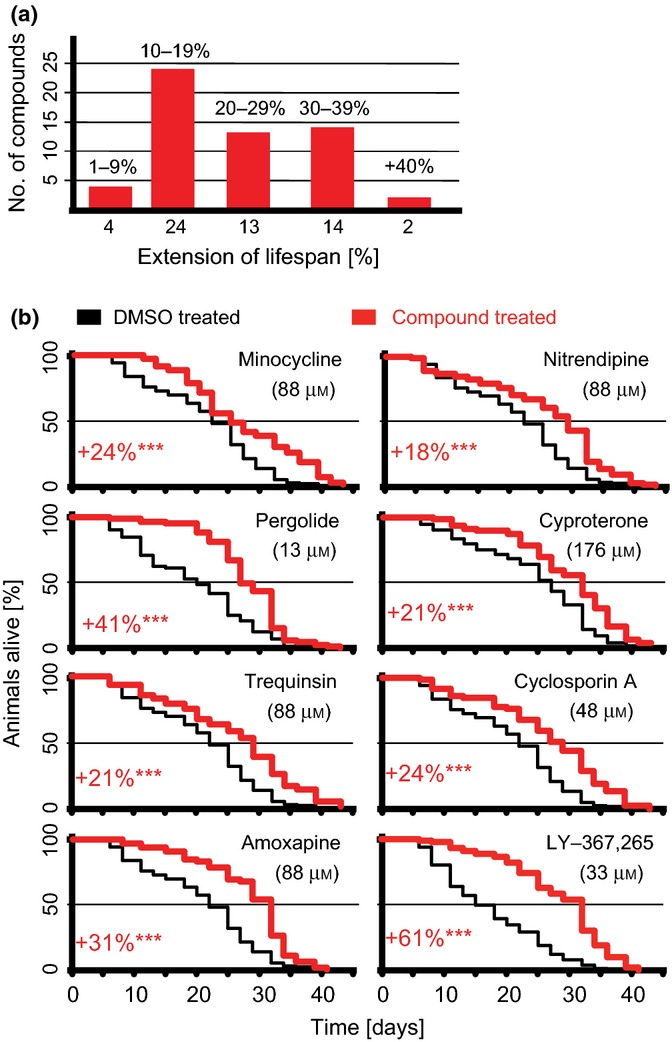
Numerous compounds increase *Caenorhabditis elegans* lifespan. (a) Bars show the number of compounds that increased lifespan by different percentages. The range of percent lifespan extension is indicated at the top of each bar and the number of compounds at the bottom. (b) Survival curves from representative experiments show the percent of animals alive on different days [red, compound-treated; black, vehicle-treated (DMSO)]. Percent lifespan increase is shown for each compound (red) with asterisks indicating significant increases (***, *P* < 0.0001). For number of animals used and exact *P* values see Table S2 (Supporting information).

These experiments identified 57 compounds (including mianserin) as secondary hits that produced a statistically significant increase in *C. elegans* lifespan. Five of these compounds could increase lifespan via their direct effects on nematodes or indirect effects resulting from the inhibition of growth of the feeding bacteria. Four compounds extended lifespan by an average of 1–9%, 24 by 10–19%, 13 by 20–29%, 14 by 30–39% and 2 by 40% or more (Fig. 2). Of the 57 compounds, nearly half (27/57) have been approved for use as pharmaceutical drugs in humans (Table 1, Fig. S2).

### Lifespan extending compounds cluster in certain pharmacological classes

The 1280 compounds in the LOPAC library are grouped into 55 different pharmacological classes based on their protein targets in mammals (humans/mice). The 57 compounds that increased *C. elegans* lifespan belong to 15 of those classes (Fig. 1d). The presence of numerous proteins with related sequences and functions in *C. elegans* and mammals suggests that many of the identified compounds might interact with homologous proteins in humans and nematodes. Consistent with this idea, several compounds that interact with human proteins have previously been shown to interact with a homologous *C. elegans* protein with a related function (Kwok *et al*., 2006; Petrascheck *et al*., 2007).

Notably, a large percentage (51/57, 89%) of the compounds that increased *C. elegans* lifespan in these studies target mammalian proteins involved in intercellular signaling. Four library classes with compounds that increased nematode longevity contain drugs that target mammalian biogenic amine receptors. These receptors are G protein-coupled receptors (GPCRs) that recognize adrenaline/noradrenaline, dopamine, histamine, or serotonin. The biogenic amine receptors are closely related to one another and many of the compounds that act on one type also have activity on another (Table 1). Many of the compounds that act on these receptors are also related in structure (Fig. S1). Most of the compounds from these classes that increased *C. elegans* lifespan are antagonists for their mammalian target receptors. The maximum increase in lifespan seen with these compounds was 43% (Table 1, Table S2). *C. elegans* has receptors homologous to mammalian biogenic amine receptors that recognize serotonin, dopamine, tyramine, or octopamine, though histamine receptors have not been identified thus far. Moreover, previous studies indicate that mianserin increases nematode lifespan by inhibiting two different nematode biogenic amine receptors and that animals mutant for a different nematode receptor of this class show increased longevity (Murakami & Murakami, 2007; Petrascheck *et al*., 2007).

Three other compound classes that increased nematode lifespan have functions related to those of the biogenic amine receptors. Compounds identified in two of these classes, the cholinergic and glutamate classes, act on GPCRs that are activated by acetylcholine (muscarinic acetylcholine receptors) or glutamate (metabotropic glutamate receptor, Grm7). Compounds acting on these receptors increased *C. elegans* lifespan by 15% and 8%, respectively. A third class related to the biogenic amine class is the ‘cyclic nucleotide’ class. The three members of this class that increased *C. elegans* lifespan (by 15–27%) are all inhibitors of human cyclic nucleotide phosphodiesterases (PDEs). PDEs are linked to GPCR signaling in that they degrade cAMP or cGMP generated by signaling through some GPCRs.

Two additional classes of compounds that target proteins involved in transmembrane signaling are those that act on ion channels. In addition to one member of the glutamate class that acts on a glutamate-gated ion channel [the NMDA receptor (+16% lifespan increase)], targets of these compounds include calcium (25% maximum lifespan increase) and potassium channels (42% maximum lifespan increase).

Other compounds that increased *C. elegans* lifespan include those that target mammalian serine proteases (+13%), tubulin (+12%), nuclear hormone receptors (+7–23%), a nitric oxide donor (+30%), and cytoplasmic protein kinases or receptor tyrosine kinases (+5–27%). Three tetracycline-type antibiotics also increased lifespan (+16–29%), but could do so indirectly by killing feeding bacteria, as noted above.

These results demonstrate that *C. elegans* lifespan can be extended by numerous compounds that target a large variety of different mammalian proteins. *C. elegans* has proteins homologous to many, and possibly most of the annotated targets of these compounds. While those homologs are the most likely targets of the identified compounds in the nematode, future studies will be needed to ascertain whether the homologs are involved in the observed effects of the compounds on nematode lifespan. As many of the possible *C. elegans* targets have not previously been linked to aging, such studies could potentially provide additional information as to the mechanisms underlying aging in the animal.

Many of the compounds identified in these studies act on multiple mammalian proteins and could actually belong to several pharmacological classes. To gain further insight into interrelationships among the compounds found to increase lifespan, we generated a pharmacological network (Fig. 5) (Smoot *et al*., 2011). The network incorporates compound-target interaction data based on LOPAC library annotations, DrugBank, and the PDSP-binding database (Table 1) (Roth *et al*., 2000; Knox *et al*., 2011). This approach generated a network consisting of 139 nodes (57 compounds and 82 protein targets) with three main network clusters centered around hormone-signaling, tetracycline antibiotics and biogenic amine signaling.

The pharmacological network clusters all the biogenic amine targeting compounds into one network component through common receptor targets. The most highly targeted receptors are the dopamine receptor DRD2 and the serotonin receptor HTR2A. It further connects calcium channel blockers to the biogenic amine network through binding of HRH1 and DRD2. Compounds targeting hormone signaling form a cluster that is distinct from biogenic amine signaling and that contains three of the four compounds that affect hormone signaling, all connected by common targets.

Even though no structural information went into the generation of this network, it clusters many structurally similar compounds through their common targets. Alkaloids like methylergonovine and metergoline, belong to different pharmacological classes, but are structurally related (Fig. S1) and are connected through the binding to HTR2A, HTR2C, and HTR7 serotonin receptors (Fig. 5). Similarly, the calcium channel blocker cinnarizine is connected to the antihistamine oxatomide through binding to HRH1. Despite their different classification, these two compounds share structural similarities. In addition, all components in the hormone signaling network are structurally related. Over all, structurally similar compounds tend to cluster together in the pharmacological network.

### Lifespan-extending compounds that protect *C. elegans* from oxidative stress

Decreases in the ability to respond to different forms of stress have been proposed to play an important role in aging and susceptibility to age-associated diseases. Consistent with this idea, long-lived *C. elegans* mutants in the insulin/IGF-signaling pathway show increased resistance to oxidative stress as do long-lived nematodes subjected to RNAi targeting the electron transport chain (Honda & Honda, 1999; Lee *et al*., 2006), though other interventions that increase longevity do not affect stress resistance. While increased oxidative stress resistance alone is unlikely to be sufficient to increase longevity, it may be associated with an array of changes that together increase lifespan in certain settings (Gems & Doonan, 2009; Van Raamsdonk & Hekimi, 2009; Yang & Hekimi, 2010; Shore *et al*., 2012).

To examine whether any of the 57 compounds that increased lifespan can influence the response of *C. elegans* to oxidative stress, we tested the effects of these compounds on animals exposed to paraquat, a generator of radical oxygen species (ROS) (Fukushima *et al*., 2002). Starting from day 1 of adulthood, animals were treated with a single compound for five days and then paraquat was added to a final concentration of 100 mm. The fraction of live animals per well was measured 24 h later.

**Figure 3:**
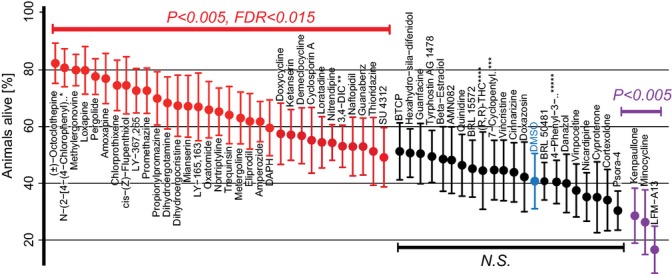
Many compounds protect *Caenorhabditis elegans* from oxidative stress. Animals were exposed to individual compounds or the vehicle control (DMSO) (blue) for 5 days and then to paraquat for 24 h before survival was determined. Of 57 compounds that increased lifespan, 33 (red) caused a significant increase in the percentage of live animals compared to the control and 3 (purple) caused a significant decrease whereas the others (black) had no significant effect. *P* value and false discovery rate are indicated. Shown are average values from four experiments. For values and cohort sizes see Table 3 (Supporting information). Error bars indicate SEM. Asterisks indicate compounds whose names are abbreviated: N−(2−[4−(4−Chlorophenyl)*, N−(2−[4−(4−Chlorophenyl)piperazin−1−yl]ethyl)−3−methoxybenzamide 3,4−DIC **, 3,4−Dichloroisocoumarin 7−Cyclopentyl***, 7-Cyclopentyl-5-(4-phenoxy)phenyl-7H-pyrrolo[2,3-d]pyrimidin-4-ylamine, (R,R)-THC****, (R,R)−cis−Diethyltetrahydro−2,8−chrysenediol, 4−Phenyl−3-*****, 4−Phenyl−3−furoxancarbonitrile.

Of the 57 compounds tested, 33 (57.9%; *P* < 0.005, FDR 0.015) caused a significant increase in the survival of animals exposed to paraquat, 21 had no effect, and three reduced resistance to oxidative stress. Of the control animals that received vehicle alone (0.33% DMSO), 39.8% were alive following paraquat treatment. In contrast, in the presence of the 33 compounds with a significant effect, the percentage of live animals ranged from 50.6% to 83%, corresponding to increases in stress resistance of 27% to 108%, respectively (Fig. 3).

The 33 compounds that increased *C. elegans* resistance to oxidative stress belong to nine of the 15 pharmacological classes that contained lifespan-extending compounds. Compounds that increased stress resistance included those that target the following mammalian proteins: all four classes of biogenic amine receptors, a phosphodiesterase, the NMDA ionotropic glutamate receptor, a protease inhibitor, calcium channel blockers, antibiotics, and a receptor tyrosine kinase. The greatest effects were seen with three compounds that target mammalian dopamine receptors (80–83% of animals alive). The lowest protection was seen with a compound (SU4312) that acts on the mammalian VEGF receptor (50.6% alive). By choosing an FDR of 0.015, we expect less than one false positive among those 33 compounds. Twenty-one compounds did not increase oxidative stress resistance, however, and three compounds (LFM-A13, kenpaullone and minocycline) significantly decreased oxidative stress resistance. Thus, like some other interventions that increase lifespan, some compounds that increase lifespan also increase resistance to oxidative stress, whereas others do not.

**Figure 4:**
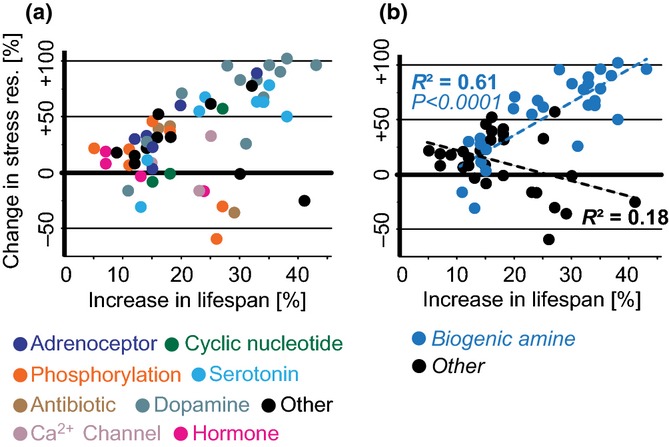
Correlation between effects of compounds on lifespan and stress resistance. (a) A graph compares the effects of different compounds on *C. elegans* lifespan versus resistance to oxidative stress induced by paraquat. Values indicate percent change in lifespan (*X*-axis) or survival (stress resistance) (*Y*-axis) relative to controls. Each dot represents a single compound whose pharmacological class is indicated by a color, as noted below. (b) A graph like that shown in a) in which all the compounds have been grouped as either ‘biogenic amine’ targeting or ‘other’. Correlations and *P* value are indicated.

Analyzing the relationship between lifespan and stress resistance increases by pharmacological class, we found that the ability to increase lifespan and oxidative stress resistance correlated strongly for compounds that target biogenic amine receptors (*R*^2^ = 0.61, Fig. 4), but not for compounds in other pharmacological classes (*R*^2^ = 0.18, Fig. 4b). These findings suggest that some compounds increase lifespan by mechanisms independent of those involved in oxidative stress resistance, but that there could be a mechanistic link between oxidative stress resistance and lifespan extension by compounds that target mammalian biogenic amine receptors.

**Figure 5:**
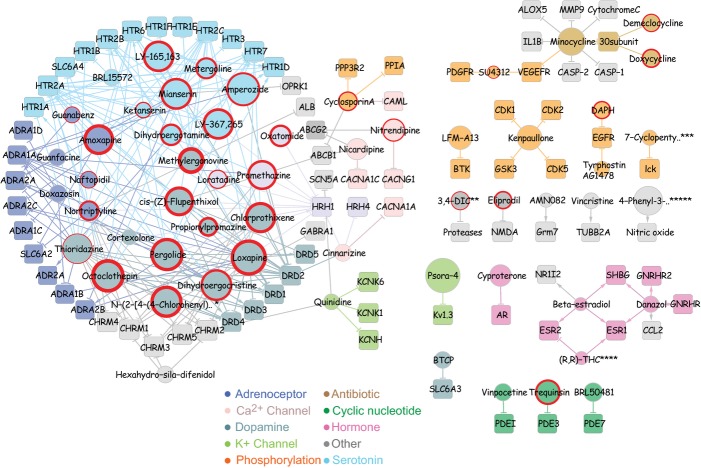
Pharmacological network for lifespan extension. Shown is a network generated by connecting compounds (circles) and their respective protein targets (squares). Arrows indicate agonist action, and T’s indicate antagonist action. For compounds (circles), the node size is proportional to the compound’s effect on lifespan whereas the size of protein nodes is fixed. Induction of stress resistance is indicated by a red ring around compounds, with the thickness of the ring proportional to the effect magnitude. Nodes (compounds and targets) and connections between nodes are colored according to pharmacology, as indicated in the key below. See the Fig. 3 legend for names of compounds that are abbreviated.

The same correlation is also evident in the pharmacological network (Fig. 5). The ability to induce resistance to oxidative stress is clustered around biogenic amine receptors, with 24 of 29 compounds that target these receptors increasing resistance to oxidative stress. Furthermore, 19 of 20 compounds that increase stress resistance by at least 60% are centered in the biogenic amine receptor network. In contrast, none of the compounds that target hormone signaling affected oxidative stress resistance.

## Discussion

One long-term aim of aging research is to find drugs that would delay the onset of age-associated disease in humans. Drug discovery and development are long and costly processes. However, it is possible that drugs previously developed for other purposes could prove beneficial for aging humans without requiring the years of medicinal chemistry and pharmacological safety profiling needed for the development of a new drug. *C. elegans* has homologs of many human proteins and a few compounds that target human proteins have been found to not only interact with their nematode counterparts, but also increase *C. elegans* lifespan, and in one case, the lifespan of both *C. elegans* and mice (Harrison *et al*., 2009; Robida-Stubbs *et al*., 2012). Here, we asked whether it would be possible to identify additional compounds that increase *C. elegans* longevity by conducting a large-scale screen of compounds with known pharmacological targets in mammals. In a screen of 1280 such compounds, we identified 57 compounds that increased *C. elegans* lifespan of which 27 are approved for human use. These studies provide candidate compounds to explore for helpful effects on aging in mammals as well as tools to further investigate the mechanisms underlying aging in *C. elegans*.

### Compounds that increase lifespan in *C. elegans*

These studies revealed that compounds that target a variety of mammalian proteins can increase *C. elegans* lifespan. They newly identified 56 compounds that increased *C. elegans* longevity by 5–43% as well as another four compounds previously shown to increase nematode lifespan (Broue *et al*., 2007; Petrascheck *et al*., 2007; Houtkooper *et al*., 2013). In the compound library screened, compounds are divided into 55 pharmacological classes according to the functions of their target proteins in humans/mice. Compounds that increased *C. elegans* lifespan belonged to 15 of those classes.

In these studies, 60/1280 screened compounds increased *C. elegans* lifespan, a hit rate of 4.7%. In contrast, when we previously screened 88 000 compounds of undefined function, only 0.13% (115/88 000) produced a statistically significant increase in *C. elegans* longevity. One possible explanation for this difference is that the 1280 compound LOPAC library comprises compounds with established biological activity whereas this is not the case for the 88 000 compound library. Consistent with this idea, examination of data from a different screen of *C. elegans* with the LOPAC library also suggests a high hit rate (>3%) (Kwok *et al*., 2006). It should also be noted that the LOPAC library contains multiple compounds that target the same protein or related proteins, another factor likely to contribute to a high hit rate. Indeed, 29/57 LOPAC compounds that increased nematode lifespan in the present studies act on interrelated biogenic amine receptors in mammals and may similarly act on the same or interrelated proteins in *C. elegans*.

The nematode targets of the compounds that increased *C. elegans* lifespan in these studies are not yet known. However, the presence of numerous homologous proteins with similar functions in nematodes and humans suggests that the compounds may well interact with related proteins in the two species. Compounds previously shown to interact with related proteins in nematodes and mammals include several that target serotonin receptors and a calcium channel blocker (Kwok *et al*., 2006; Petrascheck *et al*., 2007).

The majority of compounds that increased *C. elegans* lifespan target mammalian proteins involved in intercellular signaling in mammals (89%). Many of these compounds [29/57 (50.9%)] act on mammalian G protein-coupled receptors for biogenic amines (serotonin, dopamine, adrenaline/noradrenaline, histamine). Most, though not all, are receptor antagonists. The large number of identified compounds that act on these receptors may reflect their relatively high abundance in the library that was screened. *C. elegans* has GPCRs structurally related to those in mammals that recognize the same (serotonin, dopamine) or related (octopamine, tyramine) biogenic amines. Several human serotonin receptor antagonists were previously found to increase *C. elegans* longevity by inhibiting two homologous receptors, one for serotonin and the other for octopamine, and animals mutant for a different serotonin receptor also showed increased longevity. Those receptors are potential targets for some of the compounds identified here that target mammalian biogenic amine receptors, many of which act on multiple receptors of this class in mammals (Murakami & Murakami, 2007; Petrascheck *et al*., 2007).

The other compounds that increased *C. elegans* lifespan have a variety of different mammalian targets. These include several other types of GPCRs, phosphodiesterases that function downstream of GPCRs, calcium, and potassium channels, an ionotropic glutamate receptor, growth factor receptors, protein kinases, proteases, and nuclear hormone receptors. Interestingly, one compound that increased nematode lifespan (cyclosporin A) binds cyclophilin, causing inhibition of calcineurin, a protein whose *C. elegan’s* homolog (CNB-1) is involved in aging (Dong *et al*., 2007). Elucidation of the targets of the identified compounds in *C. elegans* may provide added information about aging mechanisms in the animal and contribute to a further understanding of those mechanisms.

### Compounds that increase stress resistance in *C. elegans*

These studies show that some compounds that increase *C. elegans* lifespan also increase the animal’s resistance to oxidative stress, one stressor proposed to play a role in aging (Harman, 1956). Decreased insulin/IGF signaling and some DR regimens also increase both lifespan and oxidative stress resistance in *C. elegans* (Honda & Honda, 1999; Houthoofd *et al*., 2002; Lee *et al*., 2006). While increased oxidative stress resistance alone is unlikely to affect lifespan, it might be part of a constellation of alterations that together allow the animal to live longer by increasing its ability to repair or prevent damage caused by different types of stressors.

Of 57 compounds that increased *C. elegans* longevity in the present studies, 33 (57.9%) increased the survival of nematodes exposed to paraquat, a ROS generator. There was a strong correlation between the effects of compounds on lifespan and resistance to oxidative stress (Fig. 4b) for compounds that target biogenic amines, but not for compounds from other pharmacological classes. The pharmacological network makes evident that all but seven of the compounds that increased oxidative stress resistance in *C. elegans* (26/33) are part of the biogenic amine receptor network (Fig. 5). Mammalian targets of the other seven compounds that increased nematode oxidative stress resistance are phosphodiesterases, proteases, the NMDA type of glutamate receptor, and the EGF receptor.

These results are consistent with previous observations that some long-lived *C. elegans* mutants exhibit increased resistance to oxidative stress, whereas others do not (Van Raamsdonk & Hekimi, 2009). Many of the compounds that interact with biogenic amine receptors can bind to more than one mammalian receptor of this type (Table 1, Fig. 5). By analogy, if these compounds increase oxidative stress resistance in nematodes by acting on homologous receptors for biogenic amines, the observed effects could conceivably be mediated by only one or a few receptors of this type. One intriguing question for future studies will be whether compounds that increase stress resistance in nematodes have similar effects in mammals.

The information obtained in these studies sets the stage for future studies to investigate whether compounds that increase lifespan in the nematode may also have a beneficial effect on aging in mammals. Given the large number of compounds against biogenic amine receptors that increased nematode lifespan in the present studies, it is intriguing that an allelic variant of one such receptor, dopamine DRD4, is reported to be enriched in humans over the age of 90 (Grady *et al*., 2013).

## Experimental procedures

### LOPAC®1280 library

The library was purchased from Sigma-Aldrich (St. Louis, MO, USA). We made the following changes to the library annotation. Minocycline was reassigned from the ‘Cell Cycle’ class to the ‘Antibiotic’ class. All phosphodiesterase inhibitors were classified as belonging to the ‘Cyclic Nucleotide’ class, eliminating the ‘phosphodiesterase’ and ‘calcium signaling’ classes. Quinidine was reassigned from the ‘Na+ Channel’ class to the ‘K+ Channel’ class. All antihistamines were assigned to the ‘biogenic amine/histamine’ class. Compounds in the ‘lipid signaling’ class were placed in the ‘lipid’ class. The class ‘cytokine and growth factor’ and the class ‘inflammation’ were re-assigned to the ‘immune system’ class. Compounds belonging to the class ‘gene regulation’ were re-assigned to the ‘transcription’ class. These reassignments reduced the original 60 classes to our annotation of 55 classes.

### Lifespan assays

The screening procedure was carried out as previously described (Petrascheck *et al*., 2007). A detailed protocol is on JoVe (doi: 10.3791/2496). In short, 5–15, age-synchronized animals were cultured in S-complete media in wells of 96-well plates containing E. coli OP50 as feeding bacteria (~2 × 10^9^ bacteria mL^−1^). It should be noted that the effects of compounds can vary depending upon the assay conditions. For the screen, each compound was tested at a final concentration of 33 μm and 0.33%. The concentration used was based on compound and DMSO concentrations in the library and our finding that >0.5% DMSO can affect lifespan. Animals were exposed to the compounds continuously beginning on day 1 of adulthood. Controls contained 0.33% DMSO (vehicle) alone. Screens were conducted blind with the names of the compounds coded. Living animals were scored by eye. Scoring was based on movement induced by shaking and application of light to each well before scoring.

### *Caenorhabditis elegans* stress resistance assays

Animals were cultured in 96-well plates under conditions identical to those used in the lifespan assays. Compounds were added on day 1 of adulthood. Five days later (day 5 of adulthood) paraquat (Sigma-Aldrich) was added to a final concentration of 100 mm. Twenty-four hours after that, survival was assessed as outlined earlier. Survival was expressed as the percentage of surviving animals compared with the total starting population. *P* values were calculated based on contingency tables and chi-square analysis and combined with false discovery rates to account for inflated *P* values (Gribbon *et al*., 2005).

### Statistical analysis of the lifespan screen

The screen consisted of 1536 populations of animals comprised of four independent wells each (5–15 animals per well). The lifespan results were analyzed using two different approaches, which gave nearly identical outcomes. In the first approach, we used the Cox-proportional hazard model and calculated FDRs (false discovery rates) as described by Storey and Tibshirani (Storey & Tibshirani, 2003). In the second approach, we used the (Mantel–Haenszel) version of the log-rank test. See supplementary data for details. Generating a Q-Q plot graphing expected versus observed *P*-values for each of the four wells of each of the 250 control populations (DMSO only) showed that the conditions across the screen were uniform. To determine whether a given compound extended lifespan, *P*-values using either the Cox-proportional hazard model or the Mantel–Haenszel version of the log-rank test were calculated by comparing compound-treated populations to DMSO-treated control populations within the same set of plates. Library plates were tested in quadruplicate; each contained 80 wells that received an individual compound and eight wells that received only DMSO. Comparisons were calculated within the same quadruplicates to account for plate to plate variations. Standard deviations were calculated on the basis of populations consisting of four wells each.

### Statistical analysis of stress resistance in *C. elegans*

Stress resistance experiments for different compounds were conducted in parallel. Each compound was tested in four experiments using the concentration that gave the highest increase in lifespan (Tables S2 and S3). *P* values were calculated by comparisons across all four sets of experiments for the number of live and dead animals when animals were exposed to a compound versus control vehicle alone. FDR calculations were conducted as outlined in Storey and Tibshirani (Storey & Tibshirani, 2003)**.** The standard error of the mean, S.E.M., was calculated on the basis of the number of independent experiments.

### Parametric model

Parametric modeling was based on the Gompertz equation. For an exact description of the derivation of the model, see the Data S1 (Supporting information).

## Supporting Information

**Fig. S1** Lifespan-extending compounds can show structural similarity or have common substructures. **Fig. S2** Structures of lifespan-extending compounds and their effects on stress resistance. **Fig. S3** Dose response curves for lifespan-extending compounds. **Fig. S4** Effects of lifespan-extending compounds on bacterial growth. **Fig. S5** Parametric survival-time model based on the Gompertz equation. **Fig. S6** Density function *f*(t). **Fig. S7** Probability of detecting % increases in lifespan. **Table S1** Results of primary screen. **Table S2** Summary of *C. elegans* lifespan experiments with 57 hit compounds. **Table S3** Summary of stress resistance experiments in *C. elegans*. **Table S4** Screening data overview and total numbers. **Data S1** Generation of a parametric survival-time model.
