# Supplementary material for: A pharmacological network for lifespan extension in Caenorhabditis elegans
Source: Aging Cell. 2013 Nov 13;13(2):206–15. doi: 10.1111/acel.12163 (PMC3955372; doi:10.1111/acel.12163)
Supplement: Supplementary file 1 — Fig. S1 Lifespan-extending compounds can show structural similarity or have common substructures. Fig. S2 Structures of lifespan-extending compounds and their effects on stress resistance. Fig. S3 Dose response curves for lifespan-extending compounds. Fig. S4 Effects of lifespan-extending compounds on bacterial growth. Fig. S5 Parametric survival-time model based on the Gompertz equation. Fig. S6 Density function f(t). Fig. S7 Probability of detecting % increases in lifespan. Table S1 Results of primary screen. Table S2 Summary of C. elegans lifespan experiments with 57 hit compounds. Table S3 Summary of stress resistance experiments in C. elegans. Table S4 Screening data overview and total numbers. Data S1 Generation of a parametric survival-time model. [file acel0013-0206-sd1.pdf]

Supplementary Fig. 1
Lifespan-extending compounds can show structural similarity or have common substructures.

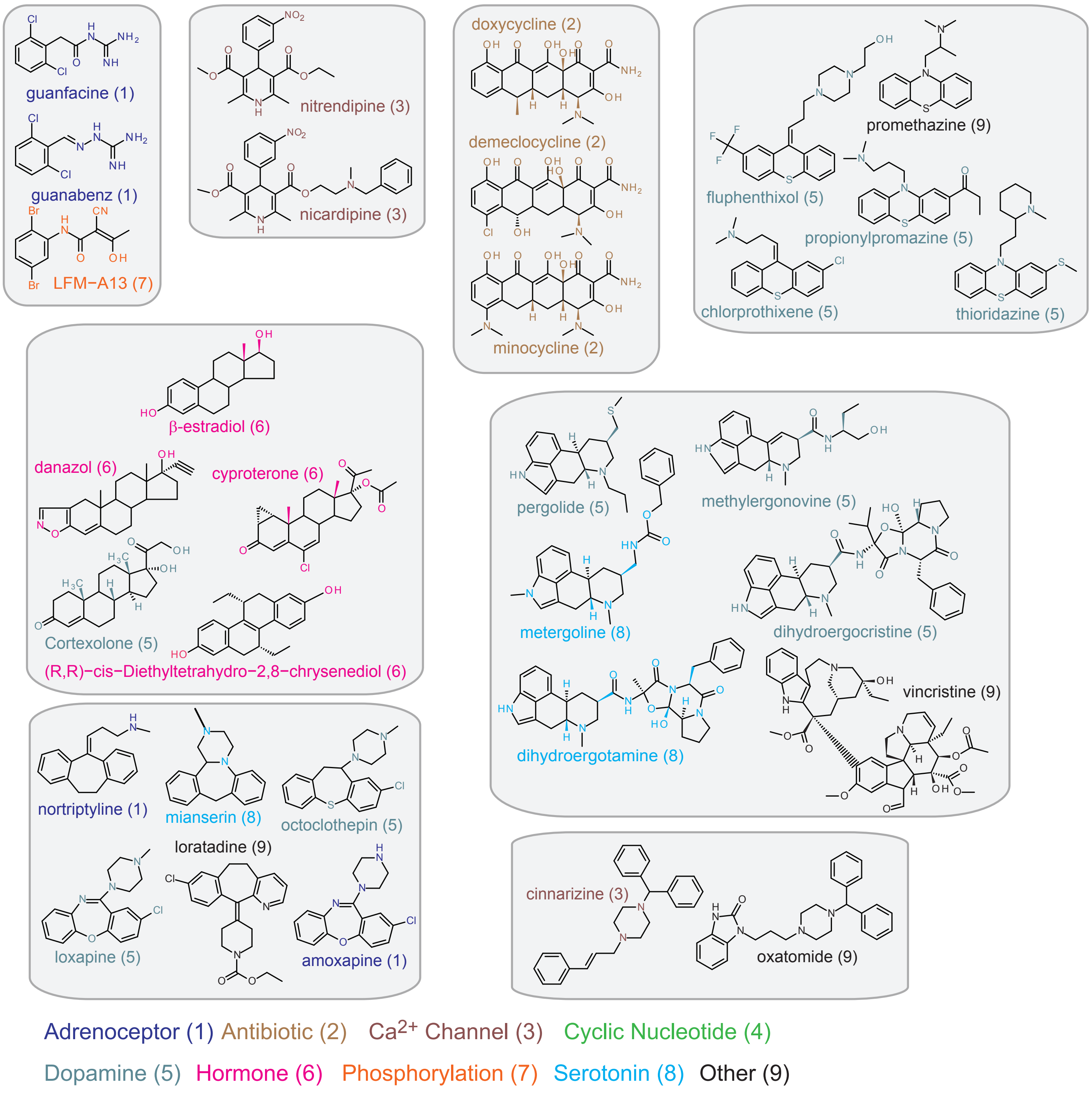

Supplementary Fig. 1. Lifespan-extending compounds show structural similarity or have common substructures. Names of the compounds are color coded and numbered according to pharmacological class, as indicated.

Supplementary Fig. 2. Structures of lifespan-extending compounds and their effects on stress resistance.

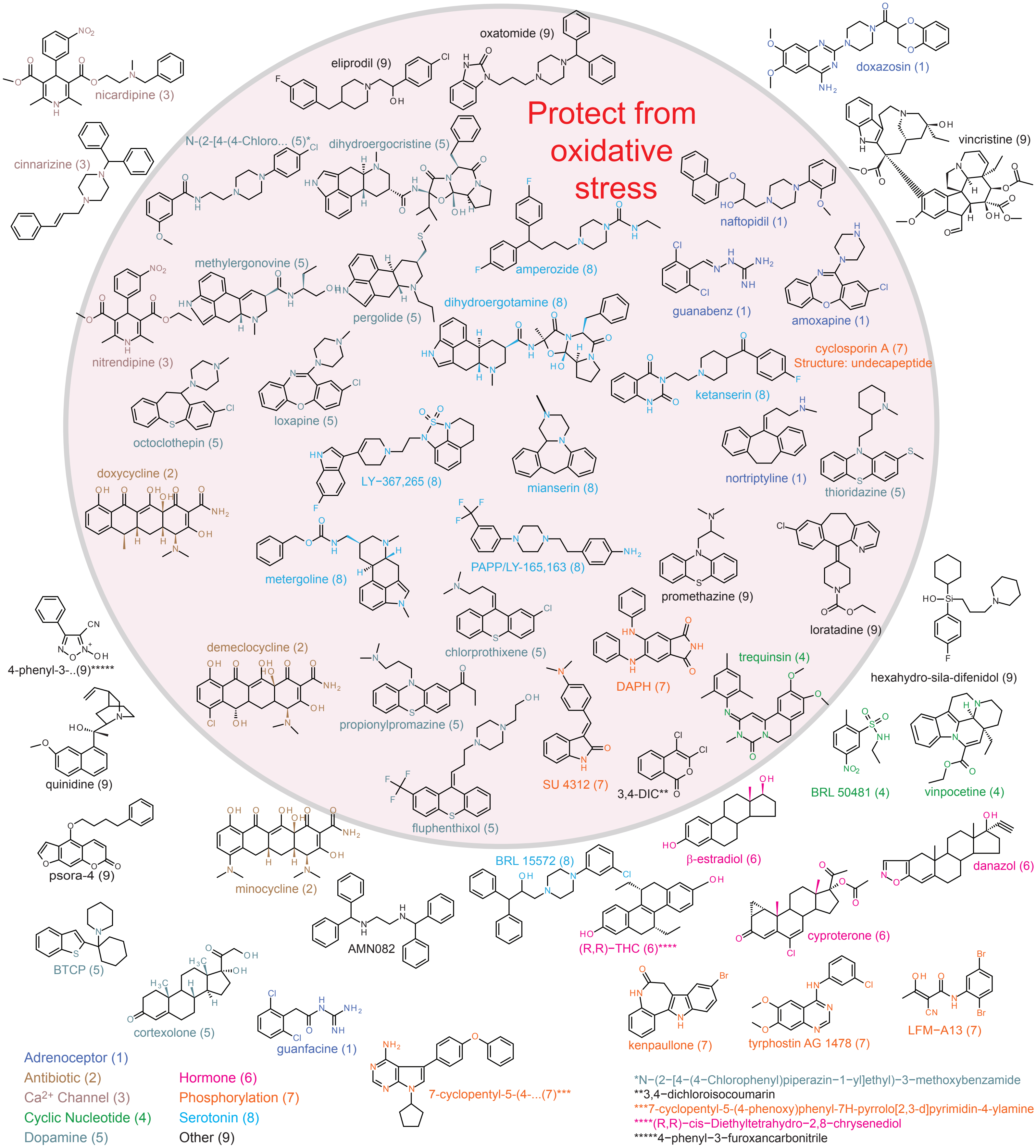

Supplementary Fig. 2. Structures of lifespan-extending compounds and their effects on stress resistance.
Structures are shown for compounds that increased *C. elegans* lifespan. Compounds in the red circle increased oxidative stress resistance in *C. elegans*. Names of compounds are color coded and numbered according to pharmacological class, as indicated. Full names for compounds marked by asterisks are shown at lower left.

Supplementary Fig. 3.
Dose response curves for lifespan- extending compounds

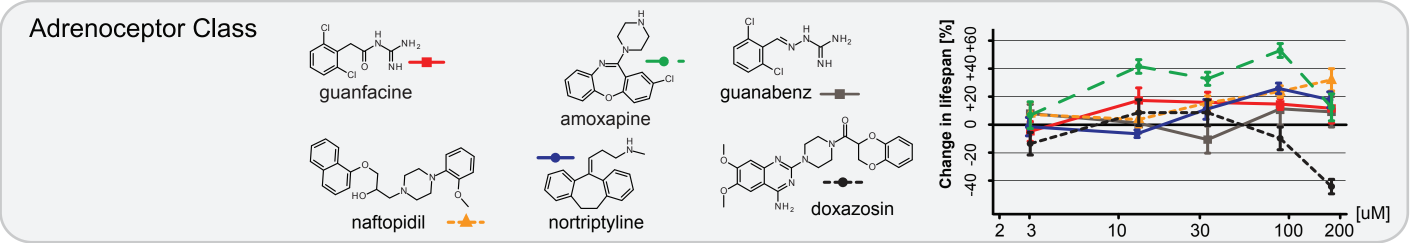

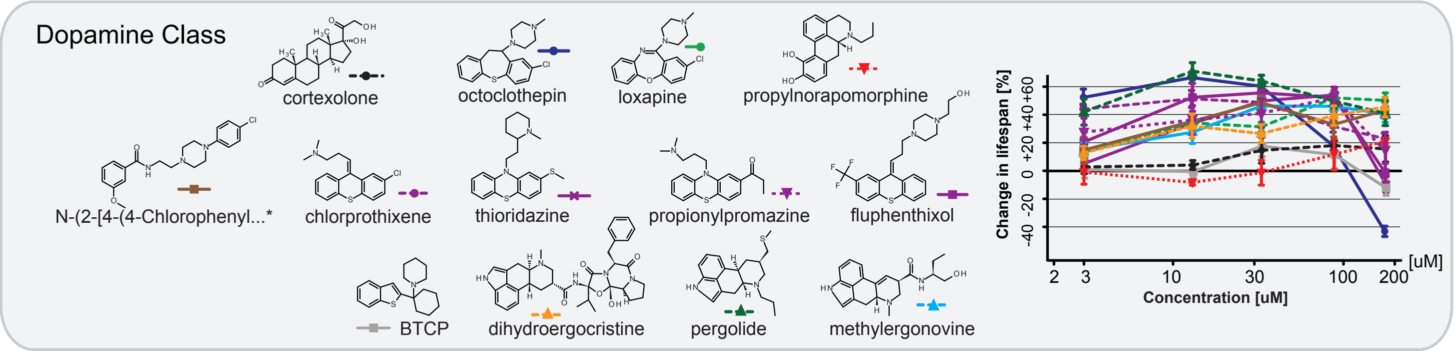

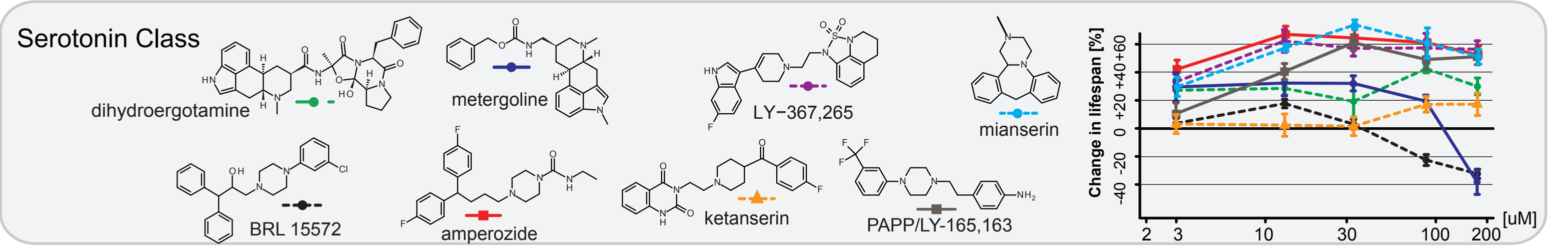

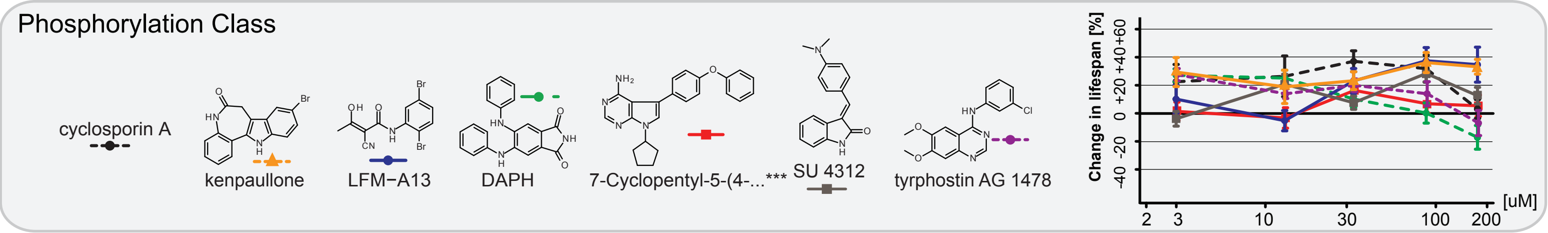

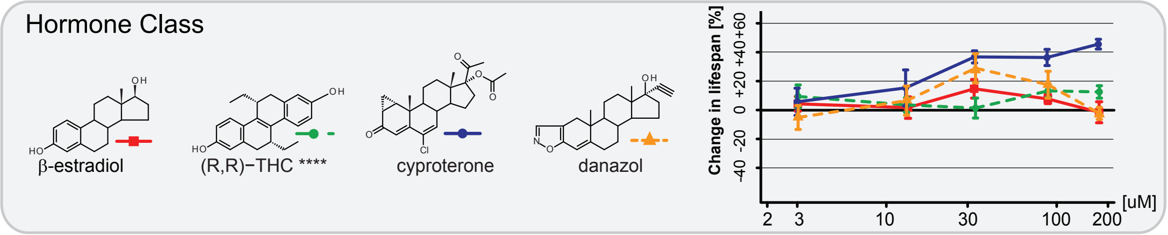

Supplementary Fig. 3. Dose response curves for lifespan- extending compounds. Graphs show percent increase in lifespan compared to vehicle control (DMSO) as a function of compound concentration [uM]. Data points indicate means of different experimental populations and error bars indicate S.E.M.. Results obtained with the compounds at left are shown in different colors, as indicated. Each compound produced a significant lifespan increase at two or more concentrations ($p<0.05$).

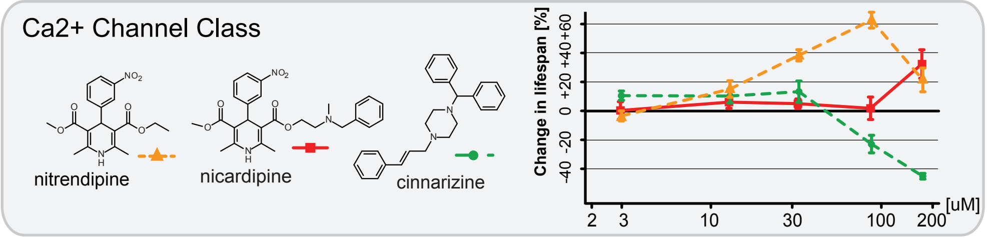

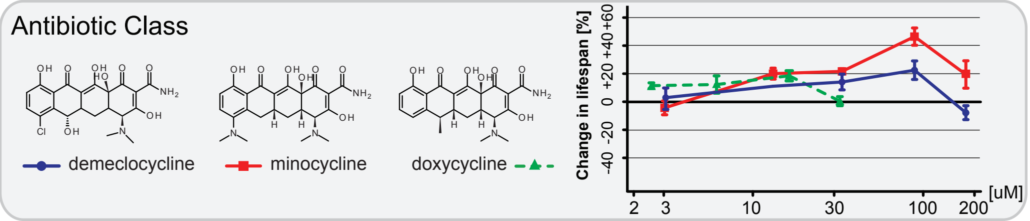

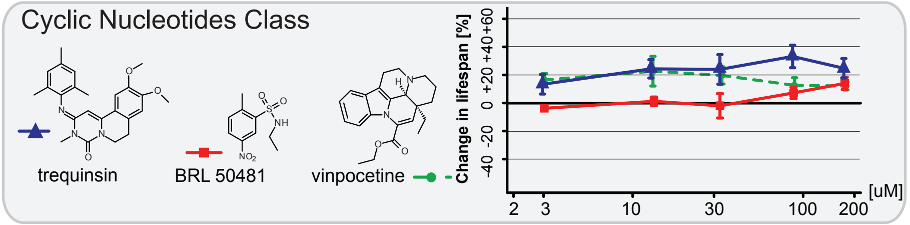

*N−(2−[4−(4−Chlorophenyl)piperazin−1−yl]ethyl)−3−methoxybenzamide
**3,4−Dichloroisocoumarin
***7-Cyclopentyl-5-(4-phenoxy)phenyl-7H-pyrrolo[2,3-d]pyrimidin-4-ylamine
****(R,R)−cis−Diethyltetrahydro−2,8−chrysenediol
*****4−Phenyl−3−furoxancarbonitrile

Supplementary Fig. 4
Effects of lifespan-extending compounds on bacterial growth

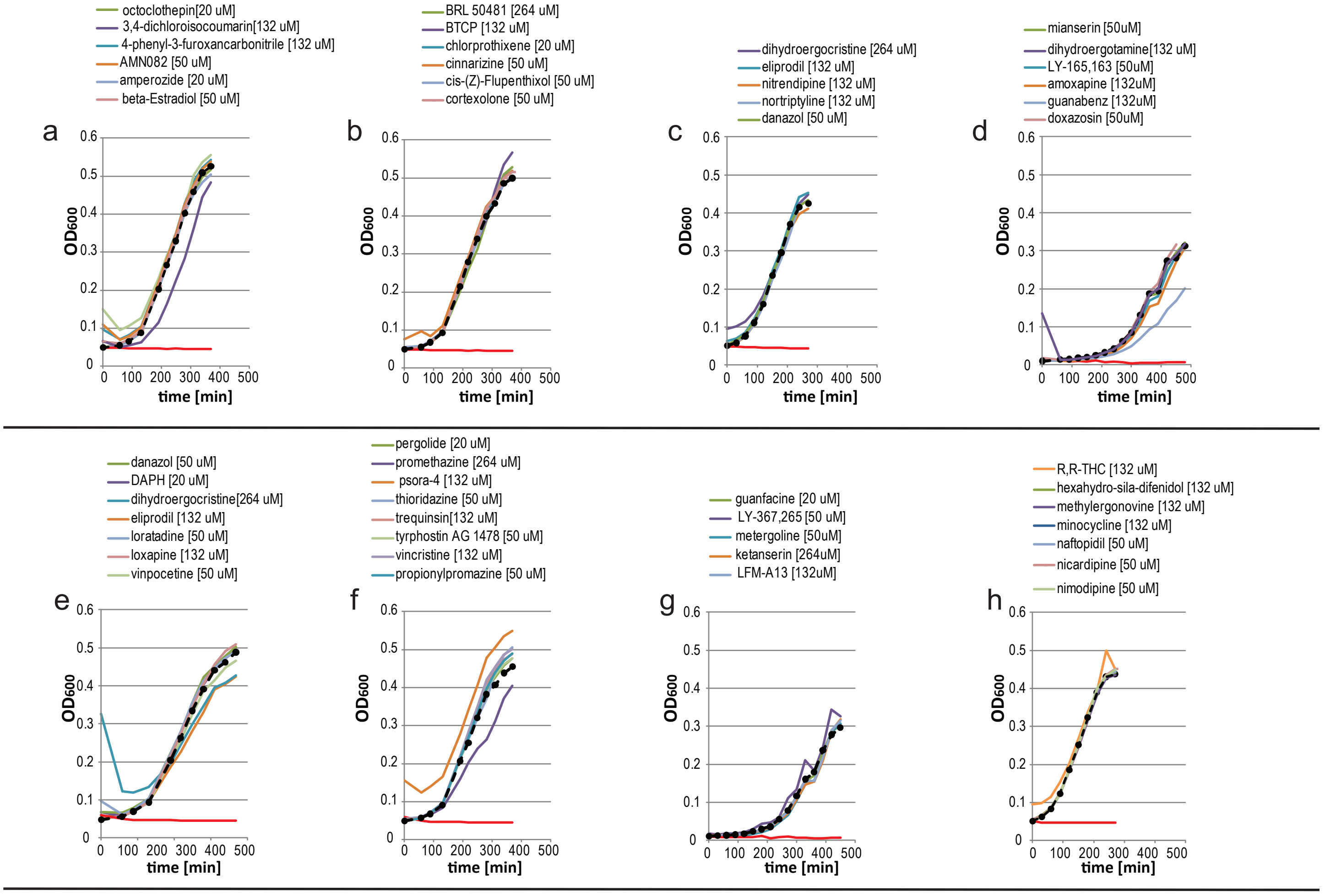

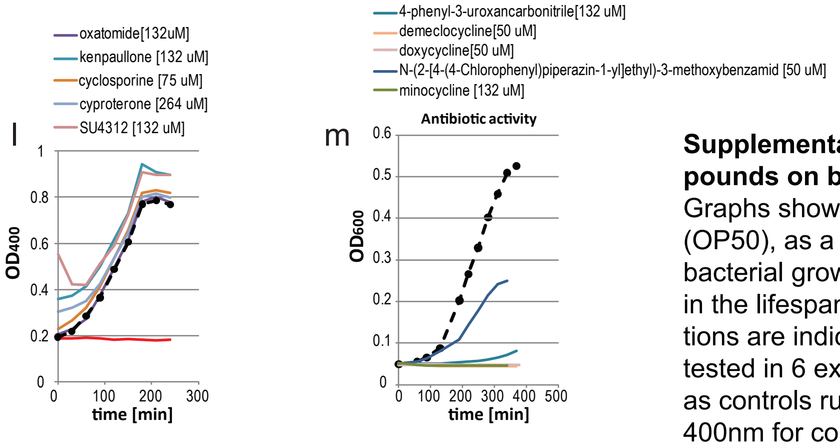

DMSO[0.5%] kanamycin [155 uM]

Supplementary Fig. 4. Effect of lifespan-extending compounds on bacterial growth
Graphs show OD600, a measurment for bacterial growth (OP50), as a function of time. The effect of each compound on bacterial growth was tested at 1.5 times the concentration used in the lifespan assay. Names of the compound and concentrations are indicated above each pannel. Each compound was tested in 6 experimental replicates with DMSO and Kanamycin as controls run in parallel. Bacterial growth was measured at 400nm for compounds that absorbed at 600nm, pannel l. The five compounds that were found to inhibit bacterial growth were retested in paralell, shown in pannel m.

Supplementary Methods
Generation of a parametric survival-time model

To be able to better interpret the outcome of our screen, we generated a parametic survival-time model describing the survival of DMSO-treated control animals. As the basis of our model, we used the Gompertz equation. Using this survival-time model we generated artificial datasets using Monte Carlo simulations. These datasets were then analyzed using our primary hit criteria to estimate our ability to detect different percent changes in lifespan.

Definitions:
T: non-negative random variable denoting the time to a failure event
F(t): cumulative distribution function: $F(t) = \Pr(T \leq t)$
S(t): survivor function: $S(t) = \Pr(T > t) = 1 - F(t)\ \ and\ for\ S(0) = 1$
Gompertz equation: $h(t) = A * e^{\gamma t}$

Equations:
(1) $h(t) = f(t)/S(t)$
(2) $F(t)' = f(t)$
(3) $F(t) = 1 - S(t) => F(t)' = f(t) = -S(t)'$
(4) $h(t) = -S(t)'/S(t)$
(5) $H(t) = \int_0^t h(t) = \int_0^t -S(u)'/S(u)\ du = -\ln(S(t))$
(6) $S(t) = \exp(-H(t))$
(7) $F(t) = 1 - e^{-H(t)}$
(8) $f(t) = h(t) * e^{-H(t)}$
(9) $H(t) = \int_0^t A * e^{-\gamma u}\ du$ [H(t) for the Gompertz equation]
(10) $H(t) = \left(\frac{A}{\gamma}\right) * (e^{\gamma t} - 1)$
(11) $S(t) = e^{-\left(\frac{A}{\gamma}\right)*(e^{\gamma t}-1)}$
(12) $f(t) = A * e^{\gamma t} * e^{-\left(\frac{A}{\gamma}\right)*(e^{\gamma t}-1)}$
(13) $F(t) = 1 - e^{-\left(\frac{A}{\gamma}\right)*(e^{\gamma t}-1)}$
(14) $Q(u) = \mathrm{F(u)}^{-1}$
(15) $ln(1 - F) = -\left(\frac{A}{\gamma}\right) * (e^{\gamma t} - 1)$
(16) $\left(\frac{\gamma}{A}\right) ln(1 - F) = 1 - e^{\gamma t}$
(17) $Q(u) = t = \left(\frac{1}{\gamma}\right) * \ln\left(1 - \left(\frac{\gamma}{A}\right) * ln(1 - u)\right)$ [Q(u) for Gompertz equation]

Maximum likelihood estimation was performed using the STATA `streg` command as described in (*1*) to estimate the parameter γ from our DMSO control population survival-time data. Using the obtained estimate for γ we generated a parametric regression survival-time model for DMSO control animals (Supplementary Fig. 5, 6)

To simulate the death times for control and drug -treated animals, the quantile function, defined as the inverse of the cumulative distribution (14) for t he Gompertz was obtained (17). Modulation of parameter *A* was used to model populations with lifespans greater than those of DMSO-treated animals. The quantile function (17) was then used for Monte Carlo simulations to generate artificial datasets of DMSO-treated, control, and long-lived populations. Population sizes used for the simulation were 30, 41 and 58 animals.

Using the logrank test (Mantel Haenzel), we analyzed these data-sets (Supplementary Fig. 7) (100,000 repetitions) to estimate the percentage of times we would be able to detect a given percent increase in lifespan using our criteria for a primary hit (alpha=0.05).

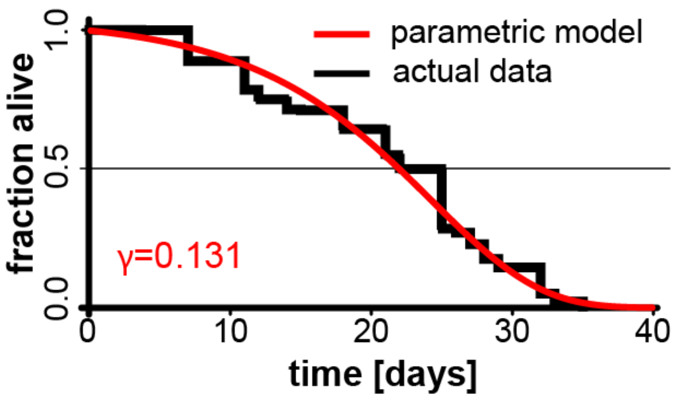

Supplementary Fig. 5: Parametric survival-time model based on the Gompertz equation

Maximum likelihood estimation was performed to generate a parametric regression survival-time model. The parameter γ was estimated from our DMSO control population survival-time data (γ =0.131). Fraction of animals alive is plotted as a function of time in days for the parametric model (red) and the actual data from the screen (black).

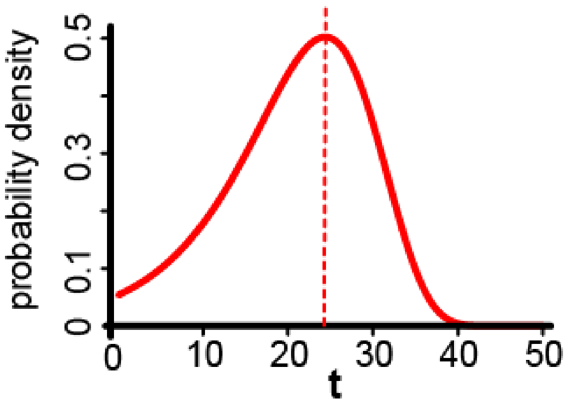

Supplementary Fig. 6: Density function f(t)

The graph plots the probability density of survival as a function of time (t) in days. The parameters for the equation were derived using DMSO control population survival time data. As can be seen, the distribution of survival times differs markedly from a normal distribution.

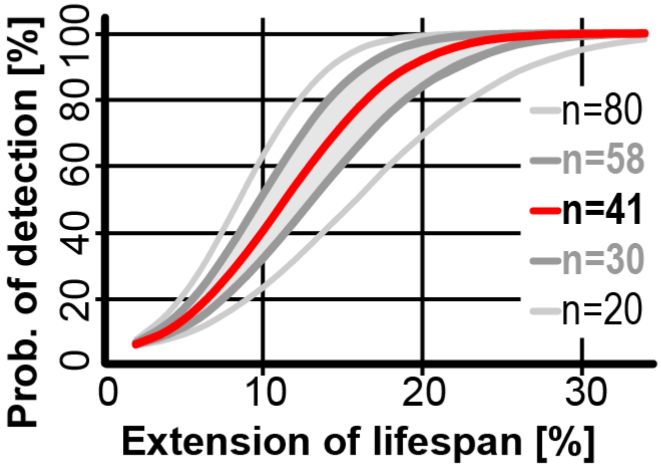

Supplementary Fig. 7: Probability of detecting % increases in lifespan

Fig.3 shows the chance of detecting a compound as a function of the percent increase in lifespan it causes using the indicated number of animals (n) in the screen. The mean number of animals used for each compound in our screen was 41. Over 90% of the compound-treated populations contained from 30 to 58 animals.

1. M. Cleves, W. Gould, R. Gutierrez, Eds., *An Introduction to Survival Analysis Using STATA*, (ed. revised edition, 2004), revised edition.

Supplementary Table 1.
Results of primary screen

* was tested a second time even though it was not in the top 156 performing compounds

** was identified to extend lifespan twice independently

FDR: False Discovery Rate

Class	Drug name	No. of animals	Percent change in [%] lifespan	Mean lifespan[days]	re-tested	Final hit	*P*-value	FDR
Adenosine	(S)-ENBA	39	0	20.2	no	no	0.760	0.997
Adenosine	1,3-Dimethyl-8-phenylxanthine	46	-4	20.2	no	no	0.681	0.997
Adenosine	1,3-Dipropyl-7-methylxanthine	39	0	20.9	no	no	0.577	0.997
Adenosine	1,3-Dipropyl-8-p-sulfophenylxanthine	39	-3	21.3	no	no	0.826	0.997
Adenosine	1,7-Dimethylxanthine	36	4	22.4	no	no	0.738	0.997
Adenosine	1-Allyl-3,7-dimethyl-8-p-sulfophenylxanthine	40	-2	21.4	no	no	0.933	0.997
Adenosine	2-Chloroadenosine	21	19	25.8	no	no	0.317	0.897
Adenosine	2-Phenylaminoadenosine	38	18	24.3	yes	no	0.024	0.235
Adenosine	3,7-Dimethyl-I-propargylxanthine	46	-15	17.9	no	no	0.078	0.492
Adenosine	3-Isobutyl-1-methylxanthine	42	-8	19.0	no	no	0.269	0.834
Adenosine	3-n-Propylxanthine	42	2	21.5	no	no	0.752	0.997
Adenosine	5'-N-Ethylcarboxamidoadenosine	34	-5	19.9	no	no	0.467	0.995
Adenosine	5'-N-Methyl carboxamidoadenosine	37	-2	21.4	no	no	0.823	0.997
Adenosine	7-Chloro-4-hydroxy-2-phenyl-1,8-naphthyridine	27	7	23.3	no	no	0.539	0.997
Adenosine	8-(3-Chlorostyryl)caffeine	35	0	21.3	no	no	0.649	0.997
Adenosine	8-(p-Sulfophenyl)theophylline	39	-1	21.7	no	no	0.958	0.997
Adenosine	8-Cyclopentyl-1,3-dimethylxanthine	42	0	21.3	no	no	0.757	0.997
Adenosine	8-Cyclopentyl-1,3-dipropylxanthine	43	-1	21.3	no	no	0.829	0.997
Adenosine	AB-MECA	39	10	24.2	no	no	0.503	0.996
Adenosine	Adenosine	28	5	22.9	no	no	0.945	0.997
Adenosine	Alloxazine	41	-4	21.0	no	no	0.429	0.977
Adenosine	Aminophylline ethylenediamine	38	1	22.9	no	no	0.900	0.997
Adenosine	Caffeine	44	-2	21.3	no	no	0.709	0.997
Adenosine	CGS-15943	38	-5	20.4	no	no	0.916	0.997
Adenosine	CGS-21680 hydrochloride	46	-3	20.9	no	no	0.884	0.997
Adenosine	Chloro-IB-MECA	44	-10	19.3	no	no	0.500	0.996
Adenosine	Dilazep hydrochloride	36	9	23.3	no	no	0.311	0.893
Adenosine	Dipyridamole	41	-10	18.9	no	no	0.276	0.843
Adenosine	erythro-9-(2-Hydroxy-3-nonyl)adenine hydrochloride	31	13	22.9	no	no	0.609	0.997
Adenosine	Etazolate hydrochloride	45	-7	19.4	no	no	0.489	0.996
Adenosine	FSCPX	26	18	23.8	no	no	0.189	0.743
Adenosine	GR 79236X	41	3	22.3	no	no	0.910	0.997
Adenosine	HE-NECA	42	16	23.1	yes	no	0.099	0.560
Adenosine	IB-MECA	37	-18	17.0	no	no	0.172	0.717
Adenosine	Metrifudil	49	1	21.4	no	no	0.662	0.997
Adenosine	MRS 1523	31	4	21.4	no	no	0.503	0.996
Adenosine	MRS 1754	41	4	21.4	no	no	0.844	0.997

Adenosine	MRS 2211 sodium salt hydrate	35	2	22.3	no	no	0.980	0.997
Adenosine	N6-2-(4-Aminophenyl)ethyladenosine	33	-4	21.0	no	no	0.671	0.997
Adenosine	N6-2-Phenylethyladenosine	35	5	21.6	no	no	0.393	0.951
Adenosine	N6-Cyclohexyladenosine	45	-4	20.6	no	no	0.979	0.997
Adenosine	N6-Cyclopentyl-9-methyladenine	42	11	23.5	no	no*	0.153	0.681
Adenosine	N6-Cyclopentyladenosine	40	26	27.4	yes	no	0.023	0.232
Adenosine	N6-Methyladenosine	46	3	21.2	no	no	0.788	0.997
Adenosine	N6-Phenyladenosine	46	-4	19.8	no	no	0.680	0.997
Adenosine	Propentofylline	35	-3	19.9	no	no	0.687	0.997
Adenosine	R(-)-N6-(2-Phenylisopropyl)adenosine	40	-3	20.4	no	no	0.619	0.997
Adenosine	S-(4-Nitrobenzyl)-6-thioguanosine	41	9	24.8	no	no	0.158	0.691
Adenosine	S-(4-Nitrobenzyl)-6-thioinosine	37	0	21.2	no	no	0.811	0.997
Adenosine	Theobromine	36	-2	20.8	no	no	0.983	0.997
Adenosine	Theophylline	50	0	21.2	no	no	0.119	0.606
Adenosine	VUF 5574	43	-16	17.5	no	no	0.001	0.020
Adenosine	Xanthine amine congener	31	-6	19.7	no	no	0.350	0.925
Adrenergic	S(-)-Pindolol	38	0	21.4	no	no	0.720	0.997
Adrenoceptor	(-)-alpha-Methylnorepinephrine	33	-3	20.8	no	no	0.971	0.997
Adrenoceptor	(-)-Ephedrine hemisulfate	38	11	22.5	no	no	0.276	0.843
Adrenoceptor	(-)-Epinephrine bitartrate	49	6	21.4	no	no	0.697	0.997
Adrenoceptor	(-)-Isoproterenol hydrochloride	59	-3	20.0	no	no	0.983	0.997
Adrenoceptor	(±)-Atenolol	37	10	24.0	no	no	0.419	0.968
Adrenoceptor	(±)-CGP-12177A hydrochloride	50	7	22.9	no	no	0.340	0.917
Adrenoceptor	(±)-Epinephrine hydrochloride	38	10	22.3	no	no	0.321	0.903
Adrenoceptor	(±)-Isoproterenol hydrochloride	45	0	20.7	no	no	0.780	0.997
Adrenoceptor	(±)-Metoprolol (+)-tartrate	36	5	21.5	no	no	0.710	0.997
Adrenoceptor	(±)-Norepinephrine (+)bitartrate	45	-6	21.5	no	no	0.823	0.997
Adrenoceptor	(±)-Normetanephrine hydrochloride	48	6	22.4	no	no	0.627	0.997
Adrenoceptor	(±)-Octopamine hydrochloride	40	-13	18.4	no	no	0.031	0.274
Adrenoceptor	(±)-Propranolol hydrochloride	46	7	22.7	yes	no	0.115	0.595
Adrenoceptor	(±)-Sotalol hydrochloride	24	-2	20.5	no	no	0.728	0.997
Adrenoceptor	(±)-Synephrine	40	-6	19.6	no	no	0.031	0.270
Adrenoceptor	(±)-Vanillylmandelic acid	46	-2	19.6	no	no	0.796	0.997
Adrenoceptor	(S)-(-)-propafenone hydrochloride	39	-4	19.7	no	no	0.751	0.997
Adrenoceptor	(S)-Propranolol hydrochloride	31	14	23.4	no	no	0.175	0.721
Adrenoceptor	6-Fluoronorepinephrine hydrochloride	47	-2	21.2	no	no	0.579	0.997
Adrenoceptor	6-Hydroxy-DL-DOPA	42	-9	18.1	no	no	0.513	0.996
Adrenoceptor	A-315456	44	-9	20.7	no	no	0.264	0.830
Adrenoceptor	Albuterol hemisulfate	45	-6	19.6	no	no	0.165	0.705
Adrenoceptor	Alprenolol hydrochloride	43	1	22.1	no	no	0.577	0.997
Adrenoceptor	Amiodarone hydrochloride	48	-50	10.8	no	no	0.000	0.000
Adrenoceptor	Amitriptyline hydrochloride	34	20	26.3	yes	no	0.091	0.533
Adrenoceptor	Amoxapine	40	39	30.4	yes	yes	0.000	0.001
Adrenoceptor	Benoxathian hydrochloride	42	6	23.0	no	no	0.327	0.904
Adrenoceptor	Betaxolol hydrochloride	34	-9	19.8	no	no	0.367	0.937
Adrenoceptor	B-HT 933 dihydrochloride	35	-8	19.9	no	no	0.199	0.755

Adrenoceptor	Bisoprolol hemifumarate salt	29	4	21.9	no	no	0.487	0.996
Adrenoceptor	Bretylium tosylate	44	-4	20.9	no	no	0.761	0.997
Adrenoceptor	BRL 37344 sodium	47	-8	20.0	no	no	0.570	0.997
Adrenoceptor	Bromoacetyl alprenolol menthane	48	-21	17.0	no	no	0.007	0.103
Adrenoceptor	CGP 20712A methanesulfonate	41	3	22.1	no	no	0.345	0.923
Adrenoceptor	Chloroethylclonidine dihydrochloride	43	-4	20.7	no	no	0.914	0.997
Adrenoceptor	Cirazoline hydrochloride	52	7	22.8	yes	no	0.102	0.560
Adrenoceptor	CL 316,243	35	6	22.9	no	no	0.557	0.997
Adrenoceptor	Clonidine hydrochloride	40	3	22.5	no	no	0.631	0.997
Adrenoceptor	Desipramine hydrochloride	50	7	23.0	no	no	0.314	0.897
Adrenoceptor	Dobutamine hydrochloride	44	8	23.1	no	no	0.524	0.997
Adrenoceptor	Doxazosin mesylate	38	11	23.2	yes	yes	0.017	0.186
Adrenoceptor	Doxepin hydrochloride	37	15	24.7	yes	no	0.017	0.186
Adrenoceptor	DSP-4 hydrochloride	40	2	22.1	no	no	0.806	0.997
Adrenoceptor	Fenoterol hydrobromide	55	5	21.2	no	no	0.670	0.997
Adrenoceptor	Fenspiride hydrochloride	34	4	21.1	no	no	0.777	0.997
Adrenoceptor	Fiduxosin hydrochloride	32	-11	18.1	no	no	0.282	0.853
Adrenoceptor	Formoterol	37	2	20.7	no	no	0.379	0.946
Adrenoceptor	Guanabenz acetate	43	19	23.6	yes	yes	0.030	0.269
Adrenoceptor	Guanfacine hydrochloride	43	27	25.7	yes	yes	0.014	0.168
Adrenoceptor	ICI 118,551 hydrochloride	44	0	20.8	no	no	0.791	0.997
Adrenoceptor	Imiloxan hydrochloride	37	0	20.8	no	no	0.918	0.997
Adrenoceptor	Isotharine mesylate	35	0	20.6	no	no	0.738	0.997
Adrenoceptor	L(-)-Norepinephrine bitartrate	38	6	23.1	no	no	0.574	0.997
Adrenoceptor	L-765,314	36	3	21.9	yes	no	0.017	0.186
Adrenoceptor	Labetalol hydrochloride	31	11	22.9	no	no	0.307	0.885
Adrenoceptor	Maprotiline hydrochloride	35	10	23.4	yes	no	0.103	0.560
Adrenoceptor	Metaproterenol hemisulfate	44	2	21.0	no	no	0.659	0.997
Adrenoceptor	Methoxamine hydrochloride	37	3	21.1	no	no	0.786	0.997
Adrenoceptor	MHPG piperazine	36	11	22.0	no	no	0.189	0.743
Adrenoceptor	MHPG sulfate potassium	44	11	22.2	no	no	0.389	0.950
Adrenoceptor	MK-912	48	8	22.2	yes	no	0.058	0.421
Adrenoceptor	Moxisylyte hydrochloride	52	-8	19.0	no	no	0.426	0.973
Adrenoceptor	Moxonidine hydrochloride	45	0	20.6	no	no	0.735	0.997
Adrenoceptor	Naftopidil dihydrochloride	30	14	24.0	yes	yes	0.018	0.187
Adrenoceptor	Naphazoline hydrochloride	34	2	21.7	no	no	0.740	0.997
Adrenoceptor	Nisoxetine hydrochloride	49	0	21.0	no	no	0.791	0.997
Adrenoceptor	Nortriptyline hydrochloride	41	26	26.7	yes	yes	0.000	0.001
Adrenoceptor	Nylidrin hydrochloride	41	-12	18.5	no	no	0.298	0.871
Adrenoceptor	Oxymetazoline hydrochloride	41	-6	19.8	no	no	0.975	0.997
Adrenoceptor	p-Aminoclonidine hydrochloride	46	-6	21.3	no	no	0.395	0.951
Adrenoceptor	Phenoxybenzamine hydrochloride	53	-4	20.9	no	no	0.713	0.997
Adrenoceptor	Phentolamine mesylate	33	3	21.1	no	no	0.839	0.997
Adrenoceptor	Phenylephrine hydrochloride	32	-5	20.0	no	no	0.118	0.606
Adrenoceptor	Pindolol	52	-11	18.8	no	no	0.058	0.421
Adrenoceptor	p-Iodoclonidine hydrochloride	46	5	21.7	no	no	0.442	0.985

Adrenoceptor	Prazosin hydrochloride	38	6	21.8	no	no	0.223	0.787
Adrenoceptor	Protriptyline hydrochloride	36	11	22.8	no	no	0.228	0.787
Adrenoceptor	R(-)-Denopamine	48	-6	19.6	no	no	0.903	0.997
Adrenoceptor	R(-)-Isoproterenol (+)-bitartrate	47	6	21.0	no	no	0.496	0.996
Adrenoceptor	R(+)-Atenolol	41	1	22.2	no	no	0.742	0.997
Adrenoceptor	Rauwolscine hydrochloride	37	16	24.1	no	no	0.144	0.661
Adrenoceptor	Ritodrine hydrochloride	37	6	22.1	no	no	0.907	0.997
Adrenoceptor	RX 821002 hydrochloride	47	-1	20.7	no	no	0.840	0.997
Adrenoceptor	S(-)-Atenolol	39	-2	21.5	no	no	0.703	0.997
Adrenoceptor	S(-)-Timolol maleate	43	-2	20.8	no	no	0.606	0.997
Adrenoceptor	S(+)-Isoproterenol (+)-bitartrate	44	1	21.0	no	no	0.764	0.997
Adrenoceptor	Salbutamol	41	0	20.8	no	no	0.869	0.997
Adrenoceptor	Salmeterol xinafoate	38	-12	18.3	no	no	0.098	0.560
Adrenoceptor	SKF 86466	38	2	21.3	no	no	0.469	0.995
Adrenoceptor	SR 59230A oxalate	37	-3	20.7	no	no	0.271	0.836
Adrenoceptor	Terazosin hydrochloride	40	-5	20.2	no	no	0.384	0.948
Adrenoceptor	Terbutaline hemisulfate	42	3	21.8	no	no	0.492	0.996
Adrenoceptor	Tetrahydrozoline hydrochloride	27	14	24.2	no	no	0.563	0.997
Adrenoceptor	Tomoxetine	31	0	20.9	no	no	0.705	0.997
Adrenoceptor	Tulobuterol hydrochloride	47	0	21.2	no	no	0.851	0.997
Adrenoceptor	UK 14,304	41	5	22.0	no	no	0.457	0.990
Adrenoceptor	Urapidil hydrochloride	41	9	22.8	no	no	0.334	0.908
Adrenoceptor	Urapidil, 5-Methyl-	51	-3	20.2	no	no	0.585	0.997
Adrenoceptor	WB-4101 hydrochloride	26	-4	20.6	no	no	0.704	0.997
Adrenoceptor	Xamoterol hemifumarate	46	12	23.3	no	no	0.377	0.944
Adrenoceptor	Xylazine hydrochloride	28	14	23.8	no	no	0.178	0.726
Adrenoceptor	Xylometazoline hydrochloride	40	7	22.3	no	no	0.624	0.997
Adrenoceptor	Yohimbine hydrochloride	37	8	22.5	no	no	0.316	0.897
Angiogenesis	DL-alpha-Difluoromethylornithine hydrochloride	40	-4	20.2	no	no	0.841	0.997
Antibiotic	Aminopterin	32	8	24.6	no	no	0.513	0.996
Antibiotic	Cefaclor	42	0	21.8	no	no	0.754	0.997
Antibiotic	Cefazolin sodium	39	-9	19.8	no	no	0.077	0.487
Antibiotic	Cefmetazole sodium	42	-5	20.6	no	no	0.618	0.997
Antibiotic	Cefotaxime sodium	56	7	23.2	yes	no	0.114	0.595
Antibiotic	Cefsulodin sodium salt hydrate	43	8	23.4	no	no	0.343	0.920
Antibiotic	Ceftriaxone sodium	41	28	27.8	yes	no	0.002	0.034
Antibiotic	Cephalexin hydrate	38	12	24.3	yes	no	0.103	0.560
Antibiotic	Cephalosporin C zinc salt	41	0	21.8	no	no	0.684	0.997
Antibiotic	Cephalothin sodium	36	6	22.9	no	no	0.722	0.997
Antibiotic	Cephapirin sodium	47	5	22.8	no	no	0.805	0.997
Antibiotic	Cephradine	28	10	23.9	no	no	0.583	0.997
Antibiotic	Cinoxacin	42	1	21.9	no	no	0.941	0.997
Antibiotic	Demeclocycline hydrochloride	43	16	24.4	yes	yes	0.089	0.530
Antibiotic	Doxycycline hydrochloride	38	29	27.0	yes	yes	0.018	0.187
Antibiotic	Lomefloxacin hydrochloride	37	12	23.2	yes	no	0.128	0.627
Antibiotic	Mevastatin	34	-2	20.2	no	no	0.665	0.997

Antibiotic	Minocycline hydrochloride	27	15	24.4	yes	yes	0.052	0.394
Antibiotic	Nalidixic acid sodium	44	9	23.1	yes	no	0.015	0.174
Antibiotic	Niclosamide	36	-21	16.7	no	no	0.000	0.000
Antibiotic	Ofloxacin	49	8	22.9	no	no	0.212	0.776
Antibiotic	Oxolinic acid	45	8	22.7	no	no	0.255	0.820
Antibiotic	Paromomycin sulfate	35	5	21.5	no	no	0.947	0.997
Antibiotic	Phosphomycin disodium	46	-7	19.7	no	no	0.616	0.997
Antibiotic	Praziquantel	27	-5	20.2	no	no	0.465	0.995
Antibiotic	Pyrazinecarboxamide	50	-2	20.1	no	no	0.778	0.997
Antibiotic	Thiolactomycin	47	2	21.5	no	no	0.470	0.995
Antibiotic	Trimethoprim	41	14	23.8	yes	no	0.028	0.255
Antibiotic	Vancomycin hydrochloride	44	5	21.9	no	no	0.252	0.817
Anticonvulsant	5,5-Diphenylhydantoin	33	-1	21.1	no	no	0.898	0.997
Anticonvulsant	Carbamazepine	52	2	22.2	no	no	0.728	0.997
Anticonvulsant	Ethosuximide	45	3	20.9	no	no	0.797	0.997
Anticonvulsant	Foliosidine	48	-4	19.5	no	no	0.609	0.997
Anticonvulsant	Gabapentin	44	-2	19.5	no	no	0.983	0.997
Anticonvulsant	Lamotrigine	40	-3	20.2	no	no	0.718	0.997
Anticonvulsant	N-(4-Amino-2-chlorophenyl)phthalimide	40	4	22.7	no	no	0.789	0.997
Anticonvulsant	Phenytoin sodium	31	-5	20.4	no	no	0.959	0.997
Anticonvulsant	Primidone	36	7	22.0	no	no	0.894	0.997
Anticonvulsant	Valproic acid sodium	48	-2	20.7	no	no	0.947	0.997
Anticonvulsant	Zonisamide sodium	32	-4	20.0	no	no	0.455	0.990
Apoptosis	(S)-(+)-Camptothecin	32	8	23.1	no	no	0.875	0.997
Apoptosis	1,5-Isoquinolinediol	42	0	20.8	no	no	0.894	0.997
Apoptosis	3-aminobenzamide	49	-12	20.1	no	no	0.162	0.700
Apoptosis	4-Amino-1,8-naphthalimide	35	-4	21.9	no	no	0.375	0.944
Apoptosis	Aurintricarboxylic acid	52	-6	21.4	no	no	0.657	0.997
Apoptosis	Benzamide	43	-4	20.9	no	no	0.881	0.997
Apoptosis	beta-Lapachone	38	-8	19.1	no	no	0.058	0.422
Apoptosis	Emetine dihydrochloride hydrate	37	9	22.8	no	no	0.188	0.743
Apoptosis	Etoposide	31	2	21.3	no	no	0.946	0.997
Apoptosis	Imiquimod	35	3	21.5	no	no	0.566	0.997
Apoptosis	m-Iodobenzylguanidine hemisulfate	38	-7	19.3	no	no	0.392	0.951
Apoptosis	Pifithrin-mu	35	-1	21.0	no	no	0.461	0.993
Apoptosis	Retinoic acid	32	1	21.1	no	no	0.807	0.997
Apoptosis	Sobuzoxane	33	6	22.1	no	no	0.413	0.966
Apoptosis	XK469	46	8	22.5	no	no	0.412	0.966
Benzodiazepine	3-Methyl-6-(3-[trifluoromethyl]phenyl)-1,2,4-triazolo[4,3-b]pyridazine	37	-3	19.9	no	no	0.239	0.789
Benzodiazepine	CB34	35	5	22.9	no	no	0.615	0.997
Benzodiazepine	Flumazenil	36	6	21.5	no	no	0.586	0.997
Benzodiazepine	L-655,708	22	20	24.8	yes	no	0.073	0.471
Benzodiazepine	Methyl 6,7-dimethoxy-4-ethyl-beta-carboline-3-carboxylate	47	17	23.8	yes	no	0.038	0.316
Benzodiazepine	Methyl beta-carboline-3-carboxylate	30	3	20.8	no	no	0.688	0.997

Benzodiazepine	N,N-Dihexyl-2-(4-fluorophenyl)indole-3-acetamide	37	20	25.2	yes	no	0.043	0.351
Biochemistry	(±)-p-Aminoglutethimide	34	17	25.7	yes	no	0.104	0.563
Biochemistry	1,10-Phenanthroline monohydrate	40	-19	16.6	no	no	0.000	0.003
Biochemistry	1-Deoxynojirimycin hydrochloride	38	-2	20.5	no	no	0.636	0.997
Biochemistry	2,2'-Bipyridyl	31	6	22.3	no	no	0.560	0.997
Biochemistry	2,4-Dinitrophenyl 2-fluoro-2-deoxy-beta-D-glucopyranoside	40	11	23.7	no	no	0.213	0.776
Biochemistry	2-Chloro-2-deoxy-D-glucose	30	8	23.2	no	no	0.615	0.997
Biochemistry	3,4-Dichloroisocoumarin	29	28	26.9	yes	yes	0.052	0.394
Biochemistry	3-Hydroxybenzylhydrazine dihydrochloride	44	1	20.1	no	no	0.692	0.997
Biochemistry	4-(2-Aminoethyl)benzenesulfonyl fluoride hydrochloride	33	1	22.0	no	no	0.922	0.997
Biochemistry	4-Aminobenzamidine dihydrochloride	53	-8	20.9	no	no	0.623	0.997
Biochemistry	4-Chloromercuribenzoic acid	43	-46	11.8	no	no	0.000	0.000
Biochemistry	4-Hydroxybenzhydrazide	47	10	21.9	no	no	0.197	0.754
Biochemistry	4-Methylpyrazole hydrochloride	48	2	20.9	no	no	0.905	0.997
Biochemistry	Acetamide	31	1	23.1	no	no	0.832	0.997
Biochemistry	Acetazolamide	36	-13	19.7	no	no	0.089	0.530
Biochemistry	Actinonin	43	-2	22.3	no	no	0.610	0.997
Biochemistry	Benserazide hydrochloride	35	2	22.1	no	no	0.976	0.997
Biochemistry	Benzamidine hydrochloride	43	5	22.8	no	no	0.790	0.997
Biochemistry	Bestatin hydrochloride	33	-10	19.5	no	no	0.370	0.939
Biochemistry	beta-Chloro-L-alanine hydrochloride	42	1	22.1	no	no	0.921	0.997
Biochemistry	Betaine hydrochloride	32	12	24.3	no	no	0.324	0.904
Biochemistry	Chlorothiazide	51	-3	21.2	no	no	0.985	0.997
Biochemistry	Diethylenetriaminepentaacetic acid	49	9	22.9	no	no	0.183	0.737
Biochemistry	E-64	42	-3	20.3	no	no	0.839	0.997
Biochemistry	Epibestatin hydrochloride	42	-2	20.6	no	no	0.786	0.997
Biochemistry	Ethylene glycol-bis(2-aminoethylether)-N,N,N',N'-tetraacetic acid	49	-5	19.1	no	no	0.614	0.997
Biochemistry	Furafylline	38	-9	18.4	no	no	0.648	0.997
Biochemistry	Hydrochlorothiazide	37	14	22.8	yes	no	0.108	0.571
Biochemistry	Iodoacetamide	55	10	22.0	no	no	0.129	0.630
Biochemistry	L-allylglycine	42	-11	19.5	no	no	0.098	0.560
Biochemistry	L-alpha-Methyl DOPA	37	10	23.2	yes	no	0.126	0.624
Biochemistry	L-azetidine-2-carboxylic acid	47	-6	21.5	no	no	0.445	0.985
Biochemistry	L-Leucinethiol, oxidized dihydrochloride	38	5	21.8	no	no	0.723	0.997
Biochemistry	N-Ethylmaleimide	47	1	20.4	no	no	0.693	0.997
Biochemistry	N-p-Tosyl-L-phenylalanine chloromethyl ketone	52	2	21.6	no	no	0.663	0.997
Biochemistry	O-(Carboxymethyl)hydroxylamine hemihydrochloride	43	-6	21.5	no	no	0.706	0.997
Biochemistry	P1,P4-Di(adenosine-5')tetraphosphate triammonium	40	-14	18.4	no	no	0.089	0.530
Biochemistry	PD 404,182	37	0	21.1	no	no	0.563	0.997
Biochemistry	Phosphoramidon disodium	43	0	20.9	no	no	0.973	0.997
Biochemistry	S-(-)-Carbidopa	33	-2	21.0	no	no	0.706	0.997

Biochemistry	Sodium Oxamate	44	-13	18.5	no	no	0.181	0.733
Biochemistry	Tetraethylthiuram disulfide	36	-3	20.6	no	no	0.805	0.997
Biochemistry	Tetraisopropyl pyrophosphoramide	41	-6	19.9	no	no	0.940	0.997
Biochemistry	Z-L-Phe chloromethyl ketone	35	-3	21.0	no	no	0.872	0.997
Ca2+ Channel	(±)-Bay K 8644	31	12	24.2	no	no	0.269	0.834
Ca2+ Channel	(±)-Methoxyverapamil hydrochloride	29	-6	19.3	no	no	0.297	0.871
Ca2+ Channel	(±)-Verapamil hydrochloride	35	12	23.5	no	no	0.204	0.760
Ca2+ Channel	Bepridil hydrochloride	41	-6	20.4	no	no	0.549	0.997
Ca2+ Channel	Cilnidipine	35	-8	19.3	no	no	0.926	0.997
Ca2+ Channel	Cinnarizine	33	11	24.1	yes	yes	0.018	0.187
Ca2+ Channel	Diltiazem hydrochloride	45	-2	21.1	no	no	0.982	0.997
Ca2+ Channel	Felodipine	39	-54	9.3	no	no	0.000	0.000
Ca2+ Channel	FPL 64176	41	-8	18.6	no	no	0.478	0.996
Ca2+ Channel	Mibefradil dihydrochloride	29	-4	19.7	no	no	0.637	0.997
Ca2+ Channel	MRS 1845	25	0	20.5	no	no	1.000	1.000
Ca2+ Channel	Nicardipine hydrochloride	31	20	25.4	yes	yes	0.002	0.027
Ca2+ Channel	Nifedipine	46	6	22.4	no	no	0.199	0.755
Ca2+ Channel	Nimodipine	31	7	22.6	yes	no	0.066	0.451
Ca2+ Channel	Nitrendipine	42	12	23.6	yes	yes	0.009	0.120
Ca2+ Channel	NNC 55-0396	43	-26	15.2	no	no	0.021	0.215
Ca2+ Channel	Phloretin	39	-1	20.4	no	no	0.606	0.997
Ca2+ Channel	SKF 96365	27	-3	20.2	no	no	0.982	0.997
Ca2+ Channel	YS-035 hydrochloride	39	12	23.4	yes	no	0.065	0.448
Cannabinoid	(R)-(+)-WIN 55,212-2 mesylate	38	8	22.5	no	no	0.462	0.993
Cannabinoid	CP55940	40	-1	21.4	no	no	0.596	0.997
Cannabinoid	JWH-015	40	-6	19.5	no	no	0.891	0.997
Cannabinoid	N-arachidonylglycine	41	8	24.5	no	no	0.166	0.705
Cannabinoid	Palmitoylethanolamide	41	-8	19.5	no	no	0.183	0.737
Cell Cycle	5-Fluorouracil	40	5	21.3	no	no	0.840	0.997
Cell Cycle	Apigenin	28	3	23.5	no	no	0.865	0.997
Cell Cycle	Bay 11-7085	41	-2	21.2	no	no	0.491	0.996
Cell Cycle	Caffeic acid phenethyl ester	36	3	22.5	no	no	0.675	0.997
Cell Cycle	Daidzein	42	-1	20.7	no	no	0.960	0.997
Cell Cycle	Ellipticine	33	8	21.9	no	no	0.234	0.787
Cell Cycle	Fusidic acid sodium	40	1	20.4	no	no	0.926	0.997
Cell Cycle	Ganciclovir	40	-9	18.4	no	no	0.210	0.773
Cell Cycle	MDL 28170	46	0	20.6	no	no	0.958	0.997
Cell Cycle	Pyrocatechol	35	8	23.5	no	no	0.338	0.914
Cell Cycle	Retinoic acid p-hydroxyanilide	41	-3	19.2	no	no	0.820	0.997
Cell Cycle	Ribavirin	40	-12	18.5	no	no	0.403	0.957
Cell Cycle	Se-(methyl)selenocysteine hydrochloride	55	-64	7.3	no	no	0.000	0.000
Cell Cycle	SU 9516	38	7	22.5	no	no	0.793	0.997
Cell Cycle	TG003	39	-3	20.4	no	no	0.262	0.827
Cell Stress	(+)-Catechin Hydrate	40	8	23.5	no	no	0.186	0.741
Cell Stress	(±)-alpha-Lipoic Acid	48	-6	19.9	no	no	0.950	0.997
Cell Stress	(±)-Taxifolin	47	2	21.8	no	no	0.467	0.995

Cell Stress	2,3-Dimethoxy-1,4-naphthoquinone	60	-15	18.2	no	no	0.034	0.292
Cell Stress	3-Nitropropionic acid	56	-9	19.3	no	no	0.230	0.787
Cell Stress	5,5-Dimethyl-1-pyrroline-N-oxide	37	7	22.9	no	no	0.505	0.996
Cell Stress	Allopurinol	40	-10	19.8	no	no	0.134	0.642
Cell Stress	Amifostine	44	-4	21.9	no	no	0.704	0.997
Cell Stress	Astaxanthin	29	17	25.6	no	no*	0.234	0.787
Cell Stress	Caffeic Acid	38	5	22.7	no	no	0.742	0.997
Cell Stress	Hypotaurine	44	1	20.0	no	no	0.854	0.997
Cell Stress	Isoxanthopterin	33	0	20.8	no	no	0.945	0.997
Cell Stress	L-Canavanine	26	5	21.2	no	no	0.837	0.997
Cell Stress	Lonidamine	47	-3	20.0	no	no	0.932	0.997
Cell Stress	Morin	36	10	22.7	no	no	0.267	0.833
Cell Stress	N,N,N',N'-Tetramethylazodicarboxamide	40	1	21.6	no	no	0.588	0.997
Cell Stress	Rotenone	31	-43	11.8	no	no	0.000	0.000
Cell Stress	U-74389G maleate	52	6	22.1	no	no	0.390	0.950
Cholecystokinin	CR 2945	41	-11	18.5	no	no	0.214	0.776
Cholecystokinin	Lorglumide sodium	32	-6	19.3	no	no	0.347	0.923
Cholecystokinin	Proglumide	52	2	21.7	no	no	0.366	0.937
Cholinergic	(-)-Cotinine	49	1	22.0	no	no	0.484	0.996
Cholinergic	(-)-Eseroline fumarate	39	4	21.0	no	no	0.948	0.997
Cholinergic	(-)-Nicotine hydrogen tartrate salt	27	9	23.1	no	no	0.435	0.983
Cholinergic	(-)-Physostigmine	37	11	22.5	no	no	0.232	0.787
Cholinergic	(-)-Scopolamine hydrobromide	37	-11	18.6	no	no	0.243	0.798
Cholinergic	(-)-Scopolamine methyl bromide	48	1	21.0	no	no	0.843	0.997
Cholinergic	(-)-Scopolamine methyl nitrate	38	3	21.6	no	no	0.858	0.997
Cholinergic	(-)-Scopolamine,n-Butyl-, bromide	37	-1	20.7	no	no	0.736	0.997
Cholinergic	(+)-cis-Dioxolane iodide	25	5	22.4	no	no	0.821	0.997
Cholinergic	(+)-Nicotine (+)-di-p-toluoyl tartrate	40	1	21.4	no	no	0.728	0.997
Cholinergic	(+)-Pilocarpine hydrochloride	39	0	21.2	no	no	0.356	0.933
Cholinergic	(±)-Muscarine chloride	38	-6	19.8	no	no	0.327	0.904
Cholinergic	(±)-Vesamicol hydrochloride	36	16	24.3	yes	no	0.047	0.369
Cholinergic	1,1-Dimethyl-4-phenyl-piperazinium iodide	46	0	21.3	no	no	0.826	0.997
Cholinergic	4-DAMP methiodide	40	0	20.9	no	no	0.926	0.997
Cholinergic	4-Diphenylacetoxy-N-(2-chloroethyl)piperidine hydrochloride	26	17	24.5	no	no	0.236	0.787
Cholinergic	9-Amino-1,2,3,4-tetrahydroacridine hydrochloride	34	-28	16.4	no	no	0.000	0.004
Cholinergic	Acetyl-beta-methylcholine chloride	43	-12	20.2	no	no	0.075	0.477
Cholinergic	Acetylcholine mustard hydrochloride	47	11	23.6	yes	no	0.051	0.391
Cholinergic	Acetylthiocholine chloride	34	3	23.4	no	no	0.974	0.997
Cholinergic	AF267B	44	-4	20.0	no	no	0.673	0.997
Cholinergic	alpha-Lobeline hydrochloride	45	-10	18.7	no	no	0.072	0.467
Cholinergic	Aminobenztropine	38	-5	20.7	no	no	0.594	0.997
Cholinergic	Arecaidine propargyl ester hydrobromide	37	6	23.2	no	no	0.934	0.997
Cholinergic	Arecoline hydrobromide	36	10	25.0	no	no	0.575	0.997
Cholinergic	Atropine methyl bromide	43	-6	21.4	no	no	0.519	0.997

Cholinergic	Atropine methyl nitrate	43	0	22.7	no	no	0.788	0.997
Cholinergic	Atropine sulfate	28	-1	22.5	no	no	0.822	0.997
Cholinergic	Benztropine mesylate	46	-1	21.5	no	no	0.759	0.997
Cholinergic	Betaine aldehyde chloride	47	-4	20.8	no	no	0.599	0.997
Cholinergic	Bethanechol chloride	48	7	23.2	no	no	0.367	0.937
Cholinergic	Bromoacetylcholine bromide	43	-14	18.6	no	no	0.107	0.571
Cholinergic	BW 284c51	45	-19	17.8	no	no	0.024	0.235
Cholinergic	Carbachol	45	5	22.7	no	no	0.709	0.997
Cholinergic	Choline bromide	32	2	22.2	no	no	0.866	0.997
Cholinergic	DBO-83	36	5	21.9	no	no	0.687	0.997
Cholinergic	Decamethonium dibromide	46	9	23.3	yes	no	0.096	0.555
Cholinergic	Dicyclomine hydrochloride	46	10	23.1	yes	no	0.036	0.304
Cholinergic	Dihydro-beta-erythroidine hydrobromide	46	13	23.7	yes	no	0.119	0.606
Cholinergic	DL-Homatropine hydrobromide	47	-6	18.7	no	no	0.808	0.997
Cholinergic	Edrophonium chloride	39	-9	18.4	no	no	0.403	0.957
Cholinergic	Gallamine triethiodide	49	-4	19.5	no	no	0.634	0.997
Cholinergic	Hemicholinium-3	39	8	21.5	no	no	0.291	0.862
Cholinergic	Hexahydro-sila-difenidol hydrochloride	43	25	24.9	yes	yes	0.007	0.094
Cholinergic	Hexamethonium bromide	29	26	25.2	yes	no	0.085	0.524
Cholinergic	Hexamethonium dichloride	44	21	24.1	yes	no	0.083	0.511
Cholinergic	Hydroxytacrine maleate	46	4	20.7	no	no	0.471	0.995
Cholinergic	Ipratropium bromide	44	16	23.0	yes	no	0.070	0.462
Cholinergic	Ivermectin	36	-60	8.3	no	no	0.000	0.000
Cholinergic	Karakoline	40	0	20.7	no	no	0.984	0.997
Cholinergic	L-Hyoscyamine	36	1	20.2	no	no	0.564	0.997
Cholinergic	Linopirdine	30	-8	18.8	no	no	0.635	0.997
Cholinergic	McN-A-343	47	-5	20.6	no	no	0.907	0.997
Cholinergic	Mecamylamine hydrochloride	41	4	21.9	no	no	0.829	0.997
Cholinergic	Methoctramine tetrahydrochloride	43	-1	20.9	no	no	0.826	0.997
Cholinergic	Methylcarbamylcholine chloride	53	-10	19.0	no	no	0.234	0.787
Cholinergic	MG 624	43	-24	15.6	no	no	0.002	0.039
Cholinergic	N,N,N-trimethyl-1-(4-trans-stilbenoxy)-2-propylammonium iodide	53	-16	17.7	no	no	0.015	0.174
Cholinergic	Neostigmine bromide	39	-11	19.0	no	no	0.263	0.829
Cholinergic	Orphenadrine hydrochloride	48	-3	20.6	no	no	0.785	0.997
Cholinergic	OXA-22 iodide	54	-5	20.4	no	no	0.588	0.997
Cholinergic	Oxotremorine methiodide	41	-9	19.2	no	no	0.090	0.530
Cholinergic	Oxotremorine sesquifumarate salt	30	6	22.5	no	no	0.996	1.000
Cholinergic	Oxybutynin Chloride	31	5	22.3	no	no	0.503	0.996
Cholinergic	Pancuronium bromide	43	8	22.8	no	no*	0.138	0.657
Cholinergic	Pentolinium di[L(+)-tartrate]	30	2	21.5	no	no	0.555	0.997
Cholinergic	Pilocarpine nitrate	49	-4	20.2	no	no	0.766	0.997
Cholinergic	Pirenzepine dihydrochloride	42	4	21.3	no	no	0.640	0.997
Cholinergic	PNU-282987	36	3	21.8	no	no	0.922	0.997
Cholinergic	Propantheline bromide	45	-3	20.0	no	no	0.420	0.968
Cholinergic	Propofol	24	21	25.4	no	no*	0.271	0.836

Cholinergic	Pyridostigmine bromide	49	-8	18.9	no	no	0.163	0.700
Cholinergic	Succinylcholine chloride	46	-10	18.8	no	no	0.131	0.635
Cholinergic	Telenzepine dihydrochloride	40	-6	19.6	no	no	0.805	0.997
Cholinergic	Tetraethylammonium chloride	36	-4	20.3	no	no	0.351	0.925
Cholinergic	TMPH hydrochloride	43	4	21.4	no	no	0.635	0.997
Cholinergic	Trihexyphenidyl hydrochloride	30	7	22.7	yes	no	0.090	0.530
Cholinergic	Tropicamide	61	3	21.6	no	no	0.371	0.939
Cholinergic	WB 64	36	13	23.5	yes	no	0.061	0.432
Cl- Channel	5-Nitro-2-(3-phenylpropylamino)benzoic acid	37	11	23.4	yes	no	0.046	0.365
Cl- Channel	N-Phenylanthranilic acid	41	-10	20.4	no	no	0.030	0.269
Cl- Channel	R(+)-IAA-94	42	-5	19.6	no	no	0.727	0.997
Cyclic Nucleotides	8-(4-Chlorophenylthio)-cAMP sodium	39	5	22.9	no	no	0.747	0.997
Cyclic Nucleotides	8-Bromo-cAMP sodium	39	-2	21.3	no	no	0.511	0.996
Cyclic Nucleotides	8-Bromo-cGMP sodium	41	-3	21.2	no	no	0.554	0.997
Cyclic Nucleotides	8-Methoxymethyl-3-isobutyl-1-methylxanthine	39	7	22.1	no	no	0.382	0.946
Cyclic Nucleotides	9-cyclopentyladenine	43	7	23.4	no	no	0.973	0.997
Cyclic Nucleotides	BRL 50481	27	26	25.1	yes	yes	0.036	0.304
Cyclic Nucleotides	BRL 50481	46	13	24.5	yes	yes**	0.081	0.502
Cyclic Nucleotides	Cilostamide	26	10	23.8	no	no	0.479	0.996
Cyclic Nucleotides	Cilostazol	53	9	23.7	no	no	0.172	0.717
Cyclic Nucleotides	Enoximone	29	-10	18.8	no	no	0.585	0.997
Cyclic Nucleotides	Forskolin	42	-8	18.7	no	no	0.307	0.885
Cyclic Nucleotides	Ibudilast	45	2	20.4	no	no	1.000	1.000
Cyclic Nucleotides	Imazodan	43	-2	19.5	no	no	0.975	0.997
Cyclic Nucleotides	Isoliquiritigenin	34	9	22.6	yes	no	0.121	0.610
Cyclic Nucleotides	Milrinone	42	12	23.1	yes	no	0.047	0.368
Cyclic Nucleotides	Na-p-Tosyl-L-lysine chloromethyl ketone hydrochloride	34	-13	18.5	no	no	0.855	0.997
Cyclic Nucleotides	NS 2028	42	3	21.7	no	no	0.315	0.897
Cyclic Nucleotides	ODQ	55	3	21.7	no	no	0.277	0.843
Cyclic Nucleotides	Olprinone hydrochloride	63	1	21.0	no	no	0.842	0.997
Cyclic Nucleotides	Papaverine hydrochloride	49	6	22.5	yes	no	0.020	0.207
Cyclic Nucleotides	Pentoxifylline	43	-5	20.0	no	no	0.503	0.996
Cyclic Nucleotides	Protoporphyrin IX disodium	43	-32	13.9	no	no	0.000	0.000
Cyclic Nucleotides	Quazinone	37	13	23.2	no	no	0.155	0.684
Cyclic Nucleotides	Quercetin dihydrate	52	0	20.6	no	no	0.390	0.950
Cyclic Nucleotides	Ro 20-1724	38	4	22.6	no	no	0.217	0.779
Cyclic Nucleotides	Rolipram	47	-6	19.5	no	no	0.377	0.945
Cyclic Nucleotides	SKF 94836	37	-1	20.4	no	no	0.559	0.997
Cyclic Nucleotides	SQ 22536	43	4	22.1	no	no	0.981	0.997
Cyclic Nucleotides	T-0156	50	4	21.7	no	no	0.978	0.997
Cyclic Nucleotides	T-1032	33	3	21.6	no	no	0.835	0.997
Cyclic Nucleotides	Trequinsin hydrochloride	52	14	24.3	yes	yes	0.008	0.107
Cyclic Nucleotides	Vinpocetine	25	17	24.4	yes	yes	0.018	0.187
Cyclic Nucleotides	YC-1	28	-6	19.6	no	no	0.519	0.997
Cyclic Nucleotides	Zaprinast	54	-3	20.2	no	no	0.794	0.997

Cyclic Nucleotides	Zardaverine	27	7	22.3	yes	no	0.124	0.619
Cytoskeleton and ECM	(±)-Thalidomide	27	6	22.0	no	no	0.862	0.997
Cytoskeleton and ECM	2,3-Butanedione	28	3	22.0	no	no	0.920	0.997
Cytoskeleton and ECM	Brefeldin A from Penicillium brefeldianum	41	-2	21.2	no	no	0.938	0.997
Cytoskeleton and ECM	Clodronic acid	47	6	22.8	no	no	0.390	0.950
Cytoskeleton and ECM	Colchicine	31	-7	20.3	no	no	0.561	0.997
Cytoskeleton and ECM	Nocodazole	48	-31	14.2	no	no	0.000	0.000
Cytoskeleton and ECM	Podophyllotoxin	30	6	22.4	no	no	0.726	0.997
Cytoskeleton and ECM	Taxol	33	-40	12.5	no	no	0.000	0.000
Cytoskeleton and ECM	Vinblastine sulfate salt	28	6	22.1	no	no	0.975	0.997
Cytoskeleton and ECM	Vincristine sulfate	33	19	24.8	yes	yes	0.033	0.285
DNA	Carboplatin	31	0	21.7	no	no	0.662	0.997
DNA	Carmustine	48	-11	19.2	no	no	0.336	0.910
DNA	CB 1954	50	-11	19.2	no	no	0.196	0.754
DNA	Chlorambucil	41	11	24.1	no	no	0.224	0.787
DNA	Chloroquine diphosphate	47	-2	21.3	no	no	0.953	0.997
DNA	Cisplatin	40	-5	20.1	no	no	0.973	0.997
DNA	Cyclophosphamide monohydrate	50	0	21.6	no	no	0.841	0.997
DNA	Nimustine hydrochloride	40	-2	20.7	no	no	0.654	0.997
DNA	Phosphonoacetic acid	41	-5	19.5	no	no	0.346	0.923
DNA Metabolism	5-azacytidine	53	-14	19.7	no	no	0.120	0.607
DNA Metabolism	5-Bromo-2'-deoxyuridine	41	0	21.7	no	no	0.933	0.997
DNA Metabolism	5-fluoro-5'-deoxyuridine	35	7	21.7	no	no	0.588	0.997
DNA Metabolism	Altretamine	52	-14	18.8	no	no	0.111	0.582
DNA Metabolism	Ancitabine hydrochloride	34	9	23.9	no	no	0.654	0.997
DNA Metabolism	Azelaic acid	52	-7	21.2	no	no	0.165	0.705
DNA Metabolism	Cytosine-1-beta-D-arabinofuranoside hydrochloride	34	8	23.5	no	no	0.214	0.776
DNA Metabolism	Hydroxyurea	33	-1	19.8	no	no	0.960	0.997
DNA Metabolism	Idarubicin	52	14	22.8	no	no	0.448	0.985
DNA Metabolism	Melphalan	41	-6	19.3	no	no	0.438	0.985
DNA Metabolism	Methotrexate	42	-6	21.4	no	no	0.500	0.996
DNA Metabolism	Mitoxantrone	31	16	23.8	no	no*	0.160	0.697
DNA Metabolism	Mizoribine	43	-1	20.4	no	no	0.467	0.995
DNA Repair	Amsacrine hydrochloride	46	6	23.2	no	no	0.137	0.654
DNA Repair	O6-benzylguanine	50	-9	19.7	no	no	0.197	0.754
DNA Repair	p-Benzoquinone	33	13	24.6	no	no	0.405	0.959
Dopamine	(-)-Quinpirole hydrochloride	58	2	21.1	no	no	0.750	0.997
Dopamine	(-)-Sulpiride	42	2	21.4	no	no	0.723	0.997
Dopamine	(+)-Bromocriptine methanesulfonate	30	15	24.9	no	no*	0.460	0.993
Dopamine	(±)-6-Chloro-PB hydrobromide	33	18	25.0	yes	no	0.022	0.220
Dopamine	(±)-7-Hydroxy-DPAT hydrobromide	51	10	21.9	no	no	0.142	0.661

Dopamine	(±)-Butaclamol hydrochloride	38	-10	19.4	no	no	0.017	0.186
Dopamine	(±)-Chloro-APB hydrobromide	34	-14	18.4	no	no	0.075	0.477
Dopamine	(±)-Octoclothepin maleate	39	33	28.1	yes	yes	0.000	0.000
Dopamine	(±)-PD 128,907 hydrochloride	34	4	21.4	no	no	0.461	0.993
Dopamine	(±)-PPHT hydrochloride	30	-5	19.5	no	no	0.791	0.997
Dopamine	(±)-Quinpirole dihydrochloride	40	0	20.6	no	no	0.813	0.997
Dopamine	(±)-SKF 38393, N-allyl-, hydrobromide	39	5	22.2	no	no	0.926	0.997
Dopamine	(±)-SKF-38393 hydrochloride	48	5	22.1	no	no	0.197	0.754
Dopamine	(±)-Sulpiride	34	-3	20.3	no	no	0.784	0.997
Dopamine	1-(4-Hydroxybenzyl)imidazole-2-thiol	38	13	22.5	no	no	0.174	0.717
Dopamine	1-Phenyl-3-(2-thiazolyl)-2-thiourea	32	-21	16.7	no	no	0.015	0.172
Dopamine	3,4-Dihydroxyphenylacetic acid	46	-1	20.7	no	no	0.821	0.997
Dopamine	3-Methoxy-4-hydroxyphenethylamine hydrochloride	38	-1	20.5	no	no	0.901	0.997
Dopamine	3-Phenylpropargylamine hydrochloride	40	-1	20.4	no	no	0.539	0.997
Dopamine	4-Hydroxy-3-methoxyphenylacetic acid	36	9	21.6	no	no	0.400	0.957
Dopamine	4-Hydroxyphenethylamine hydrochloride	36	2	21.8	no	no	0.883	0.997
Dopamine	4-Methoxy-3-hydroxyphenethylamine hydrochloride	29	14	22.7	no	no	0.385	0.948
Dopamine	6,7-ADTN hydrobromide	37	12	23.4	no	no	0.284	0.857
Dopamine	A-77636 hydrochloride	41	1	22.1	no	no	0.984	0.997
Dopamine	Amantadine hydrochloride	51	-1	22.6	no	no	0.809	0.997
Dopamine	Apomorphine hydrochloride hemihydrate	43	-2	22.3	no	no	0.907	0.997
Dopamine	BP 897	39	3	22.4	yes	no	0.061	0.432
Dopamine	BTCP hydrochloride	51	22	26.4	yes	yes	0.000	0.002
Dopamine	Bupropion hydrochloride	33	7	23.3	no	no	0.224	0.787
Dopamine	Chlorpromazine hydrochloride	33	-34	14.2	no	no	0.000	0.000
Dopamine	Chlorprothixene hydrochloride	43	50	32.5	yes	yes	0.000	0.000
Dopamine	cis(+/-)-8-OH-PBZI hydrobromide	37	1	20.8	no	no	0.640	0.997
Dopamine	cis-(Z)-Flupenthixol dihydrochloride	38	48	30.0	yes	yes	0.000	0.001
Dopamine	Clozapine	39	19	25.9	no	no	0.136	0.650
Dopamine	Cortexolone maleate	47	16	24.3	yes	yes	0.000	0.000
Dopamine	Dihydrexidine hydrochloride	37	12	23.9	no	no	0.482	0.996
Dopamine	Dihydroergocristine methanesulfonate	32	40	29.4	yes	yes	0.000	0.001
Dopamine	Dipropyldopamine hydrobromide	45	-13	18.2	no	no	0.225	0.787
Dopamine	Domperidone	49	6	22.1	no	no	0.142	0.661
Dopamine	Dopamine hydrochloride	46	5	20.9	no	no	0.626	0.997
Dopamine	Droperidol	43	8	23.1	no	no	0.173	0.717
Dopamine	Fenoldopam bromide	42	7	21.6	no	no	0.768	0.997
Dopamine	Fluphenazine dihydrochloride	57	16	23.5	no	no	0.371	0.939
Dopamine	Fluspirilene	49	-44	11.4	no	no	0.000	0.000
Dopamine	Fusaric acid	40	-7	18.8	no	no	0.196	0.754
Dopamine	GBR-12909 dihydrochloride	45	13	23.6	yes	no	0.070	0.462
Dopamine	GBR-12935 dihydrochloride	44	21	24.4	yes	no	0.006	0.088
Dopamine	GYKI 52895	42	7	21.3	no	no	0.299	0.871
Dopamine	Haloperidol	33	-8	18.3	no	no	0.289	0.861

Dopamine	Indatraline hydrochloride	23	-4	19.9	no	no	0.322	0.904
Dopamine	JHW 007 hydrochloride	43	-25	15.7	no	no	0.000	0.002
Dopamine	JL-18	36	6	21.9	no	no	0.204	0.760
Dopamine	L-3,4-Dihydroxyphenylalanine	32	8	22.6	no	no	0.520	0.997
Dopamine	L-3,4-Dihydroxyphenylalanine methyl ester hydrochloride	45	0	21.3	no	no	0.967	0.997
Dopamine	L-741,626	30	-31	14.2	no	no	0.001	0.014
Dopamine	L-745,870 hydrochloride	33	-25	15.4	no	no	0.010	0.128
Dopamine	L-750,667 trihydrochloride	43	-43	11.7	no	no	0.000	0.000
Dopamine	LE 300	30	-6	19.5	no	no	0.657	0.997
Dopamine	Loxapine succinate	41	23	25.4	yes	yes	0.001	0.015
Dopamine	Mesulergine hydrochloride	45	-1	21.1	no	no	0.519	0.997
Dopamine	Methylergonovine maleate	40	26	26.0	yes	yes	0.010	0.128
Dopamine	Metoclopramide hydrochloride	27	21	24.9	yes	no	0.070	0.462
Dopamine	Molindone hydrochloride	40	4	21.6	no	no	0.396	0.953
Dopamine	N-(2-[4-(4-Chlorophenyl)piperazin-1-yl]ethyl)-3-methoxybenzamide	41	29	28.1	yes	yes	0.001	0.012
Dopamine	N-Acetyldopamine monohydrate	38	6	23.1	no	no	0.802	0.997
Dopamine	N-Methyldopamine hydrochloride	33	-2	20.9	no	no	0.954	0.997
Dopamine	Nomifensine maleate	44	-9	19.2	no	no	0.154	0.681
Dopamine	PD 168,077 maleate	40	11	22.8	yes	no	0.023	0.228
Dopamine	Pergolide methanesulfonate	37	32	27.2	yes	yes	0.000	0.000
Dopamine	Perphenazine	31	-8	19.4	no	no	0.123	0.617
Dopamine	Pimozide	54	-32	14.3	no	no	0.000	0.000
Dopamine	Piribedil maleate	35	1	20.8	no	no	0.819	0.997
Dopamine	Prochlorperazine dimaleate	48	2	21.0	no	no	0.403	0.957
Dopamine	Promazine hydrochloride	33	-14	17.7	no	no	0.010	0.132
Dopamine	Propionylpromazine hydrochloride	46	24	25.6	yes	yes	0.000	0.003
Dopamine	Quinelorane dihydrochloride	37	5	21.6	no	no	0.504	0.996
Dopamine	R(-)-2,10,11-Trihydroxyaporphine hybrobromide	50	15	24.1	yes	no	0.005	0.068
Dopamine	R(-)-2,10,11-Trihydroxy-N-propylnoraporphine hydrobromide	36	20	25.1	yes	no	0.062	0.433
Dopamine	R(-)-Apocodeine hydrochloride	27	25	26.2	yes	no	0.017	0.187
Dopamine	R(-)-N-Allylnorapomorphine hydrobromide	38	2	21.5	no	no	0.513	0.996
Dopamine	R(-)-Propylnorapomorphine hydrochloride	54	17	24.6	yes	no	0.002	0.032
Dopamine	R(-)-SCH-12679 maleate	34	-1	21.0	no	no	0.559	0.997
Dopamine	R(+)-3PPP hydrochloride	37	5	21.6	no	no	0.665	0.997
Dopamine	R(+)-6-Bromo-APB hydrobromide	33	6	22.9	no	no	0.808	0.997
Dopamine	R-(+)-7-Hydroxy-DPAT hydrobromide	41	3	20.5	no	no	0.820	0.997
Dopamine	R(+)-SCH-23390 hydrochloride	25	-1	20.7	no	no	0.608	0.997
Dopamine	R(+)-Terguride	38	18	24.7	yes	no	0.025	0.239
Dopamine	Risperidone	39	14	23.8	yes	no	0.100	0.560
Dopamine	Ropinirole hydrochloride	32	2	21.3	no	no	0.775	0.997
Dopamine	S(-)-3PPP hydrochloride	47	3	21.1	no	no	0.901	0.997
Dopamine	S(-)-DS 121 hydrochloride	35	-1	20.8	no	no	0.940	0.997
Dopamine	S-(-)-Eticlopride hydrochloride	37	14	23.1	no	no	0.124	0.619
Dopamine	S(+)-PD 128,907 hydrochloride	32	5	21.6	no	no	0.609	0.997

Dopamine	S(+)-Raclopride L-tartrate	41	-4	20.0	no	no	0.652	0.997
Dopamine	SKF 75670 hydrobromide	44	2	21.3	no	no	0.673	0.997
Dopamine	SKF 83565 hydrobromide	36	-2	20.5	no	no	0.511	0.996
Dopamine	SKF 83959 hydrobromide	49	-9	19.0	no	no	0.274	0.842
Dopamine	SKF 89626	44	-7	19.5	no	no	0.817	0.997
Dopamine	Spiperone hydrochloride	23	6	22.2	no	no	0.235	0.787
Dopamine	Thioridazine hydrochloride	42	34	28.1	yes	yes	0.000	0.000
Dopamine	Thiothixene hydrochloride	45	5	22.4	no	no	0.465	0.995
Dopamine	Tiapride hydrochloride	39	-3	20.6	no	no	0.688	0.997
Dopamine	Trifluoperazine dihydrochloride	41	-8	19.1	no	no	0.225	0.787
Dopamine	Trifluperidol hydrochloride	41	-16	17.6	no	no	0.088	0.530
Dopamine	Triflupromazine hydrochloride	39	-34	14.1	no	no	0.000	0.000
Dopamine	U-101958 maleate	43	4	21.8	no	no	0.413	0.966
Dopamine	U-99194A maleate	38	16	24.3	yes	no	0.003	0.044
G protein	(-)-Perillic acid	45	-6	19.2	no	no	0.550	0.997
G protein	Farnesylthiosalicylic acid	31	-17	16.9	no	no	0.172	0.717
G protein	SCH-202676 hydrobromide	37	-6	19.7	no	no	0.351	0.925
GABA	(-)-Bicuculline methbromide, 1(S), 9(R)	39	0	21.6	no	no	0.657	0.997
GABA	(+)-Hydrastine	48	11	22.1	no	no	0.205	0.760
GABA	(±)-Baclofen	38	1	21.9	no	no	0.934	0.997
GABA	(±)-gamma-Vinyl GABA	50	-4	20.1	no	no	0.753	0.997
GABA	(±)-Nipecotic acid	40	1	22.9	no	no	0.594	0.997
GABA	(E)-4-amino-2-butenoic acid	41	7	22.8	no	no	0.385	0.948
GABA	2-Hydroxysaclofen	51	0	22.7	no	no	0.719	0.997
GABA	3-alpha,21-Dihydroxy-5-alpha-pregnan-20-one	38	2	21.6	no	no	0.444	0.985
GABA	3-Amino-1-propanesulfonic acid sodium	39	8	24.6	no	no	0.330	0.904
GABA	3-Aminopropylphosphonic acid	38	0	22.7	no	no	0.694	0.997
GABA	5alpha-Pregnan-3alpha-ol-11,20-dione	33	4	21.9	no	no	0.735	0.997
GABA	5alpha-Pregnan-3alpha-ol-20-one	34	20	24.6	yes	no	0.047	0.368
GABA	5-Aminovaleric acid hydrochloride	43	-12	20.1	no	no	0.097	0.560
GABA	CGP-13501	37	-3	21.1	no	no	0.579	0.997
GABA	CGP-7930	35	5	22.7	no	no	0.303	0.880
GABA	cis-4-Aminocrotonic acid	25	2	22.3	no	no	0.962	0.997
GABA	Dehydroisoandrosterone 3-sulfate sodium	40	8	23.2	no	no	0.234	0.787
GABA	GABA	33	-3	22.1	no	no	0.614	0.997
GABA	Gabaculine hydrochloride	36	5	23.8	no	no	0.984	0.997
GABA	gamma-Acetylinic GABA	41	4	22.8	no	no	0.709	0.997
GABA	Guvacine hydrochloride	46	7	21.2	no	no	0.506	0.996
GABA	Imidazole-4-acetic acid hydrochloride	34	7	21.4	no	no	0.566	0.997
GABA	Isoguvacine hydrochloride	52	1	20.2	no	no	0.990	0.999
GABA	Isonipecotic acid	43	-4	19.8	no	no	0.736	0.997
GABA	Muscimol hydrobromide	40	-2	19.6	no	no	0.874	0.997
GABA	NCS-356	30	-2	20.7	no	no	0.588	0.997
GABA	NCS-382	43	2	21.6	no	no	0.513	0.996
GABA	N-Methyl-beta-carboline-3-carboxamide	39	-6	19.0	no	no	0.528	0.997
GABA	NO-711 hydrochloride	54	4	22.0	no	no	0.571	0.997

GABA	Phaclofen	46	6	21.7	no	no	0.220	0.785
GABA	Picrotoxin	39	-1	20.9	no	no	0.624	0.997
GABA	Piperidine-4-sulphonic acid	41	-2	20.2	no	no	0.995	1.000
GABA	PK 11195	42	5	22.8	no	no	0.229	0.787
GABA	Pregnenolone sulfate sodium	43	5	21.7	yes	previous	0.105	0.563
GABA	SB 205384	39	-11	18.6	no	no	0.129	0.630
GABA	SKF 89976A hydrochloride	43	-9	19.4	no	no	0.196	0.754
GABA	SKF 97541 hydrochloride	49	-1	21.6	no	no	0.898	0.997
GABA	SR-95531	36	-7	19.8	no	no	0.256	0.820
GABA	THIP hydrochloride	33	-3	20.2	no	no	0.615	0.997
GABA	TPMPA	38	-2	20.4	no	no	0.749	0.997
GABA	Xli 093 hydrate	43	6	23.3	no	no	0.617	0.997
Glutamate	(-)-MK-801 hydrogen maleate	44	3	21.8	no	no	0.328	0.904
Glutamate	(+)-MK-801 hydrogen maleate	39	-5	20.1	no	no	0.482	0.996
Glutamate	(+)-Quisqualic acid	44	7	22.1	no	no	0.600	0.997
Glutamate	(±)-2-Amino-3-phosphonopropionic acid	28	10	25.1	no	no	0.259	0.824
Glutamate	(±)-2-Amino-4-phosphonobutyric acid	53	-7	21.2	no	no	0.292	0.864
Glutamate	(±)-2-Amino-5-phosphonopentanoic acid	36	2	23.3	no	no	0.887	0.997
Glutamate	(±)-2-Amino-7-phosphonoheptanoic acid	31	-5	21.7	no	no	0.425	0.973
Glutamate	(±)-alpha-Methyl-4-carboxyphenylglycine	37	12	23.0	no	no	0.394	0.951
Glutamate	(±)-AMPA hydrobromide	38	1	20.1	no	no	0.945	0.997
Glutamate	(±)-cis-Piperidine-2,3-dicarboxylic acid	55	5	21.6	no	no	0.168	0.710
Glutamate	(±)-CPP	37	-8	19.7	no	no	0.615	0.997
Glutamate	(±)-HA-966	44	-10	19.8	no	no	0.259	0.824
Glutamate	(±)-Ibotenic acid	37	7	22.2	no	no	0.189	0.743
Glutamate	(2S,1'S,2'S)-2-(carboxycyclopropyl)glycine	46	3	22.0	no	no	0.802	0.997
Glutamate	(S)-3,5-Dihydroxyphenylglycine	45	-6	20.2	no	no	0.911	0.997
Glutamate	(S)-MAP4 hydrochloride	33	2	20.9	no	no	0.905	0.997
Glutamate	1,10-Diaminodecane	45	-6	19.6	no	no	0.665	0.997
Glutamate	1-Aminocyclopropanecarboxylic acid hydrochloride	48	-2	22.3	no	no	0.770	0.997
Glutamate	2,6-Difluoro-4-[2-(phenylsulfonylamino)ethylthio]phenoxyacetamide	48	2	21.0	no	no	0.472	0.995
Glutamate	3-Methoxy-morphanin hydrochloride	31	1	21.5	no	no	0.728	0.997
Glutamate	5,7-Dichlorokynurenic acid	42	3	21.6	no	no	0.830	0.997
Glutamate	5-Fluoroindole-2-carboxylic acid	46	3	23.4	no	no	0.818	0.997
Glutamate	6,7-Dichloroquinoxaline-2,3-dione	34	1	21.2	no	no	0.947	0.997
Glutamate	6-Methyl-2-(phenylethynyl)pyridine hydrochloride	41	-14	17.8	no	no	0.035	0.297
Glutamate	7-Chlorokynurenic acid	36	-3	20.7	no	no	0.613	0.997
Glutamate	AIDA	41	-2	21.4	no	no	0.665	0.997
Glutamate	AMN082	43	16	24.7	yes	yes	0.000	0.006
Glutamate	Aniracetam	42	0	21.8	no	no	0.893	0.997
Glutamate	Arcaine sulfate	44	-2	22.3	no	no	0.973	0.997
Glutamate	ATPA	39	-5	20.7	no	no	0.212	0.776
Glutamate	ATPO	44	-4	21.0	no	no	0.671	0.997

Glutamate	Chelidamic acid	47	5	22.9	no	no	0.481	0.996
Glutamate	cis-Azetidine-2,4-dicarboxylic acid	35	-1	21.6	no	no	0.711	0.997
Glutamate	CNQX disodium	33	0	21.4	no	no	0.346	0.923
Glutamate	CNS-1102	43	7	23.3	yes	no	0.099	0.560
Glutamate	CPCCOEt	31	7	23.3	no	no	0.368	0.937
Glutamate	CR 2249	41	1	21.4	no	no	0.635	0.997
Glutamate	CX 546	43	-1	21.3	no	no	0.819	0.997
Glutamate	Cyclothiazide	35	14	24.4	no	no	0.196	0.754
Glutamate	Cystamine dihydrochloride	50	-5	20.7	no	no	0.555	0.997
Glutamate	D(-)-2-Amino-7-phosphonoheptanoic acid	33	-3	21.2	no	no	0.495	0.996
Glutamate	D-Cycloserine	37	-4	20.8	no	no	0.829	0.997
Glutamate	Dextromethorphan hydrobromide monohydrate	37	15	24.6	no	no	0.330	0.904
Glutamate	Dextrorphan D-tartrate	50	-3	20.2	no	no	0.723	0.997
Glutamate	DFB	32	-1	21.1	no	no	0.812	0.997
Glutamate	Dihydrokainic acid	47	3	22.0	no	no	0.744	0.997
Glutamate	DL-threo-beta-hydroxyaspartic acid	43	-4	19.1	no	no	0.809	0.997
Glutamate	DNQX	42	4	22.2	no	no	0.572	0.997
Glutamate	D-Serine	36	0	20.8	no	no	0.929	0.997
Glutamate	Eliprodil	43	17	24.5	yes	yes	0.070	0.462
Glutamate	Felbamate	41	2	20.7	no	no	0.879	0.997
Glutamate	Flupirtine maleate	42	5	21.3	no	no	0.444	0.985
Glutamate	gamma-D-Glutamylaminomethylsulfonic acid	36	11	22.0	no	no	0.242	0.797
Glutamate	GYKI 52466 hydrochloride	40	12	22.2	no	no	0.363	0.937
Glutamate	IEM-1460	44	9	21.7	no	no	0.373	0.940
Glutamate	Ifenprodil tartrate	42	3	21.4	no	no	0.511	0.996
Glutamate	Kainic acid	48	-3	20.0	no	no	0.976	0.997
Glutamate	Kynurenic acid	43	-7	19.2	no	no	0.451	0.988
Glutamate	L-2-aminoadipic acid	36	-4	21.1	no	no	0.511	0.996
Glutamate	L-701,324	50	3	21.4	no	no	0.591	0.997
Glutamate	L-Aspartic acid	46	1	22.0	no	no	0.916	0.997
Glutamate	L-Cysteinesulfinic Acid	45	-5	20.7	no	no	0.696	0.997
Glutamate	L-Glutamic acid hydrochloride	47	-5	19.3	no	no	0.437	0.985
Glutamate	L-Glutamic acid, N-phthaloyl-	32	-2	20.7	no	no	0.912	0.997
Glutamate	L-Glutamine	44	-3	19.6	no	no	0.770	0.997
Glutamate	L-Methionine sulfoximine	37	3	21.2	no	no	0.884	0.997
Glutamate	MDL 105,519	44	-4	20.4	no	no	0.570	0.997
Glutamate	MDL 26,630 trihydrochloride	36	5	22.3	no	no	0.291	0.862
Glutamate	Memantine hydrochloride	42	-5	20.1	no	no	0.820	0.997
Glutamate	N-(3,3-Diphenylpropyl)glycinamide	49	-1	20.0	no	no	0.864	0.997
Glutamate	N-Acetyl-L-Cysteine	26	0	21.9	no	no	0.798	0.997
Glutamate	NBQX disodium	41	1	21.4	no	no	0.362	0.937
Glutamate	N-Methyl-D-aspartic acid	48	1	20.7	no	no	0.350	0.925
Glutamate	NS 521 oxalate	34	13	23.9	yes	no	0.013	0.165
Glutamate	O-Phospho-L-serine	40	-8	19.5	no	no	0.089	0.530
Glutamate	Pentamidine isethionate	33	-1	20.9	no	no	0.872	0.997
Glutamate	Phthalamoyl-L-glutamic acid trisodium	42	1	20.8	no	no	0.889	0.997

Glutamate	Piracetam	41	6	22.4	no	no	0.709	0.997
Glutamate	Putrescine dihydrochloride	44	5	21.6	no	no	0.365	0.937
Glutamate	Quinolinic acid	33	14	23.4	no	no	0.238	0.788
Glutamate	Riluzole	39	-22	16.4	no	no	0.001	0.011
Glutamate	Ro 25-6981 hydrochloride	42	-3	20.2	no	no	0.560	0.997
Glutamate	Ro 8-4304	43	-3	20.3	no	no	0.532	0.997
Glutamate	S(-)-Willardiine	38	10	23.0	no	no	0.549	0.997
Glutamate	SIB 1757	46	9	23.1	no	no	0.541	0.997
Glutamate	SIB 1893	37	1	21.4	no	no	0.948	0.997
Glutamate	Spermidine trihydrochloride	48	-3	20.2	no	no	0.965	0.997
Glutamate	Spermine tetrahydrochloride	47	-6	19.6	no	no	0.339	0.915
Glutamate	trans-(±)-ACPD	54	1	22.0	no	no	0.926	0.997
Glutamate	trans-Azetidine-2,4-dicarboxylic acid	36	4	22.8	no	no	0.956	0.997
Glycine	Phenylbenzene-omega-phosphono-alpha-amino acid	34	-4	19.8	no	no	0.711	0.997
Glycine	Taurine	42	0	21.3	no	no	0.997	1.000
Histamine	(+)-Brompheniramine maleate	33	-3	21.0	no	no	0.875	0.997
Histamine	(+)-Chlorpheniramine maleate	28	8	23.5	no	no	0.555	0.997
Histamine	(±)-Brompheniramine maleate	35	4	22.4	no	no	0.612	0.997
Histamine	(±)-Chlorpheniramine maleate	31	2	22.1	no	no	0.893	0.997
Histamine	1-Methylhistamine dihydrochloride	34	-1	20.4	no	no	0.885	0.997
Histamine	3-(1H-Imidazol-4-yl)propyl di(p-fluorophenyl)methyl ether hydrochloride	48	-22	16.1	no	no	0.000	0.001
Histamine	4-Imidazoleacrylic acid	42	-2	20.5	no	no	0.940	0.997
Histamine	4-Imidazolemethanol hydrochloride	33	21	24.0	yes	no	0.119	0.606
Histamine	Cimetidine	40	3	22.4	no	no	0.985	0.997
Histamine	Clemastine fumarate	46	-19	17.5	no	no	0.002	0.035
Histamine	Clemizole hydrochloride	41	10	23.8	no	no	0.544	0.997
Histamine	Diphenhydramine hydrochloride	38	11	23.9	no	no	0.185	0.740
Histamine	Doxylamine succinate	42	4	22.3	no	no	0.703	0.997
Histamine	Famotidine	41	9	22.1	no	no	0.633	0.997
Histamine	Fexofenadine hydrochloride	45	-2	19.8	no	no	0.936	0.997
Histamine	Histamine dihydrochloride	58	2	20.3	no	no	0.509	0.996
Histamine	Histamine, R(-)-alpha-methyl-, dihydrochloride	34	9	21.8	no	no	0.626	0.997
Histamine	Imetit dihydrobromide	42	0	20.7	no	no	0.644	0.997
Histamine	Ketotifen fumarate	42	1	21.0	no	no	0.553	0.997
Histamine	L-Histidine hydrochloride	40	-4	19.1	no	no	0.771	0.997
Histamine	Loratadine	29	23	25.4	yes	yes	0.013	0.159
Histamine	Methapyrilene hydrochloride	34	12	23.7	no	no	0.282	0.854
Histamine	Oxatomide	35	31	27.6	yes	yes	0.000	0.001
Histamine	Pheniramine maleate	37	0	20.5	no	no	0.440	0.985
Histamine	Promethazine hydrochloride	34	22	25.7	yes	yes	0.026	0.245
Histamine	Pyrilamine maleate	45	1	21.3	no	no	0.665	0.997
Histamine	Ranitidine hydrochloride	50	-5	19.8	no	no	0.511	0.996
Histamine	SKF 91488 dihydrochloride	34	1	21.4	no	no	0.425	0.973
Histamine	SKF 95282 dimaleate	31	10	22.9	yes	no	0.059	0.426

Histamine	Terfenadine	33	43	29.9	yes	no	0.000	0.000
Histamine	Thioperamide maleate	38	3	21.6	no	no	0.907	0.997
Histamine	Triprolidine hydrochloride	39	2	21.7	no	no	0.684	0.997
Hormone	(R,R)-cis-Diethyl tetrahydro-2,8-chrysenediol	26	13	23.6	yes	yes	0.082	0.505
Hormone	1-(2-Chlorophenyl)-1-(4-chlorophenyl)-2,2-dichloroethane	27	8	23.5	no	no*	0.145	0.661
Hormone	1,3,5-tris(4-hydroxyphenyl)-4-propyl-1H-pyrazole	41	-3	19.3	no	no	0.365	0.937
Hormone	17alpha-hydroxyprogesterone	42	0	19.9	no	no	0.829	0.997
Hormone	2-methoxyestradiol	44	-7	19.1	no	no	0.825	0.997
Hormone	4-Androsten-4-ol-3,17-dione	32	8	24.5	no	no	0.156	0.685
Hormone	AC-93253 iodide	46	-72	5.9	no	no	0.000	0.000
Hormone	Acetohexamide	40	0	21.9	no	no	0.730	0.997
Hormone	Androsterone	36	1	22.1	no	no	0.816	0.997
Hormone	Beclomethasone	37	-4	20.9	no	no	0.567	0.997
Hormone	beta-Estradiol	39	19	24.0	yes	yes	0.070	0.462
Hormone	Betamethasone	40	16	25.0	yes	no	0.108	0.571
Hormone	Budesonide	30	-8	19.9	no	no	0.998	1.000
Hormone	Chlorpropamide	43	6	23.0	no	no	0.472	0.995
Hormone	Cortexolone maleate	50	-7	19.1	no	no	0.443	0.985
Hormone	Corticosterone	27	-2	21.2	no	no	0.697	0.997
Hormone	Cortisone	44	-2	21.3	no	no	0.884	0.997
Hormone	Cortisone 21-acetate	43	-1	21.4	no	no	0.883	0.997
Hormone	Cyproterone acetate	37	26	27.4	yes	yes	0.002	0.027
Hormone	Danazol	49	6	22.1	yes	yes	0.061	0.432
Hormone	Estrone	47	-2	19.9	no	no	0.978	0.997
Hormone	Finasteride	37	-7	21.2	no	no	0.721	0.997
Hormone	Flutamide	49	-19	16.5	no	no	0.079	0.496
Hormone	Fulvestrant	35	-10	19.6	no	no	0.141	0.661
Hormone	Hydrocortisone	45	9	21.8	no	no	0.417	0.968
Hormone	Hydrocortisone 21-hemisuccinate sodium	33	2	20.2	no	no	0.631	0.997
Hormone	Mifepristone	46	-5	20.0	no	no	0.871	0.997
Hormone	Nilutamide	33	-7	19.8	no	no	0.861	0.997
Hormone	PQ401	55	-24	15.8	no	no	0.000	0.000
Hormone	Progesterone	36	-14	18.2	no	no	0.008	0.107
Hormone	Raloxifene hydrochloride	42	13	23.6	yes	no	0.003	0.042
Hormone	Spironolactone	43	-3	20.4	no	no	0.758	0.997
Hormone	Tolazamide	42	3	21.9	no	no	0.781	0.997
Hormone	Tolbutamide	36	6	22.6	no	no	0.432	0.979
Hormone	trans-Dehydroandrosterone	40	5	22.6	no	no	0.609	0.997
Hormone	Triamcinolone	43	3	21.8	no	no	0.407	0.963
Imidazoline	Agmatine sulfate	40	-1	22.5	no	no	0.567	0.997
Imidazoline	Antozoline hydrochloride	34	-1	21.7	no	no	0.534	0.997
Imidazoline	Benazoline oxalate	41	3	22.3	no	no	0.505	0.996
Imidazoline	BU224 hydrochloride	40	-4	20.7	no	no	0.672	0.997
Imidazoline	BU99006	50	9	21.6	no	no	0.285	0.857

Imidazoline	Efaroxan hydrochloride	39	6	21.4	no	no	0.737	0.997
Imidazoline	Harmane	34	21	24.1	yes	no	0.010	0.128
Imidazoline	Idazoxan hydrochloride	43	-5	19.7	no	no	0.455	0.990
Imidazoline	Metrazoline oxalate	42	0	20.6	no	no	0.847	0.997
Imidazoline	Rilmenidine hemifumarate	43	-2	20.5	no	no	0.659	0.997
Immune System	(E)-5-(2-Bromovinyl)-2'-deoxyuridine	42	-10	19.5	no	no	0.061	0.432
Immune System	2',3'-didehydro-3'-deoxythymidine	36	3	22.0	no	no	0.744	0.997
Immune System	2',3'-dideoxycytidine	45	10	23.5	no	no	0.223	0.787
Immune System	3'-Azido-3'-deoxythymidine	31	8	24.5	yes	no	0.112	0.582
Immune System	3-deazaadenosine	48	3	21.5	no	no	0.677	0.997
Immune System	6-Aminohexanoic acid	53	-8	20.0	no	no	0.457	0.990
Immune System	Acyclovir	43	-9	20.8	no	no	0.162	0.700
Immune System	AFMK	48	-7	19.0	no	no	0.145	0.661
Immune System	Amiprilose hydrochloride	40	-6	21.4	no	no	0.171	0.717
Immune System	CV-3988	30	9	22.7	yes	no	0.042	0.341
Immune System	Leflunomide	39	-7	19.2	no	no	0.838	0.997
Immune System	Pirfenidone	42	2	21.6	no	no	0.716	0.997
Intracellular Ca+2	Calcimycin	26	7	23.3	no	no	0.560	0.997
Intracellular Ca+2	Calmidazolium chloride	41	-21	17.2	no	no	0.015	0.174
Intracellular Ca+2	Dantrolene sodium	36	5	22.1	no	no	0.476	0.996
Intracellular Ca+2	Thapsigargin	27	15	23.9	no	no	0.551	0.997
Intracellular Ca+2	Thio-NADP sodium	43	-2	20.8	no	no	0.793	0.997
Intracellular Ca+2	TMB-8 hydrochloride	36	-3	22.1	no	no	0.890	0.997
Intracellular Ca+2	W-7 hydrochloride	39	-15	19.4	no	no	0.221	0.786
Ion Channels	SCH-28080	40	-16	17.5	no	no	0.252	0.817
Ion Pump	3',4'-Dichlorobenzamil	31	-25	15.7	no	no	0.228	0.787
Ion Pump	5-(N,N-Dimethyl)amiloride hydrochloride	45	-11	20.2	no	no	0.147	0.665
Ion Pump	5-(N,N-hexamethylene)amiloride	32	3	22.5	no	no	0.273	0.841
Ion Pump	5-(N-Ethyl-N-isopropyl)amiloride	37	-7	21.1	no	no	0.323	0.904
Ion Pump	5-(N-Methyl-N-isobutyl)amiloride	27	-4	21.9	no	no	0.957	0.997
Ion Pump	Benzamil hydrochloride	35	-8	19.9	no	no	0.663	0.997
Ion Pump	Bumetanide	36	-6	20.4	no	no	0.763	0.997
Ion Pump	Dihydroouabain	44	-3	20.7	no	no	0.896	0.997
Ion Pump	Flunarizine dihydrochloride	39	-27	14.7	no	no	0.001	0.012
Ion Pump	Furosemide	41	-2	19.9	no	no	0.926	0.997
Ion Pump	Lansoprazole	44	10	22.8	no	no	0.184	0.737
Ion Pump	Ouabain	40	-1	21.0	no	no	0.433	0.980
Ion Pump	R(+)-Butylindazone	43	-1	20.7	no	no	0.601	0.997
Ion Pump	Ruthenium red	36	-6	19.7	no	no	0.584	0.997
Ion Pump	Sanguinarine chloride	42	-35	13.5	no	no	0.000	0.000
K+ Channel	2,3-Butanedione monoxime	38	8	23.7	no	no	0.562	0.997
K+ Channel	4-Aminopyridine	39	8	24.6	no	no	0.299	0.871
K+ Channel	5-hydroxydecanoic acid sodium	40	0	19.9	no	no	0.891	0.997
K+ Channel	Alinidine	39	6	21.5	no	no	0.906	0.997
K+ Channel	CBIQ	35	-77	5.0	no	no	0.000	0.000
K+ Channel	Cibenzoline succinate	31	5	21.7	no	no	0.333	0.908

K+ Channel	Clotrimazole	42	-32	14.7	no	no	0.003	0.044
K+ Channel	DCEBIO	52	-4	20.1	no	no	0.423	0.972
K+ Channel	Dequalinium dichloride	37	-52	10.4	no	no	0.000	0.000
K+ Channel	Diazoxide	41	11	23.3	yes	no	0.098	0.560
K+ Channel	Disopyramide phosphate	38	-13	18.2	no	no	0.651	0.997
K+ Channel	Glibenclamide	34	4	21.1	no	no	0.793	0.997
K+ Channel	Glipizide	33	10	21.8	no	no	0.758	0.997
K+ Channel	IMID-4F hydrochloride	34	15	22.9	no	no	0.331	0.904
K+ Channel	Minoxidil	43	2	21.1	no	no	0.644	0.997
K+ Channel	NS-1619	47	-16	17.6	no	no	0.025	0.239
K+ Channel	Pinacidil	48	-9	18.7	no	no	0.479	0.996
K+ Channel	PNU-37887A	45	-2	21.5	no	no	0.643	0.997
K+ Channel	Propafenone hydrochloride	44	-7	19.7	no	no	0.530	0.997
K+ Channel	Psora-4	46	26	27.5	yes	yes	0.000	0.002
K+ Channel	Quinidine sulfate	35	18	24.2	yes	yes	0.053	0.394
K+ Channel	Quinine sulfate	38	-5	19.6	no	no	0.662	0.997
K+ Channel	Rutaecarpine	38	-3	20.2	no	no	0.703	0.997
Leukotriene	AA-861	44	-9	20.8	no	no	0.289	0.861
Leukotriene	BWB70C	40	-12	19.1	no	no	0.014	0.168
Leukotriene	Ebselen	37	2	20.7	no	no	0.750	0.997
Leukotriene	Hydroquinone	33	14	22.8	no	no	0.279	0.848
Leukotriene	MK-886	47	4	21.3	no	no	0.783	0.997
Leukotriene	Nordihydroguaiaretic acid from Larrea divaricata (creosote bush)	36	-1	21.0	no	no	0.910	0.997
Leukotriene	REV 5901	44	-31	14.4	no	no	0.000	0.002
Leukotriene	SR 2640	54	-4	20.0	no	no	0.682	0.997
Leukotriene	Tranilast	39	-6	20.0	no	no	0.255	0.820
Leukotriene	U-75302	35	4	21.7	no	no	0.637	0.997
Lipid	(Z)-Gugglesterone	43	9	22.7	no	no	0.166	0.705
Lipid	7,7-Dimethyl-(5Z,8Z)-eicosadienoic acid	32	0	20.9	no	no	0.894	0.997
Lipid	Bromoenol lactone	31	21	26.4	yes	no	0.014	0.168
Lipid	Citicoline sodium	60	10	23.7	no	no*	0.146	0.662
Lipid	Clofibrate	43	-5	20.7	no	no	0.526	0.997
Lipid	D-609 potassium	35	-4	20.1	no	no	0.600	0.997
Lipid	ET-18-OCH3	42	-15	17.9	no	no	0.210	0.773
Lipid	L-165,041	32	21	24.1	no	no*	0.254	0.820
Lipid	Ranolazine dihydrochloride	53	-7	19.3	no	no	0.328	0.904
Lipid	Sandoz 58-035	46	-10	20.5	no	no	0.412	0.966
Lipid	U-73122	48	-1	20.8	no	no	0.978	0.997
Melatonin	6-Hydroxymelatonin	41	-1	19.6	no	no	0.598	0.997
Melatonin	IIK7	28	5	21.6	no	no	0.392	0.951
Melatonin	K 185	39	5	21.6	yes	no	0.028	0.255
Melatonin	Melatonin	50	-6	19.4	no	no	0.151	0.678
Melatonin	N-Acetyl-5-hydroxytryptamine	44	1	23.0	no	no	0.998	1.000
Melatonin	N-Acetyltryptamine	24	-2	21.4	no	no	0.987	0.998

Multi-Drug Resistance	1-(4-Chlorobenzyl)-5-methoxy-2-methylindole-3-acetic acid	38	7	23.2	no	no	0.236	0.787
Multi-Drug Resistance	1-(4-Fluorobenzyl)-5-methoxy-2-methylindole-3-acetic acid	36	-12	17.7	no	no	0.382	0.946
Multi-Drug Resistance	1-Aminobenzotriazole	50	-1	22.5	no	no	0.472	0.995
Multi-Drug Resistance	1-benzoyl-5-methoxy-2-methylindole-3-acetic acid	43	-11	19.4	no	no	0.216	0.776
Multi-Drug Resistance	3-Aminopropionitrile fumarate	30	-8	20.9	no	no	0.227	0.787
Multi-Drug Resistance	DL-Buthionine-[S,R]-sulfoximine	62	-1	21.5	no	no	0.800	0.997
Multi-Drug Resistance	Ketoconazole	44	2	21.2	no	no	0.170	0.715
Multi-Drug Resistance	L-Buthionine-sulfoximine	36	8	23.4	no	no	0.386	0.948
Multi-Drug Resistance	S-(p-Azidophenacyl)glutathione	40	-7	21.2	no	no	0.234	0.787
Multi-Drug Resistance	SKF-525A hydrochloride	27	0	21.1	no	no	0.943	0.997
Multi-Drug Resistance	Sodium Taurocholate	32	0	22.7	no	no	0.919	0.997
Multi-Drug Resistance	Sulfaphenazole	45	-3	20.2	no	no	0.495	0.996
Na+ Channel	Amiloride hydrochloride	45	-16	18.3	no	no	0.043	0.351
Na+ Channel	Disopyramide	45	8	22.6	no	no	0.147	0.664
Na+ Channel	Flecainide acetate	35	4	21.1	no	no	0.902	0.997
Na+ Channel	Lidocaine hydrochloride	32	7	22.3	no	no	0.631	0.997
Na+ Channel	Lidocaine N-ethyl bromide quaternary salt	33	2	21.0	no	no	0.869	0.997
Na+ Channel	Lidocaine N-methyl hydrochloride	34	2	21.1	no	no	0.971	0.997
Na+ Channel	Metolazone	88	6	21.2	no	no	0.330	0.904
Na+ Channel	Mexiletene hydrochloride	41	-5	19.5	no	no	0.535	0.997
Na+ Channel	N-Acetylprocainamide hydrochloride	23	2	23.3	no	no	0.744	0.997
Na+ Channel	N-Bromoacetamide	41	-3	21.0	no	no	0.972	0.997
Na+ Channel	Phenamil methanesulfonate	47	-12	18.1	no	no	0.173	0.717
Na+ Channel	Prilocaine hydrochloride	31	8	22.2	no	no	0.244	0.801
Na+ Channel	Procainamide hydrochloride	42	3	21.2	no	no	0.895	0.997
Na+ Channel	Procaine hydrochloride	32	8	22.2	no	no	0.497	0.996
Na+ Channel	R(-)-Me5	38	8	22.2	no	no	0.320	0.902
Na+ Channel	Tetracaine hydrochloride	48	-2	20.5	no	no	0.922	0.997
Na+ Channel	Tocainide hydrochloride	41	9	23.4	no	no	0.144	0.661
Na+ Channel	Triamterene	41	-2	20.8	no	no	0.372	0.940
Neurodegeneration	BF-170 hydrochloride	48	-21	16.2	no	no	0.000	0.007
Neurodegeneration	Ro 90-7501	36	18	24.5	yes	no	0.005	0.075
Neurotransmission	(-)-cis-(1S,2R)-U-50488 tartrate	36	9	22.4	no	no	0.365	0.937
Neurotransmission	(±)-2,3-Dichloro-alpha-methylbenzylamine hydrochloride	33	3	21.6	no	no	0.522	0.997
Neurotransmission	(±)-3-(3,4-dihydroxyphenyl)-2-methyl-DL-alanine	30	13	23.3	no	no	0.202	0.758
Neurotransmission	(±)-p-Chlorophenylalanine	36	12	24.3	no	no	0.522	0.997
Neurotransmission	(6R)-5,6,7,8-Tetrahydro-L-biopterin hydrochloride	36	4	22.2	no	no	0.418	0.968
Neurotransmission	1-Amino-1-cyclohexanecarboxylic acid hydrochloride	50	-3	21.1	no	no	0.794	0.997

Neurotransmission	2-Cyclooctyl-2-hydroxyethylamine hydrochloride	36	-2	21.0	no	no	0.943	0.997
Neurotransmission	3,5-Dinitrocatechol	36	-1	20.8	no	no	0.573	0.997
Neurotransmission	3-Iodo-L-tyrosine	41	0	20.6	no	no	0.820	0.997
Neurotransmission	6-Methoxy-1,2,3,4-tetrahydro-9H-pyrido[3,4b] indole	40	6	24.3	no	no	0.529	0.997
Neurotransmission	alpha-Methyl-DL-tyrosine methyl ester hydrochloride	44	2	20.9	no	no	0.800	0.997
Neurotransmission	BRL 52537 hydrochloride	40	3	21.8	no	no	0.303	0.880
Neurotransmission	Captopril	40	3	22.4	no	no	0.939	0.997
Neurotransmission	Carisoprodol	28	11	24.2	no	no	0.447	0.985
Neurotransmission	Chlormezanone	32	-5	20.4	no	no	0.401	0.957
Neurotransmission	Clorgyline hydrochloride	32	-2	20.1	no	no	0.485	0.996
Neurotransmission	Debrisoquin sulfate	35	-4	20.6	no	no	0.798	0.997
Neurotransmission	DL-alpha-Methyl-p-tyrosine	45	-14	19.6	no	no	0.135	0.648
Neurotransmission	DL-p-Chlorophenylalanine methyl ester hydrochloride	38	1	22.1	no	no	0.814	0.997
Neurotransmission	DL-Thiorphan	35	8	22.9	no	no	0.329	0.904
Neurotransmission	Hydralazine hydrochloride	49	15	22.9	no	no	0.288	0.861
Neurotransmission	Hydroxylamine hydrochloride	40	9	21.7	no	no	0.380	0.946
Neurotransmission	Icilin	38	-5	20.0	no	no	0.581	0.997
Neurotransmission	Iofetamine hydrochloride	44	10	22.8	no	no	0.363	0.937
Neurotransmission	Iproniazid phosphate	39	-4	19.9	no	no	0.603	0.997
Neurotransmission	L-162,313	37	13	23.3	yes	no	0.021	0.212
Neurotransmission	L-368,899	47	-1	20.5	no	no	0.792	0.997
Neurotransmission	L-alpha-Methyl-p-tyrosine	49	-1	21.0	no	no	0.230	0.787
Neurotransmission	Lithium Chloride	32	5	21.0	no	no	0.766	0.997
Neurotransmission	NBI 27914	35	23	24.9	yes	no	0.048	0.374
Neurotransmission	Nialamide	47	7	22.6	no	no	0.411	0.966
Neurotransmission	N-Oleoyldopamine	49	-3	20.3	no	no	0.900	0.997
Neurotransmission	N-Succinyl-L-proline	44	0	20.9	no	no	0.859	0.997
Neurotransmission	Org 24598 lithium salt	45	-3	22.0	no	no	0.932	0.997
Neurotransmission	Pargyline hydrochloride	42	-5	19.6	no	no	0.360	0.937
Neurotransmission	Pentylenetetrazole	56	-6	19.8	no	no	0.245	0.801
Neurotransmission	p-Fluoro-L-phenylalanine	40	0	20.3	no	no	0.906	0.997
Neurotransmission	Phenelzine sulfate	28	7	22.1	no	no	0.492	0.996
Neurotransmission	Quinacrine dihydrochloride	43	-8	18.9	no	no	0.993	1.000
Neurotransmission	R-(-)-Deprenyl hydrochloride	38	3	21.8	no	no	0.326	0.904
Neurotransmission	R-(-)-Desmethyldeprenyl hydrochloride	38	9	22.9	no	no	0.216	0.776
Neurotransmission	Ro 16-6491 hydrochloride	28	-2	20.4	no	no	0.709	0.997
Neurotransmission	Ro 41-0960	45	3	21.5	no	no	0.237	0.787
Neurotransmission	Ro 41-1049 hydrochloride	39	-8	19.3	no	no	0.386	0.948
Neurotransmission	S-(+)-PD 123177 trifluoroacetate salt hydrate	30	2	22.0	no	no	0.994	1.000
Neurotransmission	SB 218795	35	-3	19.4	no	no	0.688	0.997
Neurotransmission	Semicarbazide hydrochloride	34	3	21.6	no	no	0.942	0.997
Neurotransmission	Tranylcypromine hydrochloride	50	-2	20.1	no	no	0.779	0.997
Nitric Oxide	(±)-AMT hydrochloride	46	1	22.0	no	no	0.717	0.997

Nitric Oxide	1,3-PBIT dihydrobromide	33	5	21.5	no	no	0.398	0.955
Nitric Oxide	1,4-PBIT dihydrobromide	33	-9	18.7	no	no	0.753	0.997
Nitric Oxide	1-[2-(Trifluoromethyl)phenyl]imidazole	47	2	21.7	no	no	0.750	0.997
Nitric Oxide	1400W dihydrochloride	38	5	21.9	no	no	0.945	0.997
Nitric Oxide	2-(2-Aminoethyl)isothiourea dihydrobromide	38	-6	21.4	no	no	0.609	0.997
Nitric Oxide	3-Bromo-7-nitroindazole	35	-5	20.6	no	no	0.617	0.997
Nitric Oxide	3-Morpholinosydnonimine hydrochloride	50	3	21.9	no	no	0.580	0.997
Nitric Oxide	4-Phenyl-3-furoxancarbonitrile	34	24	26.3	yes	yes	0.005	0.068
Nitric Oxide	7-Nitroindazole	32	-1	21.0	no	no	0.787	0.997
Nitric Oxide	alpha-Guanidinoglutaric acid	40	2	20.7	no	no	0.930	0.997
Nitric Oxide	Aminoguanidine hemisulfate	36	4	23.7	no	no	0.711	0.997
Nitric Oxide	Aminoguanidine hydrochloride	35	2	22.3	no	no	0.708	0.997
Nitric Oxide	Ammonium pyrrolidinedithiocarbamate	50	-19	16.6	no	no	0.001	0.020
Nitric Oxide	Chlorzoxazone	42	-10	19.5	no	no	0.068	0.462
Nitric Oxide	Diphenyleneiodonium chloride	47	-46	11.5	no	no	0.000	0.000
Nitric Oxide	L-Arginine	55	1	22.9	no	no	0.308	0.885
Nitric Oxide	L-Canavanine sulfate	33	6	23.1	no	no	0.510	0.996
Nitric Oxide	L-N6-(1-Iminoethyl)lysine hydrochloride	33	7	22.2	no	no	0.588	0.997
Nitric Oxide	Molsidomine	43	1	20.9	no	no	0.542	0.997
Nitric Oxide	N^G,N^G-Dimethylarginine hydrochloride	33	7	23.0	no	no	0.219	0.785
Nitric Oxide	NADPH tetrasodium	52	-5	20.1	no	no	0.965	0.997
Nitric Oxide	NG-Hydroxy-L-arginine acetate	37	18	23.5	no	no	0.193	0.754
Nitric Oxide	NG-Monomethyl-L-arginine acetate	38	1	20.7	no	no	0.536	0.997
Nitric Oxide	NG-Nitro-L-arginine	36	-4	20.3	no	no	0.732	0.997
Nitric Oxide	NG-Nitro-L-arginine methyl ester hydrochloride	38	-2	20.7	no	no	0.417	0.968
Nitric Oxide	Sepiapterin	44	-1	21.0	no	no	0.730	0.997
Nitric Oxide	S-Ethylisothiourea hydrobromide	41	-13	17.5	no	no	0.126	0.624
Nitric Oxide	S-Methylisothiourea hemisulfate	48	-10	18.4	no	no	0.030	0.269
Nitric Oxide	S-Methyl-L-thiocitrulline acetate	53	-4	19.8	no	no	0.583	0.997
Nitric Oxide	S-Nitrosoglutathione	31	-6	19.9	no	no	0.504	0.996
Nitric Oxide	S-Nitroso-N-acetylpenicillamine	35	8	22.9	no	no	0.449	0.987
Nitric Oxide	Sodium nitroprusside dihydrate	37	-13	18.2	no	no	0.014	0.169
Nitric Oxide	Thiocitrulline	42	1	21.1	no	no	0.585	0.997
Nootropic	Centrophenoxine hydrochloride	31	10	24.0	no	no	0.331	0.904
Nootropic	DM 235	38	3	22.1	no	no	0.631	0.997
Nootropic	Oxiracetam	40	2	21.6	no	no	0.764	0.997
Opioid	(-)-3-Methoxynaltrexone hydrochloride	41	-10	19.0	no	no	0.103	0.560
Opioid	(-)-trans-(1S,2S)-U-50488 hydrochloride	40	1	21.2	no	no	0.660	0.997
Opioid	(+)-Cyclazocine	58	-5	20.4	no	no	0.838	0.997
Opioid	(+)-N-Allylnormetazocine hydrochloride	37	0	21.8	no	no	0.550	0.997
Opioid	(±) trans-U-50488 methanesulfonate	35	2	21.4	no	no	0.709	0.997
Opioid	(±)-N-Allylnormetazocine hydrochloride	44	-3	21.3	no	no	0.552	0.997
Opioid	AC 915 oxalate	45	6	24.1	no	no	0.227	0.787
Opioid	Carbetapentane citrate	40	-4	20.9	no	no	0.665	0.997
Opioid	GR-89696 fumarate	56	-4	19.1	no	no	0.577	0.997
Opioid	Guanidinyl-naltrindole di-trifluoroacetate	55	-7	18.8	no	no	0.594	0.997

Opioid	ICI 204,448 hydrochloride	35	-4	19.8	no	no	0.628	0.997
Opioid	L-687,384 hydrochloride	43	-10	18.6	no	no	0.193	0.754
Opioid	Levallorphan tartrate	30	7	22.0	no	no	0.490	0.996
Opioid	Loperamide hydrochloride	33	8	22.3	no	no	0.312	0.894
Opioid	Nalbuphine hydrochloride	35	0	21.2	no	no	0.951	0.997
Opioid	Naloxonazine dihydrochloride	36	-12	18.5	no	no	0.024	0.235
Opioid	Naloxone benzoylhydrazone	42	8	22.8	no	no	0.177	0.725
Opioid	Naloxone hydrochloride	29	4	22.1	no	no	0.935	0.997
Opioid	Naltrexone hydrochloride	53	-6	19.9	no	no	0.453	0.990
Opioid	Naltriben methanesulfonate	51	-2	20.8	no	no	0.445	0.985
Opioid	Naltrindole hydrochloride	34	-4	20.4	no	no	0.535	0.997
Opioid	nor-Binaltorphimine dihydrochloride	32	3	21.8	no	no	0.775	0.997
Opioid	Noscapine hydrchloride	51	-13	18.3	no	no	0.430	0.977
Opioid	PRE-084	33	3	21.7	no	no	0.615	0.997
Opioid	SNC80	42	2	21.2	no	no	0.394	0.951
Opioid	U-62066	42	2	21.2	no	no	0.841	0.997
Opioid	U-69593	40	-13	18.2	no	no	0.179	0.726
P2 Receptor	2-Chloroadenosine triphosphate tetrasodium	45	3	22.0	no	no	0.353	0.930
P2 Receptor	2-Methylthioadenosine diphosphate trisodium	42	4	21.9	no	no	0.493	0.996
P2 Receptor	2-Methylthioadenosine triphosphate tetrasodium	36	1	22.2	no	no	0.675	0.997
P2 Receptor	alpha,beta-Methylene adenosine 5'-triphosphate dilithium	44	4	21.3	no	no	0.513	0.996
P2 Receptor	ARL 67156 trisodium salt	32	8	23.5	no	no	0.744	0.997
P2 Receptor	Azathioprine	38	-1	22.5	no	no	0.984	0.997
P2 Receptor	MRS 2159	40	6	21.8	no	no	0.566	0.997
P2 Receptor	MRS 2179	53	-7	19.2	no	no	0.168	0.710
P2 Receptor	NF 023	46	-7	19.8	no	no	0.668	0.997
P2 Receptor	PPADS	42	-1	20.3	no	no	0.624	0.997
P2 Receptor	PPNDS tetrasodium	34	6	22.3	no	no	0.306	0.885
P2 Receptor	Reactive Blue 2	51	1	21.1	no	no	0.961	0.997
P2 Receptor	Suramin hexasodium	31	-1	21.0	no	no	0.804	0.997
P2 Receptor	Uridine 5'-diphosphate sodium	40	-6	19.5	no	no	0.154	0.681
Phosphorylation	(-)-Tetramisole hydrochloride	45	21	25.1	yes	no	0.000	0.006
Phosphorylation	1-(5-Isoquinolinylsulfonyl)-2-methylpiperazine dihydrochloride	37	2	21.2	no	no	0.948	0.997
Phosphorylation	1-(5-Isoquinolinylsulfonyl)-3-methylpiperazine dihydrochloride	44	3	21.2	no	no	0.510	0.996
Phosphorylation	1,4-Dideoxy-1,4-imino-D-arabinitol	39	2	21.8	no	no	0.565	0.997
Phosphorylation	2,6-Diamino-4-pyrimidinone	39	-11	18.7	no	no	0.355	0.933
Phosphorylation	7-Cyclopentyl-5-(4-phenoxy)phenyl-7H-pyrrolo[2,3-d]pyrimidin-4-ylamine	25	14	23.8	yes	yes	0.044	0.351
Phosphorylation	A3 hydrochloride	41	-2	20.2	no	no	0.408	0.964
Phosphorylation	Adenosine 3',5'-cyclic monophosphate	54	-10	19.7	no	no	0.258	0.824
Phosphorylation	Aurothioglucose	41	0	20.7	no	no	0.561	0.997
Phosphorylation	BIO	41	-43	11.8	no	no	0.000	0.000
Phosphorylation	Cantharidic Acid	28	-29	15.4	no	no	0.000	0.001
Phosphorylation	Cantharidin	46	-40	13.1	no	no	0.000	0.000

Phosphorylation	Ceramide	37	-1	21.4	no	no	0.956	0.997
Phosphorylation	CGP 57380	31	-7	19.4	no	no	0.846	0.997
Phosphorylation	CGP-74514A hydrochloride	34	-31	15.0	no	no	0.000	0.000
Phosphorylation	Chelerythrine chloride	32	-19	17.6	no	no	0.068	0.462
Phosphorylation	CK2 Inhibitor 2	40	-1	21.2	no	no	0.547	0.997
Phosphorylation	Cyclosporin A	41	14	24.9	yes	yes	0.015	0.174
Phosphorylation	DAPH	47	14	24.8	yes	yes	0.024	0.235
Phosphorylation	Daphnetin	39	6	22.6	no	no	0.660	0.997
Phosphorylation	Dephostatin	38	9	22.7	no	no	0.440	0.985
Phosphorylation	Diacylglycerol Kinase Inhibitor II	47	16	24.8	yes	no	0.028	0.255
Phosphorylation	DL-erythro-Dihydrosphingosine	29	10	23.1	no	no	0.628	0.997
Phosphorylation	DL-Stearoylcarnitine chloride	45	-13	18.2	no	no	0.030	0.269
Phosphorylation	Emodin	36	-7	18.8	no	no	0.037	0.307
Phosphorylation	Endothall	31	10	22.2	no	no	0.359	0.937
Phosphorylation	Furegrelate sodium	39	-2	19.9	no	no	0.938	0.997
Phosphorylation	Genistein	43	6	21.5	no	no	0.595	0.997
Phosphorylation	GW2974	43	7	21.6	no	no	0.296	0.871
Phosphorylation	GW5074	34	2	20.6	no	no	0.782	0.997
Phosphorylation	H-8 dihydrochloride	38	7	22.7	no	no	0.234	0.787
Phosphorylation	H-89	43	-28	15.7	no	no	0.001	0.017
Phosphorylation	H-9 dihydrochloride	46	-4	21.1	no	no	0.707	0.997
Phosphorylation	HA-100	40	-10	18.0	no	no	0.401	0.957
Phosphorylation	HA-1004 hydrochloride	38	-4	19.2	no	no	0.558	0.997
Phosphorylation	Hispidin	26	18	23.4	no	no	0.458	0.990
Phosphorylation	IC 261	29	11	22.8	yes	no	0.059	0.426
Phosphorylation	Indirubin-3'-oxime	31	16	23.1	no	no	0.552	0.997
Phosphorylation	I-OMe-Tyrphostin AG 538	45	10	23.0	yes	no	0.027	0.252
Phosphorylation	Kenpaullone	45	21	25.0	yes	yes	0.001	0.016
Phosphorylation	LFM-A13	35	23	25.4	yes	yes	0.002	0.033
Phosphorylation	LY-294,002 hydrochloride	42	0	20.7	no	no	0.484	0.996
Phosphorylation	Me-3,4-dephostatin	30	-1	20.9	no	no	0.998	1.000
Phosphorylation	ML-7	42	-9	18.9	no	no	0.825	0.997
Phosphorylation	ML-9	44	-7	20.2	no	no	0.921	0.997
Phosphorylation	Myricetin	36	8	22.2	yes	no	0.069	0.462
Phosphorylation	Norcantharidin	37	-5	20.0	no	no	0.967	0.997
Phosphorylation	NSC 95397	38	-11	17.7	no	no	0.073	0.471
Phosphorylation	Oleic Acid	43	-3	20.5	no	no	0.587	0.997
Phosphorylation	Olomoucine	40	5	22.1	no	no	0.798	0.997
Phosphorylation	Palmitoyl-DL-Carnitine chloride	32	13	23.8	yes	no	0.015	0.174
Phosphorylation	PD 169316	36	-21	16.6	no	no	0.067	0.455
Phosphorylation	PD 98,059	36	-1	20.4	no	no	0.521	0.997
Phosphorylation	Phorbol 12-myristate 13-acetate	28	-49	10.4	no	no	0.000	0.000
Phosphorylation	Piceatannol	44	-12	18.6	no	no	0.181	0.733
Phosphorylation	rac-2-Ethoxy-3-hexadecanamido-1-propylphosphocholine	30	-8	18.5	no	no	0.144	0.661

Phosphorylation	rac-2-Ethoxy-3-octadecanamido-1-propylphosphocholine	37	-2	19.9	no	no	0.624	0.997
Phosphorylation	Roscovitine	38	-1	20.7	no	no	0.368	0.937
Phosphorylation	Rottlerin	40	6	22.1	no	no	0.209	0.772
Phosphorylation	S(-)-p-Bromotetramisole oxalate	42	12	25.5	no	no	0.326	0.904
Phosphorylation	SB 202190	37	-3	21.0	no	no	0.765	0.997
Phosphorylation	SB 216763	38	7	22.5	no	no	0.719	0.997
Phosphorylation	SB 415286	42	-2	19.8	no	no	0.980	0.997
Phosphorylation	SD-169	46	-4	20.5	no	no	0.810	0.997
Phosphorylation	SP600125	47	-22	16.4	no	no	0.004	0.059
Phosphorylation	Sphingosine	39	5	22.0	no	no	0.519	0.997
Phosphorylation	SU 4312	29	16	24.6	yes	yes	0.000	0.005
Phosphorylation	SU 5416	38	-13	18.2	no	no	0.055	0.407
Phosphorylation	SU 6656	39	-22	16.0	no	no	0.090	0.530
Phosphorylation	Tamoxifen citrate	31	-21	16.5	no	no	0.229	0.787
Phosphorylation	TBB	34	-9	19.2	no	no	0.102	0.560
Phosphorylation	Tetramisole hydrochloride	46	9	23.2	no	no	0.816	0.997
Phosphorylation	Tyrphostin 1	41	5	22.3	no	no	0.606	0.997
Phosphorylation	Tyrphostin 23	60	-6	19.9	no	no	0.444	0.985
Phosphorylation	Tyrphostin 25	30	13	24.0	yes	no	0.105	0.563
Phosphorylation	Tyrphostin 47	34	10	23.1	no	no*	0.141	0.661
Phosphorylation	Tyrphostin 51	46	11	23.1	no	no*	0.161	0.697
Phosphorylation	Tyrphostin A9	21	-95	1.1	no	no	0.199	0.755
Phosphorylation	Tyrphostin AG 112	37	3	21.8	no	no	0.426	0.973
Phosphorylation	Tyrphostin AG 126	49	-10	18.7	no	no	0.215	0.776
Phosphorylation	Tyrphostin AG 1478	36	3	21.8	yes	yes	0.064	0.445
Phosphorylation	Tyrphostin AG 34	38	8	22.8	no	no*	0.141	0.661
Phosphorylation	Tyrphostin AG 490	43	6	22.4	no	no	0.262	0.827
Phosphorylation	Tyrphostin AG 494	39	5	22.3	no	no	0.335	0.909
Phosphorylation	Tyrphostin AG 527	36	11	23.5	no	no	0.214	0.776
Phosphorylation	Tyrphostin AG 528	40	5	22.4	yes	no	0.041	0.334
Phosphorylation	Tyrphostin AG 537	21	-8	19.5	no	no	0.149	0.671
Phosphorylation	Tyrphostin AG 538	30	9	22.8	no	no	0.318	0.899
Phosphorylation	Tyrphostin AG 555	50	-7	19.7	no	no	0.470	0.995
Phosphorylation	Tyrphostin AG 698	46	-7	19.7	no	no	0.636	0.997
Phosphorylation	Tyrphostin AG 808	28	-2	20.9	no	no	0.985	0.997
Phosphorylation	Tyrphostin AG 835	46	1	21.5	no	no	0.539	0.997
Phosphorylation	Tyrphostin AG 879	43	-92	1.7	no	no	0.000	0.000
Phosphorylation	U0126	41	-21	16.6	no	no	0.012	0.156
Phosphorylation	Wortmannin	40	17	24.4	yes	no	0.008	0.107
Phosphorylation	Y-27632 dihydrochloride	34	8	23.2	no	no	0.278	0.845
Phosphorylation	ZM 39923 hydrochloride	34	14	24.2	yes	no	0.006	0.090
Prostaglandin	(-)-Naproxen sodium	40	-11	18.4	no	no	0.102	0.560
Prostaglandin	(±)-Ibuprofen	45	1	20.8	no	no	0.924	0.997
Prostaglandin	1-Methylimidazole	46	-2	20.8	no	no	0.903	0.997
Prostaglandin	Acetylsalicylic acid	39	2	23.3	no	no	0.864	0.997

Prostaglandin	Diclofenac sodium	27	2	21.4	no	no	0.484	0.996
Prostaglandin	Etodolac	44	-9	19.1	no	no	0.102	0.560
Prostaglandin	Indomethacin	45	-3	20.0	no	no	0.833	0.997
Prostaglandin	Ketoprofen	61	-1	20.6	no	no	0.774	0.997
Prostaglandin	Ketorolac tris salt	43	0	20.8	no	no	0.783	0.997
Prostaglandin	loxoprofen	47	-5	19.6	no	no	0.833	0.997
Prostaglandin	Meclofenamic acid sodium	48	-10	18.6	no	no	0.055	0.407
Prostaglandin	Meloxicam sodium	43	2	20.9	no	no	0.715	0.997
Prostaglandin	Niflumic acid	47	-13	18.5	no	no	0.003	0.042
Prostaglandin	Nimesulide	27	16	24.5	yes	no	0.018	0.188
Prostaglandin	Oxaprozin	49	-5	20.0	no	no	0.223	0.787
Prostaglandin	Phenylbutazone	40	-11	18.3	no	no	0.063	0.438
Prostaglandin	Piroxicam	26	-2	20.6	no	no	0.898	0.997
Prostaglandin	Resveratrol	42	-18	17.2	no	no	0.090	0.530
Prostaglandin	S(+)-Ibuprofen	42	-1	20.4	no	no	0.777	0.997
Prostaglandin	SC 19220	48	-3	20.2	no	no	0.446	0.985
Prostaglandin	SC-560	33	-14	17.9	no	no	0.001	0.013
Prostaglandin	Sulindac	39	-6	19.7	no	no	0.365	0.937
Prostaglandin	Sulindac sulfone	36	-5	19.9	no	no	0.544	0.997
Serotonin	(R)-(-)-DOI hydrochloride	38	-3	20.4	no	no	0.997	1.000
Serotonin	(±)-8-Hydroxy-DPAT hydrobromide	39	-3	19.4	no	no	0.943	0.997
Serotonin	(±)-DOI hydrochloride	33	5	22.0	no	no	0.587	0.997
Serotonin	1-(1-Naphthyl)piperazine hydrochloride	30	-11	19.0	no	no	0.927	0.997
Serotonin	1-(2-Methoxyphenyl)piperazine hydrochloride	40	8	23.0	no	no	0.200	0.755
Serotonin	1-(3-Chlorophenyl)piperazine dihydrochloride	33	-18	17.8	no	no	0.087	0.530
Serotonin	1-(m-Chlorophenyl)-biguanide hydrochloride	27	10	23.6	no	no	0.727	0.997
Serotonin	1-Phenylbiguanide	34	6	21.9	no	no	0.237	0.787
Serotonin	2-Methyl-5-hydroxytryptamine maleate	50	-7	19.7	no	no	0.895	0.997
Serotonin	3-Tropanyl-3,5-dichlorobenzoate	56	-12	18.3	no	no	0.740	0.997
Serotonin	3-Tropanyl-indole-3-carboxylate hydrochloride	38	0	20.8	no	no	0.785	0.997
Serotonin	3-Tropanylindole-3-carboxylate methiodide	27	1	21.1	no	no	0.539	0.997
Serotonin	5-Carboxamidotryptamine maleate	48	10	23.6	no	no	0.130	0.631
Serotonin	5-Hydroxyindolacetic acid	41	9	21.6	no	no	0.350	0.925
Serotonin	5-Hydroxy-L-tryptophan	31	10	21.9	no	no	0.496	0.996
Serotonin	5-Methoxy DMT oxalate	35	-1	20.3	no	no	0.862	0.997
Serotonin	Alaproclate hydrochloride	40	-3	21.1	no	no	0.785	0.997
Serotonin	alpha-Methyl-5-hydroxytryptamine maleate	41	-1	21.1	no	no	0.990	0.999
Serotonin	Amperozide hydrochloride	38	25	28.5	yes	yes	0.008	0.106
Serotonin	BMY 7378 dihydrochloride	39	2	22.2	no	no	0.423	0.972
Serotonin	BRL 15572	37	16	25.2	yes	yes	0.000	0.010
Serotonin	BRL 54443 maleate	44	6	23.0	no	no	0.387	0.948
Serotonin	Buspirone hydrochloride	44	-2	21.3	no	no	0.689	0.997
Serotonin	BW 723C86	39	-2	21.2	no	no	0.961	0.997
Serotonin	CGS-12066A maleate	39	-13	18.7	no	no	0.093	0.545
Serotonin	Citalopram hydrobromide	41	10	23.9	no	no	0.659	0.997
Serotonin	Clomipramine hydrochloride	37	21	26.4	no	no*	0.152	0.679

Serotonin	Cyclobenzaprine hydrochloride	38	41	30.7	yes	no	0.000	0.000
Serotonin	Cyproheptadine hydrochloride	34	42	30.8	yes	previous	0.000	0.000
Serotonin	Dihydroergotamine methanesulfonate	41	42	30.4	yes	yes	0.000	0.000
Serotonin	Fluoxetine hydrochloride	50	-13	17.5	no	no	0.080	0.502
Serotonin	Fluvoxamine maleate	39	-2	19.9	no	no	0.651	0.997
Serotonin	GR 113808	44	-7	20.1	no	no	0.504	0.996
Serotonin	GR 125487 sulfamate salt	45	3	20.6	no	no	0.746	0.997
Serotonin	GR 127935 hydrochloride	45	-4	19.8	no	no	0.629	0.997
Serotonin	GR 4661	39	-5	21.6	no	no	0.687	0.997
Serotonin	Imipramine hydrochloride	31	-10	18.6	no	no	0.297	0.871
Serotonin	Ketanserin tartrate	39	18	25.0	yes	yes	0.049	0.374
Serotonin	L-Tryptophan	45	-2	20.8	no	no	0.727	0.997
Serotonin	LY-278,584 maleate	43	3	21.2	no	yes	0.653	0.997
Serotonin	LY-310,762 hydrochloride	38	31	27.2	yes	no	0.001	0.011
Serotonin	LY-367,265	25	40	29.1	yes	yes	0.000	0.000
Serotonin	Metergoline	34	23	25.4	yes	yes	0.005	0.068
Serotonin	Methiothepin mesylate	34	33	28.2	yes	previous	0.000	0.000
Serotonin	Methysergide maleate	29	-1	21.0	no	no	0.746	0.997
Serotonin	Mianserin hydrochloride	35	36	27.9	yes	previous	0.000	0.006
Serotonin	ML 10302	42	-5	19.6	no	no	0.827	0.997
Serotonin	N,N-Dipropyl-5-carboxamidotryptamine maleate	40	8	22.7	no	no	0.202	0.758
Serotonin	NAN-190 hydrobromide	41	-6	20.0	no	no	0.412	0.966
Serotonin	N-omega-Methyl-5-hydroxytryptamine oxalate salt	40	6	21.8	no	no	0.926	0.997
Serotonin	O-Methylserotonin hydrochloride	29	8	22.2	no	no	0.585	0.997
Serotonin	PAPP	42	23	26.1	yes	no	0.001	0.024
Serotonin	Paroxetine hydrochloride hemihydrate	51	6	23.1	yes	no	0.099	0.560
Serotonin	Parthenolide	44	-11	18.8	no	no*	0.450	0.988
Serotonin	Pirenperone	40	8	22.2	yes	no	0.019	0.203
Serotonin	p-MPPF dihydrochloride	36	8	22.9	no	no	0.205	0.760
Serotonin	Quipazine dimaleate	39	3	21.1	no	no	0.440	0.985
Serotonin	Quipazine, 6-nitro-, maleate	32	-21	16.2	no	no	0.000	0.001
Serotonin	Quipazine, N-methyl-, dimaleate	45	-18	17.0	no	no	0.001	0.021
Serotonin	R-(-)-Fluoxetine hydrochloride	27	7	21.7	no	no	0.555	0.997
Serotonin	R-(+)-8-Hydroxy-DPAT hydrobromide	43	5	20.9	no	no	0.734	0.997
Serotonin	R(+)-UH-301 hydrochloride	53	7	22.3	no	no	0.245	0.801
Serotonin	Reserpine	26	6	24.2	no	no	0.189	0.743
Serotonin	Ritanserin	45	-16	17.5	no	no	0.000	0.004
Serotonin	Ro 04-6790 dihydrochloride	30	1	21.1	no	no	0.763	0.997
Serotonin	S(-)-UH-301 hydrochloride	30	-1	20.6	no	no	0.652	0.997
Serotonin	S-(+)-Fluoxetine hydrochloride	43	-8	18.6	no	no	0.265	0.831
Serotonin	S15535	36	-4	20.3	no	no	0.256	0.820
Serotonin	SB 200646 hydrochloride	30	-6	20.5	no	no	0.531	0.997
Serotonin	SB 203186	34	6	22.8	no	no	0.551	0.997
Serotonin	SB 204070 hydrochloride	45	7	22.4	yes	no	0.022	0.218
Serotonin	SB 204741	32	-4	20.9	no	no	0.887	0.997

Serotonin	SB 206553 hydrochloride	47	-1	21.1	no	no	0.420	0.968
Serotonin	SB 224289 hydrochloride	49	4	22.1	yes	no	0.101	0.560
Serotonin	SB 228357	25	15	22.9	no	no	0.276	0.843
Serotonin	SB 269970 hydrochloride	38	7	22.3	yes	no	0.127	0.625
Serotonin	SB-215505	50	-6	19.9	no	no	0.913	0.997
Serotonin	SDZ-205,557 hydrochloride	45	-4	20.4	no	no	0.516	0.997
Serotonin	Serotonin hydrochloride	41	16	23.1	yes	no	0.033	0.283
Serotonin	Sertraline hydrochloride	38	-32	14.5	no	no	0.003	0.044
Serotonin	Spiroxatrine	33	14	24.3	yes	no	0.026	0.246
Serotonin	SR 57227A	42	-1	20.6	no	no	0.770	0.997
Serotonin	Trazodone hydrochloride	42	-5	20.1	no	no	0.367	0.937
Serotonin	Trimipramine maleate	31	11	23.5	no	no	0.226	0.787
Serotonin	Tryptamine hydrochloride	46	-5	21.8	no	no	0.267	0.833
Serotonin	VER-3323 hemifumarate salt	37	-24	15.7	no	no	0.035	0.299
Serotonin	WAY-100635 maleate	41	11	23.2	no	no	0.177	0.725
Serotonin	Zimelidine dihydrochloride	46	-1	20.6	no	no	0.945	0.997
Sigma receptor	Opipramol dihydrochloride	36	-2	21.4	no	no	0.771	0.997
Somatostatin	Cysteamine hydrochloride	34	-4	19.7	no	no	0.452	0.989
Sphingolipid	(±)-threo-1-Phenyl-2-decanoylamino-3-morpholino-1-propanol hydrochloride	48	-7	19.0	no	no	0.485	0.996
Sphingolipid	DL-Cycloserine	36	-3	21.2	no	no	0.687	0.997
Sphingolipid	L-Cycloserine	44	-1	21.5	no	no	0.873	0.997
Sphingolipid	N-Oleoylethanolamine	39	1	21.4	no	no	0.790	0.997
Tachykinin	L-703,606 oxalate	31	-46	11.2	no	no	0.000	0.000
Tachykinin	L-732,138	30	5	23.9	no	no	0.706	0.997
Tachykinin	L-733,060 hydrochloride	35	-39	12.6	no	no	0.000	0.007
Tachykinin	SB 222200	41	14	24.8	yes	no	0.119	0.606
Tachykinin	WIN 62,577	39	7	22.3	yes	no	0.101	0.560
Thromboxane	Picotamide	31	6	21.8	no	no	0.677	0.997
Transcription	6(5H)-Phenanthridinone	46	-7	19.1	no	no	0.617	0.997
Transcription	6-Nitroso-1,2-benzopyrone	47	-4	20.3	no	no	0.860	0.997
Transcription	AC-55649	49	-5	19.9	no	no	0.049	0.377
Transcription	Ciprofibrate	40	-12	19.1	no	no	0.121	0.610
Transcription	D-ribofuranosylbenzimidazole	43	-8	19.8	no	no	0.110	0.581
Transcription	Fenofibrate	37	-6	18.9	no	no	0.601	0.997
Transcription	GW1929	33	1	20.5	no	no	0.515	0.997
Transcription	GW7647	33	-13	17.7	no	no	0.145	0.661
Transcription	GW9662	42	-13	17.9	no	no	0.018	0.187
Transcription	TCPOBOP	44	-20	17.0	no	no	0.010	0.128
Transcription	Tetradecylthioacetic acid	48	-10	19.1	no	no	0.001	0.024
Transcription	TTNPB	37	11	23.6	yes	no	0.106	0.570
Vanilloid	Capsazepine	35	0	21.4	no	no	0.989	0.999
Vanilloid	Dihydrocapsaicin	35	-1	20.4	no	no	0.784	0.997
Vanilloid	N-Vanillylnonanamide	29	3	21.5	no	no	0.736	0.997
Vanilloid	SB-366791	49	-12	18.3	no	no	0.067	0.455
Vanilloid	Vanillic acid diethylamide	18	21	25.4	yes	no	0.111	0.581

Supplementary Table 2.
Summary of *C. elegans* lifespan experiments with 57 hit compounds

*mean lifespan in days/ *P* value/ % change in lifespan/ no. of animals
**mean % change in lifespan/ S.D./ no. of experiments/ no. of animals

Class	Compound name	Conc. [uM]	Screen*	Dose-response*	Expt. #1*	Expt. #2*	Expt. #3*	Expt. #4*	Summary**
Antibiotic	Demeclocycline hydrochloride	3		18.0/0.75/ +1%/45					+1%/ --/1/45
		13		19.5/0.21/ +10%/49					+10%/ --/1/49
		33	24.4/0.03/ +16%/43	22.0/0.06/ +14%/61	30.7/2.7E-05/ +19%/59				+16%/3/3/163
		88		25.4/4.0E-05/ +28%/57					+28%/ --/1/57
		176		19.7/0.01/ -6%/65					-6%/ --/1/65
Antibiotic	Doxycycline hydrochloride	0.2		27.0/0.77/ -1%/46					-1%/ --/1/46
		0.6		28.6/0.79/4%/34					+4%/ --/1/34
		3		30.4/0.47/ +11%/32					+11%/ --/1/32
		6		31.2/1.2E-03/ +14%/39					+14%/ --/1/39
		17		32.1/8.0E-03/ +17%/39					+17%/ --/1/39
		33	27.0/2.1E-03/ +29%/38	28.0/0.04/ +2%/38	32.9/5.1E-13/ +32%/43	28.7/2.6E-05/ +11%/52			+18%/14/4/171
Antibiotic	Minocycline hydrochloride	3		17.1/0.26/ -4%/56					-4%/ --/1/56
		13		21.4/4.8E-03/ +21%/54					+21%/ --/1/54
		33	24.4/8.0E-03/ +15%/27	23.7/1.1E-03/ +22%/58					+18%/5/2/85
		88		29.5/7.5E-12/ +49%/63	27.7/0.01/ +14%/71	26.9/3.0E-06/ +24%/70	33.8/1.3E-12/ +31%/61		+29%/15/4/265
		176		26.0/0.01/ +24%/55					+24%/ --/1/55
Biochemistry	3,4-Dichloroisocoumarin	3		17.9/0.95/ +0%/61					0%/ --/1/61
		13		18.1/0.95/ +3%/60					+3%/ --/1/60
		33	26.9/0.012/ +28%/29	24.5/1.4E-03/ +26%/39					+27%/10/1.4/68
		56			24.7/7.1E-04/ +14%/74				+14%/ --/1/74
		88		28.1/2.1E-07/ +41%/48	20.3/0.49/ -17%/85	29.8/4.3E-03/ +16%/33			+13%/29/3/166
		176		10.8/3.9E-08/ -48%/36					-48%/ --/1/36
Biogenic Amine/ Adrenoceptor	Amoxapine	3		21.8/0.2/ +21%/41					+21%/ --/1/41
		13		26.5/4.7E-08/ +50%/41					+50%/ --/1/41
		33	30.4/3.4E-08/ +39%/40	27.3/1.4E-06/ +41%/44					+40%/1/2/84
		88		32.8/1.5E-15/ +65%/43	28.5/4.4E-05/ +17%/83	28.4/1.9E-07/ +31%/65	30.6/4.4E-05/ +19%/68		+33%/22/4/259
		176		25.9/0.025/ +24%/47					+24%/ --/1/47
Biogenic Amine/ Adrenoceptor	Doxazosin mesylate	3		15.9/0.3/ -11%/42					-11%/ --/1/42
		13		20.1/2.6E-03/ +14%/57					+14%/ --/1/57

		33	23.2/1.8E-03/ +11%/38	22.1/3.4E-04/ +14%/46	30.9/3.9E-08/ +20%/52				+15%/5/3/136
		88		19.1/0.09/ -4%/54					-4%/ --/1/54
		176		13.2/2.2E-11/ -37%/59					-37%/ --/1/59
Biogenic Amine/ Adrenoceptor	Guanabenz acetate	3		19.9/0.04/ +11%/48					+11%/ --/1/48
		13		19.5/0.027/ +10%/76					+10%/ --/1/76
		33	23.6/5.7E-03/ +19%/43	18.1/0.5/ -7%/50					+6%/18/2/93
		88		23.3/1.9E-04/ +17%/61	29.3/5.5E-12/ +21%/90	23.7/0.011/ +10%/69	26.1/0.8/ +1%/53		+12%/9/4/273
		176		23.5/8.5E-04/ +12%/60					+12%/ --/1/60
Biogenic Amine/ Adrenoceptor	Guanfacine hydrochloride	3		18.4/0.47/ +2%/67					+2%/ --/1/67
		13		21.8/2.1E-04/ +23%/55	25.1/0.39/ +3%/66	26.7/1.8E-04/ +23%/55	28.2/0.03/ +10%/51		+15%/10/4/227
		33	25.7/1.4E-03/ +27%/43	23.3/4.0E-03/ +20%/47					+24%/ --/5/2/90
		88		24.3/9.9E-05/ +22%/61					+22%/ --/1/61
		176		23.8/1.8E-03/ +13%/44					+13%/ --/1/44
Biogenic Amine/ Adrenoceptor	Naftopidil dihydrochloride	3		20.0/0.06/ +12%/63					+12%/ --/1/63
		13		19.4/0.02/ +10%/68					+10%/ --/1/68
		33	24/1.0E-03/ +14%/30	23.7/1.1E-04/ +22%/43	27.6/0.11/ +7%/62				+14%/8/3/135
		88		26.1/2.8E-07/ +31%/57					+31%/ --/1/57
		176		28.8/1.1E-09/ +37%/57					+37%/ --/1/57
Biogenic Amine/ Adrenoceptor	Nortriptyline hydrochloride	3		18.8/0.55/ +5%/60					+5%/ --/1/60
		13		17.9/0.66/ +1%/62					+1%/ --/1/62
		33	26.7/5.1E-09/ +26%/41	22.4/0.026/ +15%/56					+21%/8/2/97
		88		26.9/3.9E-08/ +35%/71	31.1/1.7E-07/ +28%/73	25.5/0.014/ +18%/75	26.1/0.98/ +1%/49		+21%/15/4/268
		176		26.6/0.048/ +26%/51					+26%/ --/1/51
Biogenic Amine /Histamine	Loratadine	3		+17.7/0.7/ -1%/54					-1%/ --/1/54
		13		19.7/0.07/ +11%/63					+11%/ --/1/63
		33	25.4/7.2E-04/ +23%/29	23.0/3.8E-05/ +18%/57	28.8/0.47/ +12%/42				+18%/6/3/128
		88		26.9/5.9E-09/ +35%/58					+35%/ --/1/58
		176		21.3/0.95/ +2%/46					+2%/ --/1/46
Biogenic Amine /Histamine	Oxatomide	3		21.4/0.04/ +19%/48	25.7/0.07/ -6%/38				+7%/18/2/86
		13		25.1/1.1E-07/ +42%/55	29.8/0.03/ +9%/44				+26%/23/2/99
		33	27.6/6.9E-09/ +31%/35	25.1/1.6E-07/ +29%/52	30.3/1.4E-03/ +11%/44				+24%/11/3/131
		88		28.9/3.9E-12/ +46%/69	31.7/1.6E-05/ +16%/49	31.7/3.9E-15/ +30%/94	27.4/8.1E-09/ +27%/96	27.6/0.25/ +7%/58	+25%/15/5/366
		176		32.0/4.8E-12/ +53%/59	29.7/0.17/ +8%/50				+31%/32/2/109
Biogenic Amine /Histamine	Promethazine hydrochloride	3		21.2/0.1/ +18%/48					+18%/ --/1/48

		13		17.1/0.6/ -3%/65					-3%/ --/1/65
		33	25.7/2.7E-03/ +22%/34	24.3/1.7E-04/ +25%/60					+24%/2/2/94
		88		27.5/3.4E-06/ +38%/44					+38%/ --/1/44
		176		32.0/2.0E-13/ +52%/68	31.8/6.9E-10/ +31%/79	29.4/1.3E-08/ +36%/67	28.2/0.9/ +10%/51		+32%/17/4/265
Biogenic Amine /Dopamine	(±)-Octoclothepin maleate	3		27.6/ 4.3E-13/ +54%/62					+54%/ --/1/62
		13		29.9/2.2E-23/ +69%/73	32.4/2.1E-03/ +31%/52	28.8/2.6E-11/ +33%/76	30.3/3.7E-04/ +18%/57		+38%/22/4/258
		33	28.1/1.1E-12/ +33%/39	31.1/6.5E-19/ +60%/60					+47%/19/2/99
		88		23.9/6.1E-03/ +20%/61					+20%/ --/1/61
		176		11.5/1.9E-15/ -45%/53					-45%/ --/1/53
Biogenic Amine /Dopamine	BTCP hydrochloride	3		17.9/0.83/0%/47					0%/ --/1/47
		13		18.0/0.78/ +2%/53					+2%/ --/1/53
		33	26.4/1.4E-07/ +22%/51	22.8/1.2E-03/ +18%/58					+20%/3/2/109
		88		22.1/0.05/ +11%/35	29.2/0.06/ +18%/47	26.7/9.1E-08/ +23%/63	27.1/0.07/ +5%/63		+14%/8/4/208
		176		18.3/0.42/ -13%/50					-13%/ --/1/50
Biogenic Amine /Dopamine	Chlorprothixene hydrochloride	3		26.2/7.1E-06/ +46%/44					+46%/ --/1/44
		13		28.2/1.7E-12/ +59%/45	31.4/8.7E-04/ +27%/67	30.6/4.3E-13/ +42%/66	26.5/0.76/ +3%/59		+33%/24/4/237
		33	32.5/3.9E-18/ +50%/43	28.9/3.9E-11/ +49%/44					+50%/1/2/87
		88		26.6/9.3E-06/ +34%/52					+34%/ --/1/52
		176		26.5/1.8E-03/ +26%/57					+26%/ --/1/57
Biogenic Amine /Dopamine	cis-(Z)-Flupenthixol dihydrochloride	3		21.7/0.46/ +21%/59					+21%/ --/1/59
		13		26.7/3.3E-11/ +51%/59					+51%/ --/1/59
		33	30.0/1.7E-08/ +48%/38	30.6/3.4E-17/ +57%/53	28.6/0.09/ +16%/57	28.0/5.9E-09/ +30%/67	25.3/0.35/ -2%/53		+30%/24/5/268
		88		31.2/6.4E-15/ +57%/57					+57%/ --/1/57
		176		19.9/0.14/ -5%/41					-5%/ --/1/41
Biogenic Amine /Dopamine	Cortexolone maleate	3		19.1/0.14/ +6%/56					+6%/ --/1/56
		13		18.9/0.15/ +7%/46					+7%/ --/1/46
		33	24.3/3.9E-10/ +16%/47	22.0/8.8E-04/ +13%/53	25.0/0.55/ +5%/74				+11%/6/3/174
		88		24.2/2.6E-05/ +22%/50					+22%/ --/1/50
		176		24.0/6.2E-04/ +14%/65					+14%/ --/1/65
Biogenic Amine /Dopamine	Dihydroergocristine methanesulfonate	3		20.4/0.08/ +14%/53					+14%/ --/1/53
		13		22.9/1.3E-03/ +30%/40					+30%/ --/1/40
		33	29.4/8.3E-08/ +40%/32	24.5/1.1E-04/ +26%/46					+33%/10/2/78
		88		27.7/3.2E-08/ +39%/53					+39%/ --/1/53

		176		31.0/1.5E-11/ +48%/67	33.1/5.5E-05/ +34%/71	30.3/2.0E-12/ +40%/57	29.6/3.7E-03/ +15%/53		+34%/14/4/248
Biogenic Amine /Dopamine	Loxapine succinate	3		20.2/0.46/ +13%/59					+13%/ --/1/59
		13		23.7/1.4E-04/ +34%/50					+34%/ --/1/50
		33	25.4/8.0E-06/ +23%/41	25.5/8.9E-06/ +31%/58					+27%/6/2/99
		88		30.3/5.3E-12/ +53%/47	36.1/1.9E-08/ +46%/59	33.5/6.1E-21/ +55%/65	30.2/6.9E-06/ +17%/80		+43%/18/4/251
		176		31.7/1.3E-10/ +51%/39					+51%/ --/1/39
Biogenic Amine /Dopamine	Methylergonovine maleate	3		20.7/0.06/ +15%/63					+15%/ --/1/63
		13		22.6/1.2E-04/ +28%/59					+28%/ --/1/59
		33	26.0/5.9E-04/ +26%/40	28.5/1.0E-09/ +47%/48					+37%/15/2/88
		88		29.9/1.1E-13/ +51%/70	29.8/0.11/ +21%/71	27.4/5.2E-06/ +27%/60	29.3/0.02/ +14%/61		+28/%/16/4/262
		176		30.1/1.0E-06/ +43%/51					+43%/ --/1/51
Biogenic Amine /Dopamine	N-(2-[4-(4-Chlorophenyl)piperazin-1-yl]ethyl)-3-methoxybenzamide	3		21.1/0.09/ +18%/42					+18%/ --/1/42
		13		23.7/8.3E-08/ +34%/57					+34%/ --/1/57
		33	28.1/1.7E-06/ +29%/41	28.3/3.2E-12/ +46%/44	32.7/3.5E-05/ +32%/54	32.2/3.2E-18/ +49%/57	30.8/2.8E-07/ +20%/61		+35%/12/5/257
		88		27.0/1.3E-09/ +36%/47					+36%/ --/1/47
		176		29.6/5.8E-10/ +41%/50					+41%/ --/1/50
Biogenic Amine /Dopamine	Pergolide methanesulfonate	3		24.9/4.5E-07/ +39%/54					+39%/ --/1/54
		13		30.5/1.5E-19/ +72%/50	29.8/0.04/ +21%/63	30.5/1.8E-11/ +41%/56	29.3/1.0E-04/ +14%/83		+37%/26/4/252
		33	27.2/4.4E-10/ +32%/37	31.6/1.1E-19/ +63%/54					+48%/22/2/91
		88		30.6/9.9E-14/ +54%/54					+54%/ --/1/54
		176		30.2/7.4E-11/ +44%/67					+44%/ --/1/67
Biogenic Amine /Dopamine	Propionylpromazine hydrochloride	3		22.3/0.01/ +24%/44					+24%/ --/1/44
		13		23.8/4.5E-06/ +35%/48					+35%/ --/1/48
		33	25.6/1.4E-07/ +24%/46	26.4/5.9E-08/ +36%/55	25.6/0.06/ -1%/64				+20%/19/3/165
		88		30.4/4.9E-15/ +53%/57					+53%/ --/1/57
		176		24.1/0.01/ +15%/44					+15%/ --/1/44
Biogenic Amine /Dopamine	Thioridazine hydrochloride	3		18.8/0.74/ +5%/46					+5%/ --/1/46
		13		23.9/8.3E-06/ +35%/51					+35%/ --/1/51
		33	28.1/5.1E-15/ +34%/42	29/2.5E-13/ +49%/57	25.7/0.09/ +9%/92				+31%/20/3/191
		88		31.2/8.9E-16/ +57%/55					+57%/ --/1/55
		176		19.3/0.67/ -8%/59					-8%/ --/1/59

Biogenic Amines /Serotonin	Amperozide hydrochloride	3		25.3/5.7E-08/ +41%/53					+41%/ --/1/53
		13		30.1/1.3E-16/ +70%/39	28.9/3.0E-04/ +19%/73	30.8/5.7E-19/ +42%/85	30.8/5.6E-09/ +20%/61		+38%/24/4/258
		33	28.5/5.2E-04/ +25%/38	32.6/4.1E-24/ +68%/63					+47%/30/2/101
		88		32.5/1.6E-22/ +64%/64					+64%/ --/1/64
		176		32.3/3.3E-17/ +54%/59					+54%/ --/1/59
Biogenic Amines /Serotonin	BRL 15572	3		29.6/0.3/ +6%/74					+6%/ --/1/74
		13		32.8/9.9E-06/ +17%/58					+17%/ --/1/58
		33	25.2/1.9E-06/ +16%/37	29.2/0.12/ +5%/75	28.5/1.5E-03/ +11%/59	26.3/5E-05/ +6%/50			+10%/5/4/221
		88		21.5/3.8E-10/ -23%/80					-23%/ --/1/80
		176		18.3/5.5E-30/ -34%/84					-34%/ --/1/84
Biogenic Amines /Serotonin	Dihydroergotamine methanesulfonate	3		22.4/6.5E-03/ +25%/35					+25%/ --/1/35
		13		23.0/7.1E-05/ +30%/61					+30%/ --/1/61
		33	30.4/1.7E-10/ +42%/41	22.1/0.08/ +14%/48					+28%/2/2/89
		88		28.7/9.9E-07/ +44%/38	31.0/4.8E-08/ +27%/85	23.2/0.35/ +7%/77	30.4/5.0E-03/ +18%/61		+24%/20/4/261
		176		27.4/7.7E-04/ +30%/54					+30%/ --/1/54
Biogenic Amines /Serotonin	Ketanserin tartrate	3		20.1/0.36/ +12%/49					+12%/ --/1/49
		13		19.5/0.49/ +10%/49					+10%/ --/1/49
		33	25/6.3E-03/ +18%/39	21.0/0.19/ +8%/55					+13%/7/2/94
		88		24.2/3.4E-03/ +22%/55					+22%/ --/1/55
		176		25.9/1.3E-03/ +23%/59	27.9/0.02/ +15%/66	25.3/0.04/ +17%/69	25.2/0.47/ -2%/61		+13%/11/4/255
Biogenic Amines /Serotonin	LY-165,163	3		19.8/0.5/ +10%/48					+10%/1/48
		13		25.2/1.1E-08/ +42%/59					+42%/ --/1/59
		33	26.1/8.8E-06/ +23%/42	31.3/2.1E-18/ +61%/55	31.9/3.4E-13/ +31%/77	29.8/2.2E-10/ +38%/70	29.1/0.03/ +13%/65		+33%/18/5/309
		88		+30.1/2.6E-11/ +52%/49					+52%/ --/1/49
		176		32.3/6.9E-14/ +54%/63					+54%/ --/1/63
Biogenic Amines /Serotonin	LY-367,265	3		23.4/1.3E-06/ +34%/106					+34%/ --/1/106
		13		28.8/1.5E-20/ +64%/103					+64%/ --/1/103
		33	29.1/5.3E-11/ +40%/25	28.2/5.7E-1/ +61/%89	31.7/1.6E-09/ +28%/44	27.4/0.05/ +7%/51			+34%/23/4/209
		88		29.1/2.0E-18/ +66%/88					+66%/ --/1/88
		176		28/1.9E-13/ +60%/67					+60%/ --/1/67
Biogenic Amines /Serotonin	Metergoline	3		22.6/0.017/ +26%/37					+26%/ --/1/37
		13		22.8/2.2/ +29%/51					+29%/ --/1/51
		33	25.4/1.2E-04/ +23%/34	25.6/5.2E-05/ +32%/43	28.9/1.4E-03/ +13%/65				+23%/10/3/142

		88		23.8/1.1E-05/ +20%/63					+20%/ --/1/63
		176		13.1/1.9E-05/ -38%/49					-38%/ --/1/49
Biogenic Amines /Serotonin	Mianserin hydrochloride	3		23.8/3.0E-03/ +31%/66					+31%/ --/1/66
		13		29.0/1.1E-15/ +59%/89					+59%/ --/1/89
		33	27.9/3.7E-07/ +36%/35	32.1/7.3E-22/ +76%/75	29.7/0.037/ +15%/57				+42%/31/3/167
		50		31.5/1.7E-16/ +30%/138	30.7/6.4E-35/ +42%/245	31.0/6.2E-03/ +25%/68			+32%/9/3/451
		88		30.7/4.1E-21/ +68%/99					+68%/ --/1/99
		176		27.7/6.5E-13/ +52%/84					+52%/ --/1/84
Ca2+ Channel	Cinnarizine	3		19.9/0.06/ +10%/43					+10%/ --/1/43
		13		19.9/0.02/ +11%/53					+11%/ --/1/53
		33	24.1/1.2E-03/ +11%/33	20.6/6.6E-05/ +14%/51	28.9/8.5E-05/ +16%/57	28.7/6.51E-08/ +18%/79	26.7/1.8E-04/ +24%/63	27.8/0.15/ +8%/62	+15%/6/6/345
		88		14.2/0.011/ -21%/49					-21%/ --/1/49
		176		9.83/6.8E-12/ -45%/46					-45%/ --/1/46
Ca2+ Channel	Nicardipine hydrochloride	3		17.9/0.69/0%/36					+0%/ --/1/36
		13		19.2/0.23/ +9%/41					+9%/ --/1/41
		33	25.4/1.3E-05/ +20%/31	20.1/0.47/ +4%/55					+12%/11/2/86
		88		20.8/0.12/ +5%/58					+5%/ --/1/58
		176		27.2/6.9E-06/ +30%/44	27.7/1.2E-08/ +14%/74	26.7/6.9E-05/ +24%/67			+23%/8/3/185
Ca2+ Channel	Nitrendipine	3		17.1/0.75/ -4%/58					-4%/ --/1/58
		13		20.1/0.03/ +14%/57					+14%/ --/1/57
		33	23.6/5.1E-04/ +12%/42	27.2/1.3E-10/ +40%/52					+26%/20/2/94
		88		33.3/5.5E-18/ +68%/45	27.1/2.6E-04/ +11%/79	25.6/5.51E-06/ +18%/91	26.8/0.18/ +4%/56		+25%/29/4/271
		176		25.5/4.2E-04/ +21%/66					+21%/ --/1/66
Cholinergic	Hexahydro-sila-difenidol hydrochloride	3		19.8/0.09/ +11%/63					+11%/ --/1/63
		13		20.7/0.02/ +17%/58					+17%/ --/1/58
		33	24.9/5.4E-04/ +25%/43	20.5/0.23/ +6%/55					+16%/13/2/98
		88		24.2/8.1E-03/ +22%/45	28.8/1.6E-07/ +18%/87	24.0/0.04/ +11%/74	27.4/0.47/ +7%/70		+15%/7/4/276
		176		24.1/0.07/ +15%/64					+15%/ --/1/64
Cyclic Nucleotides	BRL 50481	3		18.4/0.87/ +2%/52	26.3/0.63/ -4%/54				-1%/4/2/106
		13		17.7/0.46/0%/51	28/0.76/ +2%/48				+1%/1/2/99
		33	25.1/0.025/ +26%/27	18.4/0.61/ -5%/60	26.4/0.94/ -4%/46				+6%/18/3/133
		88		22.6/5.31E-04/ +14%/52	29.6/0.082/ +8%/49				+11%/4/2/101
		176		27.4/1.1E-08/ +31%/46	31.0/3.2E-03/ +13%/45	28.8/9.3E-10/ +19%/76	23.6/1.0E-03/ +9%/67	30.9/7.9E-11/ +20%/78	+18%/8/5/312
		331			31.0/0.03/ +13%/40				+13%/ --/1/40
		533			28.9/0.06/ +6%/50				+6%/ --/1/50

Cyclic Nucleotides	Trequinsin hydrochloride	3		20.2/0.11/ +13%/58					+13%/ --/1/58
		13		22.1/3.8E-04/ +25%/43					+25%/ --/1/43
		33	24.3/2.9E-04/ +14%/52	23.6/2.9E-05/ +22%/64					+18%/6/2/116
		88		27.3/3.5E-09/ +37%/56	31.9/6.9E-15/ +31%/76	26.0/2.3E-06/ +20%/77	31.1/2.9E-09/ +21%/63		+27%/8/4/272
		176		26.6/1.3E-05/ +27%/56					+27%/ --/1/56
Cyclic Nucleotides	Vinpocetine	3		23.9/0.38/ +15%/28					+15%/ --/1/28
		13		24.8/0.034/ +19%/30					+19%/ --/1/30
		33	24.4/1.3E-03/ +17%/25	25.2/0.03/ +21%/41	29.5/1.2E-04/ +19%/48	28.0/1.5E-05/ +15%/82	22.8/0.5/ +5%/71	29.6/5.9E-05/ +15%/71	+15%/6/6/338
		88		23.5/0.011/ +13%/43					+13%/ --/1/43
		176		23.5/0.06/ +13%/37					+13%/ --/1/37
Cytoskeleton and ECM	Vincristine sulfate	3		19.3/0.19/ -7%/46					-7%/ --/1/46
		13		23.5/0.83/ +13%/51					+13%/ --/1/51
		33	24.8/4.6E-03/ +19%/33	22.6/0.8/ +8%/50	26.8/0.08/ +8%/64				+12%/6/3/147
		88		24.7/0.025/ +19%/46	29.1/3.6E-04/ +20%/78	24.3/0.04/ +13%/63	25.1/0.26/ -3%/60		+12%/11/4/247
		176		23.6/0.2/ +13%/41					+13%/ --/1/41
Glutamate	AMN082	3		18.1/0.7/ +1%/42	30.9/0.04/ +7%/61				+4%/4/2/103
		13		17.2/0.7/ -3%/64	32.6/8.9E-04/ +13%/61				+5%/11/2/125
		33	24.7/5.0E-07/ +16%/43	19.0/0.15/ -2%/55	29.8/6.7E-05/ +16%/73	29.2/0.8/ +1%/73			+8%/10/4/244
		88		20.1/0.02/ +1%/62	25.4/6.6E-03/ -12%/94				-6%/9/2/156
		176		16.6/0.04/ -21%/55	19.1/2.0E-10/ -34%/80				-27%/9/2/135
Glutamate	Eliprodil	3		15.8/6.9E-03/ -11%/71					-11%/ --/1/71
		13		16.7/0.07/ -6%/59					-6%/ --/1/59
		33	24.5/0.019/ +17%/43	22.6/0.04/ +16%/48					+16%/1/2/91
		88		25.0/3.7E-04/ +26%/50	27.8/0.01/ +14%/66	25.7/9.0E-04/ +19%/79	27.1/0.29/ +5%/51		+16%/9/4/246
		176		23.1/0.13/ +10%/48					+10%/ --/1/48
Hormone	(R,R)-cis-Diethyl tetrahydro-2,8-chrysenediol	3		20.6/0.09/ +8%/82					+8%/ --/1/82
		13		19.7/0.49/ +3%/70					+3%/ --/1/70
		33	23.6/ 0.022/ +13%/26	19.1/0.86/ -1%/76	27.3/0.26/ +6%/67	26.9/0.02/ +8%/46			+7%/6/4/215
		88		21.5/9.0E-03/ +12%/90					+12%/ --/1/90
		176		21.5/0.013/ +12%/88					+12%/ --/1/88
Hormone	beta-Estradiol	3		18.7/0.97/ +4%/45	29.0/0.72/ +6%/30				+5%/1/2/75
		13		20.5/0.03/ +16%/62	28.9/0.8/ +6%/36				+11%/7/2/98
		33	24/0.02/ +19%/39	20.8/0.17/ +7%/54	31.3/0.029/ +14%/28	24.8/0.022/ +2%/96	20.9/0.77/ -3%/81	26.7/0.07/ +4%/58	+7%/8/6/356
		88		20.6/0.22/ +4%/62	29.5/0.019/ +8%/42				+6%/3/2/104
		176		25.0/3.9E-03/ +19%/32	27.4/0.76/0%/48				+10%/13/2/80

Hormone	Cyproterone acetate	3		19.2/0.41/ +7%/54	27.4/0.91/ +0%/44				+4%/5/2/98
		13		19.6/4.8E-03/ +11%/71	28.8/0.019/ +5%/51				+8%/4/2/122
		33	27.4/1.4E-05/ +26%/37	26.4/2.3E-07/ +36%/58	29.4/0.89/ +7%/42				+23%/15/3/137
		88		27.9/1.2E-07/ +41%/50	28.8/0.66/ +5%/37				+23%/25/2/87
		176		30.8/6.7E-10/ +46%/67	28.0/0.95/ +2%/45	29.5/6.6E-07/ +21%/79	30.8/7.2E-11/ +42%/59	28.5/2.9E-03/ +11%/65	+24%/19/5/315
Hormone	Danazol	3		17.1/0.36/ -5%/45					-5%/ --/1/45
		13		18.2/0.34/ +3%/34					+3%/ --/1/34
		33	22.12/0.02/ +6%/49	24.74/4.3E-05/ +28%/38	27.6/3.1E-06/ +13%/65	22.5/0.12/ +4%/65	29.7/9.9E-04/ +16%/47		+13%/10/5/264
		88		22.8/1.4E-04/ +15%/67					+15%/ --/1/67
		176		20.6/0.39/ -2%/51					-2%/ --/1/51
K+ Channel	Psora-4	3		20.8/1.7E-03/ +16%/48					+16%/ --/1/48
		13		23.8/1.9E-09/ +35%/71					+35%/ --/1/71
		33	27.5/3.6E-07/ +26%/46	30.0/7.5E-18/ +55%/62					+40%/21/2/108
		88		32.5/6.6E-17/ +64%/49	31.5/1.2E-13/ +30%/74	30.2/2.9E-18/ +40%/74	34.0/1.4E-18/ +32%/68		+42%/16/4/265
		176		33.1/3.1E-17/ +58%/52					+58%/ --/1/52
K+ Channel	Quinidine sulfate	3		20.5/0.03/ +14%/63					+14%/ --/1/63
		13		20.4/0.02/ +16%/51					+16%/ --/1/51
		33	24.2/8.0E-03/ +18%/35	22.0/4.4E-03/ +14%/50	26.7/5.4E-03/ +10%/80	24.6/0.04/ +14%/64	26.7/0.4/ +4%/65		+12%/5/5/294
		88		21.3/0.21/ +7%/50					+7%/ --/1/50
		176		22.3/0.19/6%/72					+6%/1/72
Nitric Oxide	4-Phenyl-3-furoxan-carbonitrile	3		21.5/3.4E-03/ +20%/47					+20%/ --/1/47
		13		21.4/2.0E-04/ +21%/59					+21%/ --/1/59
		33	26.3/1.1E-04/ +24%/34	21.9/6.4E-03/ +13%/51					+19%/8/2/85
		88		28.1/1.2E-10/ +42%/58	30.4/6.7E-08/ +25%/79	28.7/1.7E-09/ +33%/74	30.9/5.6E-05/ +20%/62		+30%/10/4/273
		176		14.0/3.5E-04/ -34%/57					-34%/ --/1/57
Phosphory-lation	7-Cyclopentyl-5-(4-phenoxy) phenyl-7H-pyrrolo[2,3-d]pyrimidin-4-ylamine	3		18.3/0.8/ +2%/46					+2%/ --/1/46
		13		17.1/0.8/ -3%/52					-3%/ --/1/52
		33	23.8/8.3E-03/ +14%/25	22.0/1.3E-03/ +14%/51	27.3/5.6E-04/ +6%/64				+11%/5/3/140
		88		21.8/6.7E-04/ +10%/58					+10%/ --/1/58
		176		21.8/0.38/ +4%/48					+4%/ --/1/48
Phosphory-lation	Cyclosporin A	3		21.2/0.03/ +18%/38					+18%/ --/1/38
		13		20.4/0.01/ +15%/53					+15%/ --/1/53
		33	24.9/9.98E-04/ +14%/41	27.0/4.1E-08/ +39%/37					+27%/18/2/78

		48		26.9/4.5E-07/ +24%/72					+24%/ --/1/72
		88		27.7/3.7E-10/ +39%/55	26.8/0.048/ +10%/64	27.3/0.22/ +6%/53			+18%/18/3/172
		176		21.8/0.84/ +4%/51					+4%/ --/1/51
Phosphory-lation	DAPH	3		22.8/4.2E-03/ +27%/38					+27%/ --/1/38
		13		22.6/6.4E-05/ +28%/48	27.6/2.9E-04/ +14%/85	25.7/4.3E-03/ +19%/72	25.4/0.7/ -1%/63		+15%/12/4/268
		33	24.8/2.36E-03/ +14%/47	21.4/0.06/ +10%/49					+12%/3/2/96
		88		21.1/0.6/ +6%/49					+6%/ --/1/49
		176		17.4/1.4E-04/ -17%/55					-17/%--/1/55
Phosphory-lation	Kenpaullone	3		21.9/8.3E-04/ +22%/46					+22%/ --/1/46
		13		20.3/5.7E-03/ +15%/56					+15%/ --/1/56
		33	25.0/8.76E-06/ +21%/45	24.0/1.1E-04/ +24%/46					+23%/2/2/91
		88		27.7/2.5E-09/ +40%/49	30.3/5.3E-08/ +25%/57	29.6/3.0E-11/ +37%/58	27.6/4.7E-03/ +7%/54		+27%/15/4/218
		176		27.6/1.3E-07/ +32%/55					+32%/ --/1/55
Phosphory-lation	LFM-A13	3		19.4/0.21/ +8%/51					+8%/ --/1/51
		13		16.3/0.9/ -8%/62					-8%/ --/1/62
		33	25.4/2.5E-05/ +23%/35	23.2/1.1E-04/ +19%/45					+21%/3/2/80
		88		27.1/1.20E-09/ +37%/55	30.9/3.2E-13/ +27%/81	28.6/7.8E-12/ +32%/76	28.3/0.35/ +10%/54		+27%/12/4/266
		176		28/9.4E-06/ +33%/43					+33%/ --/1/43
Phosphory-lation	SU 4312	3		17/0.9/ -5%/47					-5%/ --/1/47
		13		21.3/3.1E-04/ +20%/44					+20%/ --/1/44
		33	24.6/1.6E-07/ +16%/29	20.6/8.4E-03/ +6%/55					+11%/7/2/84
		88		25.7/2.7E-06/ +29%/45	26.9/1.2E-06/ +11%/74	15.3/1.0E-03/ -29%/73	28.2/0.01/ +10%/61		+5%/24/4/253
		176		23.42/2.9E-03/ +12%/50					+12%/ --/1/50
Phosphory-lation	Tyrphostin AG 1478	3		23.0/2.6E-03/ +28%/43					+28%/ --/1/43
		13		20.5/0.09/ +16%/55					+16%/ --/1/55
		33	21.8/0.01/ +3%/36	23.2/5.9E-04/ +19%/54	30.0/1.3E-08/ +23%/70	20.1/0.7/ -7%/87	29.8/1.3E-03/ +16%/59		+11%/12/5/306
		88		22.7/1.8E-03/ +14%/50					+14%/ --/1/50
		176		20.2/0.96/ -4%/57					-4%/ --/1/57

Supplementary Table 3.
Summary of stress resistance experiments in *C. elegans*

Class	Compund name	Conc. [uM]	Live animals (%) (expt. 1)	Live animals (%) (expt. 2)	Live animals (%) (expt. 3)	Live animals (%) (expt. 4)	Mean % of animals alive (4 expts)*	S.D., % animals alive (4 expts)	No. wells	No. animals	Mean % of animals alive (all)**
Antibiotic	Demeclocycline	33	62.7	52.5	45.9	63.4	56.9	19.7	26	236	54.7
	Doxycycline	33	49.7	61.2	54.9	66.3	57.6	21.6	26	231	60.2
	Minocycline	88	15.8	45.4	35.0	11.4	26.4	22.8	26	252	24.2
Biochemistry	3,4-Dichloro-isocoumarin	88	53.9	51.4	43.9	62.1	53.2	14.9	26	251	51.8
Biogenic Amine /Adrenoceptor	Amoxapine	88	74.7	87.1	75.5	69.9	77.1	17.7	26	249	78.3
	Doxazosin	33	44.6	45.8	49.6	29.6	42.4	24.6	26	236	41.5
	Guanabenz	88	52.3	54.4	42.7	61.2	53.1	20.7	26	235	51.5
	Guanfacine	13	58.0	48.1	50.4	43.7	50.6	21.3	26	242	50.4
	Naftopidil	33	55.5	45.9	45.1	64.7	53.1	20.1	26	224	52.2
	Nortriptyline	88	58.2	72.9	51.7	77.8	65.4	16.7	26	230	64.8
Biogenic Amine /Dopamine	(±)-Octoclothepin	13	80.6	84.7	74.7	89.0	82.5	14.2	26	284	83.1
	BTCP	88	37.5	57.5	50.5	63.6	51.4	20.1	26	255	49.0
	Chlorprothixene	13	74.7	90.7	58.9	69.0	74.7	20.2	26	269	76.2
	cis-(Z)-Flupenthixol	33	64.4	80.1	71.8	84.7	74.7	17.8	26	249	76.3
	Cortexolone	33	59.4	18.3	22.7	34.5	34.2	21.6	31	301	32.9
	Dihydroergocristine	176	59.1	83.5	57.9	68.0	67.5	21.6	26	270	69.3
	Loxapine	88	71.5	91.7	70.6	85.6	80	16.8	26	255	79.2
	Methylergonovine maleate	88	79.2	91.1	78.6	69.5	80.1	11.1	26	240	82.1
	N-(2-[4-(4-Chlorophenyl)piperazin- 1-yl]ethyl)-3-methoxybenzamide	33	87.5	82.4	80.0	71.2	80.9	13.4	26	240	80.0
	Pergolide	13	74.1	82.7	82.2	73.2	77.8	12.6	26	281	78.6
	Propionylpromazine	33	61.3	61.6	91.7	73.8	70.1	18.8	26	297	70.7
	Thioridazine	33	54.8	56.6	26.2	69.0	51.4	23.9	30	258	50.8
Biogenic Amine /Histamine	Loratadine	33	59.5	40.1	55.1	64.4	54.6	18.1	26	257	56.0
	Oxatomide	88	56.5	61.5	72.5	79.0	66.1	19.9	26	252	67.9
	Promethazine	176	65.7	78.9	67.1	79.8	72.8	16	26	250	72.1
Biogenic Amine /Serotonin	Amperozide	13	62.7	63.4	66.4	55.3	61.9	14.2	26	260	63.8
	BRL 15572	33	50.7	41.1	42.3	45.4	45.3	20.1	26	232	45.7
	Dihydroergotamine	88	61.8	70.4	68.3	75.2	68.5	18.4	26	245	67.3
	Ketanserin	176	51.0	68.2	42.8	64.6	57.2	17.9	26	254	55.5
	LY-165,163	33	63.1	80.6	86.3	40.8	67.1	23.9	26	244	73.0
	LY-367,265	33	70.9	77.5	73.3	69.6	72.8	15.7	26	284	72.9
	Metergoline	33	58.3	61.5	75.7	61.2	63.2	18.3	26	250	65.6
	Mianserin	33	48.5	85.7	63.5	74.2	67.3	21.8	26	239	70.3
Ca2+ Channel	Cinnarizine	33	51.5	32.3	39.6	51.3	44	20.9	26	290	43.1
	Nicardipine	33	.	.	44.3	24.9	35.3	17.1	15	161	33.5

	Nitrendipine	88	63.9	84.9	37.5	20.1	54.4	32	26	219	55.3
Cholinergic	Hexahydro-sila-difenidol	88	63.2	40.0	30.5	63.9	50.8	17.1	26	233	48.5
Cyclic Nucleotide	BRL 50481	176	41.7	42.7	27.6	47.5	40.6	15.3	26	245	40.0
	Trequinsin	88	45.1	59.5	82.4	80.0	64.2	20.2	26	269	65.4
	Vinpocetine	33	40.1	29.9	24.8	53.8	37.6	18.7	26	245	38.8
Cytoskeleton and ECM	Vincristine	88	49.1	53.2	18.8	48.7	44.3	21.4	26	261	45.2
Glutamate	AMN082	33	56.0	45.8	25.7	59.7	48.3	23.5	26	234	47.0
	Eliprodil	88	68.7	51.2	51.7	74.3	62	19.6	26	252	59.9
Hormone	(R,R)-cis-Diethyltetrahydro-2,8-chrysenediol	88	33.7	23.3	64.6	67.5	44.6	27.3	26	233	40.3
	beta-Estradiol	33	58.4	36.6	37.9	59.1	48.7	22.8	26	250	44.4
	Cyproterone	176	46.5	55.1	8.2	19.5	35.2	25	26	255	36.9
	Danazol	33	52.2	32.0	24.2	46.5	40.1	20.6	26	238	39.1
K+ Channel	Psora-4	88	35.2	27.6	29.5	28.2	30.5	13.8	26	248	31.0
	Quinidine sulfate	33	64.1	27.1	51.6	41.6	46.5	21.8	26	218	45.9
Nitric Oxide	4-Phenyl-3-furoxancarbonitrile	88	37.3	64.6	35.6	21.3	40.6	21.2	26	224	41.5
Phosphorylation	7-Cyclopentyl-5-(4-phenoxyphenyl)-7H-pyrrolo[2,3-d]pyrimidin-4-ylamin	33	55.8	44.1	28.0	41.0	43.9	22.9	26	245	42.8
	Cyclosporin A	88	42.3	65.8	39.6	73.4	55.3	23.2	26	241	56.8
	DAPH	13	60.6	47.1	60.6	72.3	59.7	19.7	26	258	57.0
	Kenpaullone	88	27.0	30.2	24.4	32.9	28.7	19.4	26	244	27.5
	LFM-A13	88	0.0	27.1	21.7	22.8	16.7	16.6	26	209	15.8
	SU 4312	88	37.6	58.2	41.6	60.8	49.3	20.9	26	237	50.6
	Tyrphostin AG 1478	33	47.8	41.2	48.9	62.5	49.6	18.4	26	231	48.1
CONTROL	DMSO	0	47.7	32.7	37.9	44.0	40.9	19.5	361	3452	39.8

The mean % of animals alive was calculated in two different ways:,

* Mean % of animals alive (4 expts): Mean % survival calculated by averaging the mean survival determined in 4 different experiments.

Mean % of animals alive (all): Mean % survival calculated by pooling all the animals across experiments and dividing the surviving animals by the number of total animals. This number was used to calculate OSR (%) in table 1.

Supplementary Table 4.
Summary of compound screening data and subsequent analyses

Category	Parameter	Description
**Assay**	Type of assay	Lifespan
	Target	None, phenotype-based compound screen
	Measurement	Length of life in days
	Assay protocol	doi: 10.3791/2496
**Library**	Library size	1280 compounds, LOPAC library
	Library composition	55 pharmacological classes
	Source	Sigma
	Additional comments	Five of the original 60 classes were removed from the library annotation
**Screen**	Format	96-well
	Concentration(s) tested	33 uM
	Plate controls	DMSO, 0.33%
	Reagent/ compound dispensing system	Hydra
	Detection instrument and software	Microscopy, by eye
	Assay validation/QC	Reference screen, parametric model
	Correction factors	None
	Normalization	Relative to DMSO controls in each plate
	Additional comments	Lifespan data are not normally distributed, Z'-score does not apply. All primary data from the screen can be found in Supplementary Table 1
**Secondary analysis**	Hit criteria	Lifespan extension by 1% or more, FDR<0.66, P<0.05 to at least two adjacent concentrations in dose response experiments. All the primary data from the follow up lifespan experiments can be found in Supplementary Table 2
	Hit rate	4.7%* (see comments)
	Additional assay(s)	Oxidative stress resistance in *C.elegans*
	Confirmation of hit purity and structure	For each hit, an independent vial was purchased from Sigma or a different supplier and retested
	Additional comments	The high hit rate is likely caused by the libraries high representation of biologically active compounds and multiple compounds targeting some mammalian proteins..

Total numbers:

No. of *C.elegans* animals tested in lifespan assays:	126,681
No. of *C.elegans* animals tested in stress resistance assays:	17,257

Lifespan Screen:

These numbers represent the data set collected for the LOPAC lifespan screen.

No. of compounds tested:	1280
Pharmacological classes tested:	55
Concentration tested:	33 uM
Solvent:	DMSO
Final DMSO conc.:	0.33%
Compounds added at:	day 1 of adulthood
Total no. of animals:	60,921
No. of animals that died:	59,783 (98.1% of total)

Mean No. of animals/compound	41 +/- 7 animals (min: 30m, max: 58)
No. of DMSO control animals:	5026
No. of DMSO control populations:	128 (5026 animals total)
No. of compound treated populations:	1280
Mean lifespan of control:	21.1 +/- 0.7 days
Criteria of a primary hit:	% change>1% , P<0.05, FDR<0.66
Primary hits:	156

Reference Screen:

To experimentally the determine sensitivity and selectivity of the screen a reference screen comprising of 128 populations was conducted. One researcher treated selected a number of populations (6) and treated them with the lifespan extending compound mianserin instead of DMSO. The number and locations of the mianserin treated populations was unknown to the researcher conducting the reference screen.

No of compounds tested:	Mianserin
Pharmacological classes tested:	--
Concentration tested:	33 uM
Solvent:	DMSO
Final DMSO conc.:	0.33%
Compounds added at:	day 1 of adulthood
Total no. of animals:	4994
No. of animals that died:	4899 (98.1% of total)

Mean No. of animals/ compound:	39.02 +/-6.6
No. of DMSO control populations:	122 (4759 animals total)
No. of Mianserin treated populations:	6 (235 animals total)
No. of Ref. screen populations:	128
Mean lifespan of control:	21.1 +/- 2.3 days
Criteria of a hit:	% change>1% in lifespan , P<0.05, FDR<0.66
True positives:	6
False positives:	2

Post-screen lifespan assays, secondary testing:

No. of compounds tested:	156 primary hits
Concentration tested:	3, 10, 33, 88 and 176 uM
Solvent:	DMSO
Final DMSO conc.:	0.33%
Compounds added on:	day 1 of adulthood
Total no. of animals:	65,760
No. of animals that died:	64,437 (97.9% of total)

Oxidative-stress resistance in *C.elegans:*

No of compounds tested:	57
Pharmacological classes tested:	15
Concentration tested:	optimized concentration for lifespan extension
Solvent:	DMSO
Final DMSO conc.:	0.33%
Compounds added at:	day 1, paraquat added on day 5 (100mM final)

Total no. of animals:	17,257
Mean No of animals/compound:	247 +/- 23 animals (min: 161, max: 301)
No. of DMSO control animals:	3452
No. of DMSO control populations:	361
No. of compound treated populations:	1451
